# 1,2-Octanediol, a Novel Surfactant, for Treating Head Louse Infestation: Identification of Activity, Formulation, and Randomised, Controlled Trials

**DOI:** 10.1371/journal.pone.0035419

**Published:** 2012-04-16

**Authors:** Ian F. Burgess, Peter N. Lee, Katrina Kay, Ruth Jones, Elizabeth R. Brunton

**Affiliations:** 1 Medical Entomology Centre, Insect Research & Development Limited, Cambridge, United Kingdom; 2 PN Lee Statistics and Computing Ltd, Sutton, United Kingdom; 3 Infection control, NHS Leeds, Leeds, United Kingdom; 4 South West Wales Regional Office, Care and Social Services Inspectorate Wales, National Assembly of Wales, Wales, United Kingdom; Johns Hopkins Bloomberg School of Public Health, United States of America

## Abstract

**Background:**

Interest in developing physically active pediculicides has identified new active substances. The objective was to evaluate a new treatment for clinical efficacy.

**Methods and Findings:**

We describe the selection of 1,2-octanediol as a potential pediculicide. Clinical studies were community based. The main outcome measure was no live lice, after two treatments, with follow up visits over 14 days.

Study 1 was a proof of concept with 18/20 (90%) participants cured.

Study 2 was a multicentre, parallel, randomised, observer-blind study (520 participants) that compared 0.5% malathion liquid with 1,2-octanediol lotion (20% alcohol) applied 2–2.5 hours or 8 hours/overnight. 1,2-octanediol lotion was significantly (p<0.0005) more effective with success for 124/175 (70.9%) RR = 1.50 (97.5% CI, 1.22 to 1.85) for 2–2.5 hours, and 153/174 (87.9%) RR = 1.86 (97.5% CI, 1.54 to 2.26) for 8 hours/overnight compared with 81/171 (47.4%) for malathion.

Study 3, a two centre, parallel, randomised, observer-blind study (121 participants), compared 1,2-octanediol lotion, 2–2.5 hours with 1,2-octanediol alcohol free mousse applied for 2–2.5 hours or 8 hours/overnight. The mousse applied for 8 hours/overnight cured 31/40 (77.5%), compared with 24/40 (60.0%) for lotion (RR = 1.29, 95% CI, 0.95 to 1.75; NNT = 5.7) but mousse applied for 2–2.5 hours 17/41 (41.5%) was less effective than lotion (RR = 0.69, 95% CI, 0.44 to 1.08).

Adverse events were more common using 1,2-octanediol lotion at both 2–2.5 hours (12.0%, p = 0.001) and 8 hours/overnight (14.9%, p<0.0005), compared with 0.5% malathion (2.3%). Similar reactions were more frequent (p<0.045) using lotion compared with mousse.

**Conclusions:**

1,2-octanediol was found to eliminate head louse infestation. It is believed to disrupt the insect's cuticular lipid, resulting in dehydration. The alcohol free mousse is more acceptable exhibiting significantly fewer adverse reactions.

**Trial registrations:**

Controlled-Trials.com ISRCTN66611560, ISRCTN91870666, ISRCTN28722846

## Introduction

During the 1990s head lice in many countries were found to have acquired resistance to treatment using insecticides, resulting in treatment failures, longer term infestations, and repeated cycles of treatment. Until about 2005 alternative treatment options were limited and, although physically acting preparations are now available, insecticides remain in use in many countries.

Currently used physically acting preparations are mostly siloxanes or lipids with a low surface tension that enables them to enter and block the louse spiracles resulting in immobilisation of the insects and disruption of water balance [1]. However, the respiratory tract is not the only vulnerable point for physically acting chemical compounds. Lice, like all insects, are coated with lipid, which has waterproofing and protective functions for the chitin cuticle. Dissolution of an insect's lipid coat can also disrupt its ability to minimise water loss through the cuticle and is likely to affect the normal functioning of the cuticle in other ways [2], [3].

Surface active agents of various kinds are applied to the hair and scalp, the most common of which are in toiletry shampoos and conditioners. Some of these are believed to disrupt surface lipids leading to insect death, but none has previously been commercially exploited.

We conducted preclinical screening of a range of alkyl diols, used as wetting and spreading agents in toiletries, for activity against human lice. We found 1,2-octanediol (caprylyl glycol) had the best characteristics of activity, safety, and history of prior human use. We have evaluated this material in randomised, controlled, observer blinded clinical trials randomised by individual, the preferred approach in scientific advice from the competent authority. First, 1,2-octandiol was compared with 0.5% malathion, a standard of care treatment widely prescribed in the UK. Subsequently, an alcohol free version was compared with the original preparation.

## Materials and Methods

### Preclinical work

Preclinical screens of candidate active substances were conducted using laboratory reared clothing/body lice (*Pediculus humanus*) as test insects. Adult lice were fed during the morning before testing and allowed to eliminate excess water from the bloodmeal over a period of approximately 4 hours before exposure to treatments in a manner described in previous studies [4]. Later tests conducted using formulated materials were performed using head lice collected from children screened under a separate ethical approval from the clinical investigations. Louse eggs were obtained by providing fecund adult laboratory reared clothing lice with a close-spaced nylon gauze substrate over a period of 48 hours.

Test materials were dissolved in 50% propan-2-ol. Lice or louse eggs were dipped in the test solution for 10 seconds, blotted of excess fluid, and then incubated overnight under normal maintenance conditions (30°±2° Celsius and 50%±15% relative humidity). The effects of the materials on lice were recorded without washing but the gauze squares with louse eggs were washed using a non-medicated, non-conditioning, frequent use shampoo (Boots Frequent Wash Shampoo) diluted 1∶15 with warm tap water. The eggs were then blotted dry and incubated until all nymphs from the control batches of eggs had emerged [4].

### Clinical investigations

The protocols for the two randomised, controlled trials and supporting CONSORT checklist are available as supporting information: see Checklist S1 and Protocol S1 (Study 2) and Protocol S2 (Study 3).

#### Participants

We conducted three clinical studies that recruited participants via local newspaper and radio advertising. Families responding to advertising were sent a participant information booklet (PIB) by post. Those who wished to enrol telephoned the study coordinator to request a home visit, which usually occurred within 24 hours. Trained investigators used a standard protocol to examine participants for head lice using a plastic detection comb (“PDC”, KSL Consulting, Denmark). If lice were found, and the participant was eligible, a signed consent and assent procedure was followed. Other household members were offered examination and invited to join if eligible.

All enrolled participants provided baseline data on age, gender, hair characteristics, and previous pediculicide use. The lower age limit was 4 years in the proof of concept and first controlled study, there being no upper limit. For the second controlled study the lower age limit was 6 months, in conformity with the instructions for use of the product as a medical device. Treatments and assessments were conducted in the home. No payment was offered for participation.

Eligibility and inclusion and exclusion criteria were essentially as in our previous studies [5]–[7]. All participants were confirmed as having live lice present at the time of enrolment and had not used another pediculicide within 2 weeks of commencement. Participants confirmed they had no known sensitivity to any product components, were not using medication for asthma, had not been treated using co-trimoxazole within 4 weeks, and had not participated in another clinical study within 30 days. Investigators also checked for secondary infection of the scalp and confirmed that prospective participants were not pregnant or nursing mothers.

#### Ethics

Ethical approval for the proof of concept study (protocol CTEP01, ISRCTN66611560) was granted by Hertfordshire 2 Research Ethics Committee (EudraCT 2005-005512-24). Approval for the first randomised controlled study (protocol CTEP02, ISRCTN91870666) was granted by Trent Research Ethics Committee (EudraCT 2007-001872-36), with locality issue ethical approvals granted by Cambridgeshire 2 REC, South West Wales REC, and Leeds (West) REC for the three study sites. Our second randomised controlled study (protocol CTMK12, ISRCTN28722846) was approved by Leeds West REC and locality issues by Cambridgeshire 2 REC.

At the first visit, people who wished to participate reported that they had read the PIB and understood the purpose and requirements of the study. Parents or guardians provided written consent for children younger than 16 years, who also gave written assent witnessed by the parent/guardian.

The studies were conducted in conformity with the principles of the Declaration of Helsinki, the International Conference on Harmonisation (ICH) Harmonised Tripartite Guideline for Good Clinical Practice (GCP), and of European Union Directives 2001/20/EC and 2005/28/EC.

#### Study medications

For the proof of concept (Study 1), we used a lotion containing 5% (w/v) 1,2-octanediol dissolved in 50% (v/v) propan-2-ol (isopropanol) and water.

Most people found this preparation had a pungent odour and caused skin irritation (see Adverse Events), so it was replaced for the first randomised study (Study 2) by 5% (w/v) 1,2-octanediol in a vehicle of 20% (v/v) propan-2-ol in water with 0.64% sodium dodecyl sulphate as a co-surfactant (prepared for EctoPharma Ltd, Selkirk, Scotland) supplied in 100 ml bottles. The comparison product was 0.5% malathion liquid in 200 ml bottles (Derbac-M liquid, SSL International, Manchester, UK). Both preparations were applied drop by drop and massaged into the hair to ensure full coverage of the hair and scalp.

Even with reduced alcohol (20%) the odour and irritation were more than desirable so, for the second randomised study (Study 3), we used an alcohol free lotion containing 5% w/v 1,2-octanediol in PEG 6 caprylic/capric glycerides and water (Thornton & Ross Ltd, Huddersfield, UK). The comparison product was the same 5% (w/v) 1,2-octanediol in 20% (v/v) propan-2-ol in water as used in Study 2. Each of the preparations was supplied in 150 ml finger pump bottles fitted with a moussing grid to produce a foam for easier control of the fluid on the hair.

In all three studies the investigators applied sufficient product to thoroughly moisten the hair and scalp. The treatment was repeated after one week. Participants were provided with shampoo and advised when to wash off the treatment. In Study 2 we provided carers with cards to record the actual time of washing, which were then sealed in envelopes until after the initial stage of data processing.

In the proof of concept study the lotion was applied for 8 hours or overnight. For Study 2 one cohort using 1,2-octanediol lotion was also treated for 8 hours or overnight (8–12 hours), a second used the same product for 2 hours (maximum 2.5 hours to allow some flexibility of timing within the home environment), and the third group using the 0.5% malathion liquid washed the product off after an overnight application.

In Study 3, the three treatment groups were 5% 1,2-octanediol with 20% isopropanol applied for 2–2.5 hours; 5% 1,2-octanediol alcohol free applied for 2–2.5 hours; and 5% 1,2-octanediol alcohol free applied for 8 hours or overnight.

#### Objectives

After Study 1 had confirmed that 1,2-octanediol lotion was able to eliminate a head louse infestation, Study 2 was designed with the objective of comparing the efficacy of 5% 1,2-octanediol lotion and 0.5% malathion liquid with a power sufficient to detect superiority of activity of either product but also to identify whether there were any safety issues in relation to the preparation in use in a wider population.

Study 3 was designed to demonstrate the activity of 1,2-octanediol to kill head lice in the absence of alcohol.

#### Outcomes

For all three studies the primary outcome measure was elimination of head lice using two applications of treatment 7 days apart. Initial diagnosis was by dry detection combing, using the “PDC” comb. We followed up participants in all studies using the same method on days 2, 6, 9, and 14 in Studies 1 and 2, and on days 1, 6, 9, and 14 for Study 3. Combing to determine the success or otherwise of treatment was performed systematically over the whole scalp, but was limited to 2–3 strokes of the comb on each section in order to minimise the intervention aspect of combing. The combing examination on day 14 was more extensive to ensure no lice were present. In all studies, “Cure” was defined as no lice after the second application of treatment, on days 9 and 14.

As in previous studies [5]–[7] we used a rigorous algorithm defining the criteria for reinfestation as a) no adult lice or third stage nymphs present following the first treatment; and b) no more than two adult lice or third stage nymphs found by combing on days 9 and 14. Any participant not fitting into either the cure or reinfestation after cure criteria was categorised as a treatment failure.

#### Sample size

The sample size determination used for the proof of concept (Study 1) planned to provide an estimate of cure rate expected in a controlled study and to determine whether 5% 1,2-octanediol was comparable to existing products with a cure rate of at least 50%. A group size of 20 narrowed the precision of the anticipated efficacy to within 10%, and gave confidence (p≤0.048) that an observed cure rate of 50% or lower would be unlikely if the underlying cure rate was 70% or greater.

Study 2 evaluated three treatment arms, two dose regimens of 5% 1,2-octanediol lotion with 20% alcohol, each separately compared with malathion liquid. Based on previous experience [7] we anticipated the efficacy of 0.5% malathion liquid at about 35% with an expected efficacy for 1,2-octanediol lotion to exceed this by at least 25%. Using a one-tailed alpha of 2.5% we estimated that 126 participants per group would provide 95% power to detect superiority. However, for poorer outcome, say a superiority of 20% we estimated that approximately 163 per group would provide 90% power. Therefore, to allow for early withdrawals and other non-evaluable cases, the group size was set at 170 per arm or a minimum of 510 participants over three investigation sites.

For Study 3 the aim was to confirm that the 5% 1,2-octanediol alcohol free lotion (octanediol-AF) had efficacy essentially similar to that of 5% 1,2-octanediol with 20% alcohol (octanediol lotion). It was not designed as a full equivalence study but the group size of 40 allowed us to be able to identify, with 95% confidence and up to 90% power, a difference of 35% in the success rate at 14 days between the treatments in pair wise comparisons.

#### Randomisation – allocation concealment

For Study 1 the treatment allocation was open label.

Study 2 was conducted at three, geographically separate, UK study sites, in Cambridge, Leeds, and Swansea, each of which was supplied with sets of pre-numbered, anonymised, boxed, treatment kits in balanced blocks of twelve. Each investigator was issued with a complete block of treatment kits to be used in number sequence. Further blocks were issued as required but any unused packs were not reallocated at the end of the study. Each kit contained enough bottles to complete the two treatment applications for one participant (four 100 ml bottles of 5% 1,2-octanediol lotion or two 200 ml bottles of 0.5% malathion liquid). Treatment was allocated using the next available numbered kit held by the investigator and all kits from one block issued before starting a new block.

Randomisation was by individual so household members could receive different treatments. Therefore, in order to minimise the possibility of allocation bias within a family the lowest available numbered kit was allocated to the youngest participating household member, the next number to the next higher in age, and so on, until all participating members were allocated a treatment. Infested household members who were not eligible or declined participation were offered 4% dimeticone lotion (Hedrin 4% lotion, Thornton & Ross Ltd, Huddersfield, UK) as a standard of care treatment to minimise the risk of reinfestation within the household.

Study 3 was also randomised by individual. In this study a treatment allocation code was generated using the free online randomisation service provided at http://www.randomization.com, in 14 balanced blocks of 9 treatments. Treatment was determined from instructions contained in the next available sequentially numbered, sealed, opaque envelope taken from a block allocated to the investigator.

For both randomised studies, assessors were blinded to treatment allocation. Different members of the investigation team were assigned the task of post-treatment assessment from those applying treatment. Participants were asked not to provide information that would disclose the treatment used.

#### Statistical methods

As there was only one treatment group for the proof of concept study the only analysis was of the distribution of graded or semi-continuous variables within the group.

For the two randomised controlled studies, pair-wise comparisons were conducted between each of groups. In Study 2 analyses were conducted based on the whole population and also based on the youngest member of each household group, each with stratification for study centre, with unstratified analysis also conducted for each of the study centres. For stratified data the method of Mantel and Haenszel [8] was used to calculate relative rates, with the confidence interval estimated by the method of Greenland and Robins [9]. In both studies, presence/absence variables comparisons were made using both the Fisher exact and chi-squared tests. In some analyses a chi-squared result only was available because the number of cases was too large for the exact test to be conducted. For continuous or semi-continuous variables comparisons were made using Kruskal-Wallis analysis of variance (equivalent to the Mann-Whitney test for two group comparison).

## Results

### Preclinical work

Our investigation of several series of alkyl alcohols, using the concentration required to kill 50% of the insects (LC_50_) as the benchmark, found that vicinal diols exhibited significantly greater activity against lice than 1,3-diols and other non-vicinal diols, irrespective of carbon chain length [10]. For example, 1,2-decanediol was found to be significantly more active than other diols, triols and mono-ols (Tables 1 and 2).

**Table 1:** Comparison of the LC_50_ pediculicidal activity of vicinal and non-vicinal diols.

Compound	Diol type	LC_50_ value (mmol/l)	Potency index (%)*
1,2-Butanediol	Vicinal	>700	Not effective
1,3-Butanediol	Non-vicinal	>2000	Not effective
1,2-Hexanediol	Vicinal	>200	6
1,5-Hexanediol	Non-vicinal	Not calculable	Not effective
1,6-Hexanediol	Non-vicinal	Not calculable	Not effective
2,5-Hexanediol	Non-vicinal	Not calculable	Not effective
1,2-Octanediol	Vicinal	40	30
1,3-Octanediol	Non-vicinal	>400	Not effective
1,2-Decanediol	Vicinal	12	100
1,3-Decanediol	Non-vicinal	40	30

* The potency index compares the observed LC_50_ value with that of 1,2-decanediol ( = 100%).

**Table 2:** Comparison of the LC_50_ pediculicidal activity of vicinal diols with triols and mono-ols.

Compound	LC_50_ value (mmol/l)	Potency index (%)*
1,2,3-Hexanetriol	Non calculable	Not effective
1,2,6-Hexanetriol	Non calculable	Not effective
2-Ethyl-1-hexanol	300	4
2-Ethyl-1,3-hexanediol	60	20
1-Decanol	35	32
2-Decanol	37	34
4-Decanol	183	6
9-Decene-1-ol	33	36
1,2-Decanediol	12	100
3-Ethyl-1-decanol	62	20
1-Octyl-1,2-decanediol	97	12
3-Ethyl-1,2-decanediol	Non calculable	Not effective

* The potency index compares the observed LC_50_ value with that of 1,2-decanediol ( = 100%).

**Figure 1:**
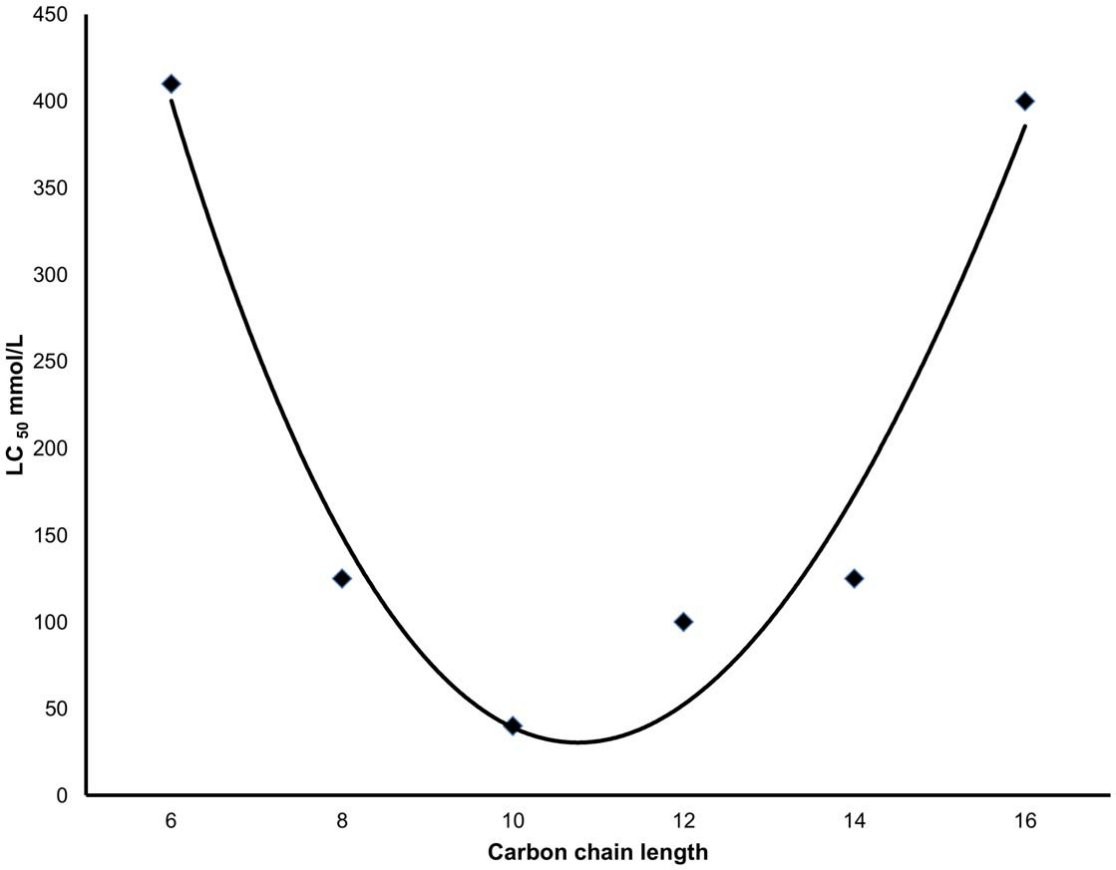
Effect of diol chain length on the activity against human lice, *Pediculus humanus*. Activity of vicinal diols was found for molecules with carbon chains C4–C16. Activity, as shown by concentration required to kill 50% of insects (LC_50_), increases with chain length from C4 through to C10 with a reduction of activity through C12 to C16.

**Figure 2:**
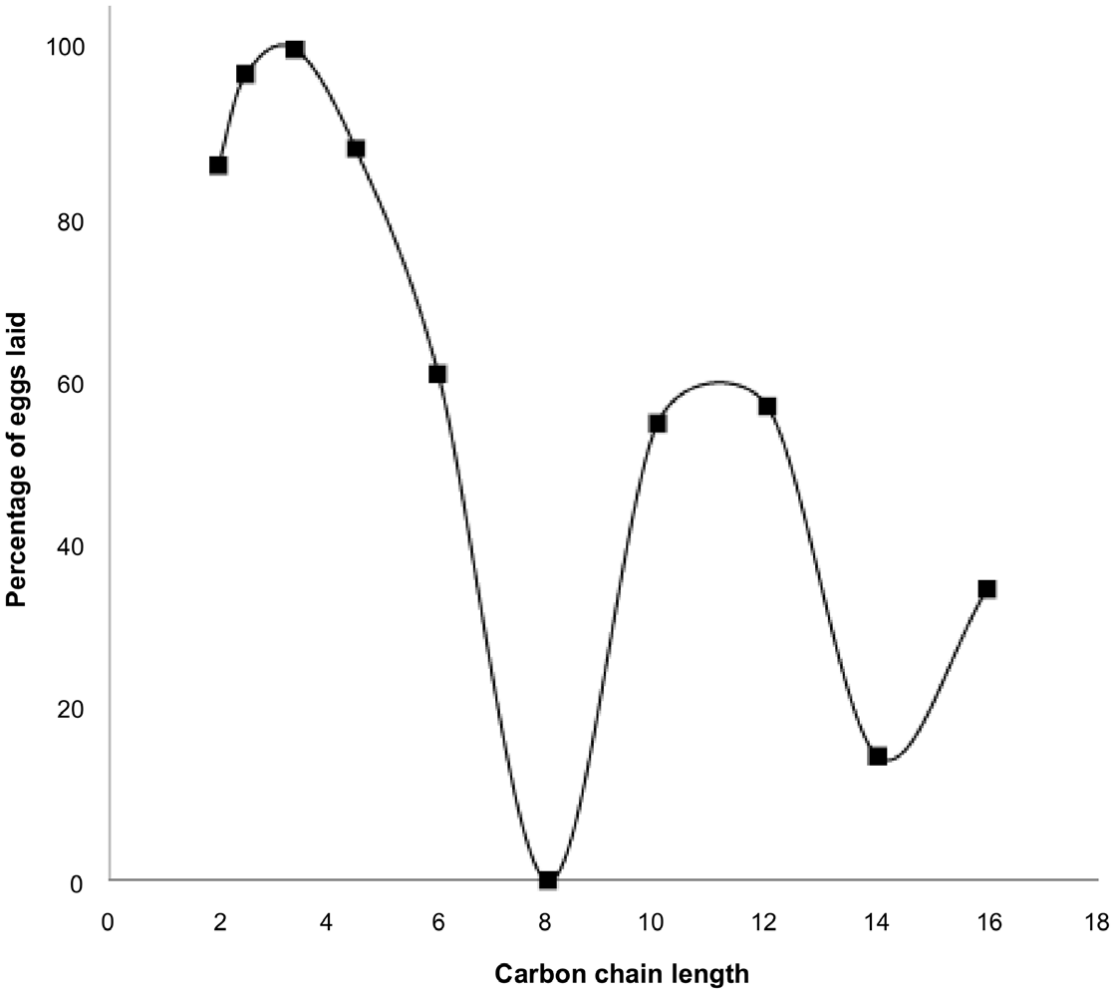
Effect of diol chain length on the percentage of eggs laid by treated lice. The activity of vicinal diols against lice is relatively slow, taking several hours for the insects to become fully immobilised. During this time some lice remain capable of laying eggs. It was found that all vicinal diols, with the exception of 1,2-octanediol allowed a proportion of lice to lay eggs subsequent to treatment.

When comparing vicinal diols it was found that they exhibited activity over a range of molecular sizes from C_4_ to C_16_ but the most active to kill lice, using a plot of LC_50_ values, were 1,2-octanediol, 1,2-decanediol, and 1,2-dodecanediol (Table 1, Figure 1) [10]. However, because these chemicals exert no obvious physiological activity, the effects on adult lice are not immediate and some insects survive long enough to lay eggs subsequent to treatment, which could be an important factor in relation to possible reinfestation. Consequently, inhibition of egg production could determine the usefulness and efficacy of the active material. Counting the eggs laid by each batch of treated lice showed that 1,2-octanediol completely inhibited egg laying, whereas a few of the insects treated using other vicinal diols did lay some eggs (Table 2, Figure 2) [10]. These eggs were not affected by the dried film of diol on the substrate and could survive to start a new infestation. However, when louse eggs were exposed to solutions of those vicinal diols found to be most active against lice, and applied in the same manner as to lice, none of the louse eggs developed to the point of hatching.

### Clinical investigations

#### Participant flow

In summer 2006, we enrolled 22 people from 12 families in Study 1. Two were non-compliant and washed off the treatment early, so we enrolled two additional people to fulfil the protocol requirement for 20 evaluable participants.

**Figure 3:**
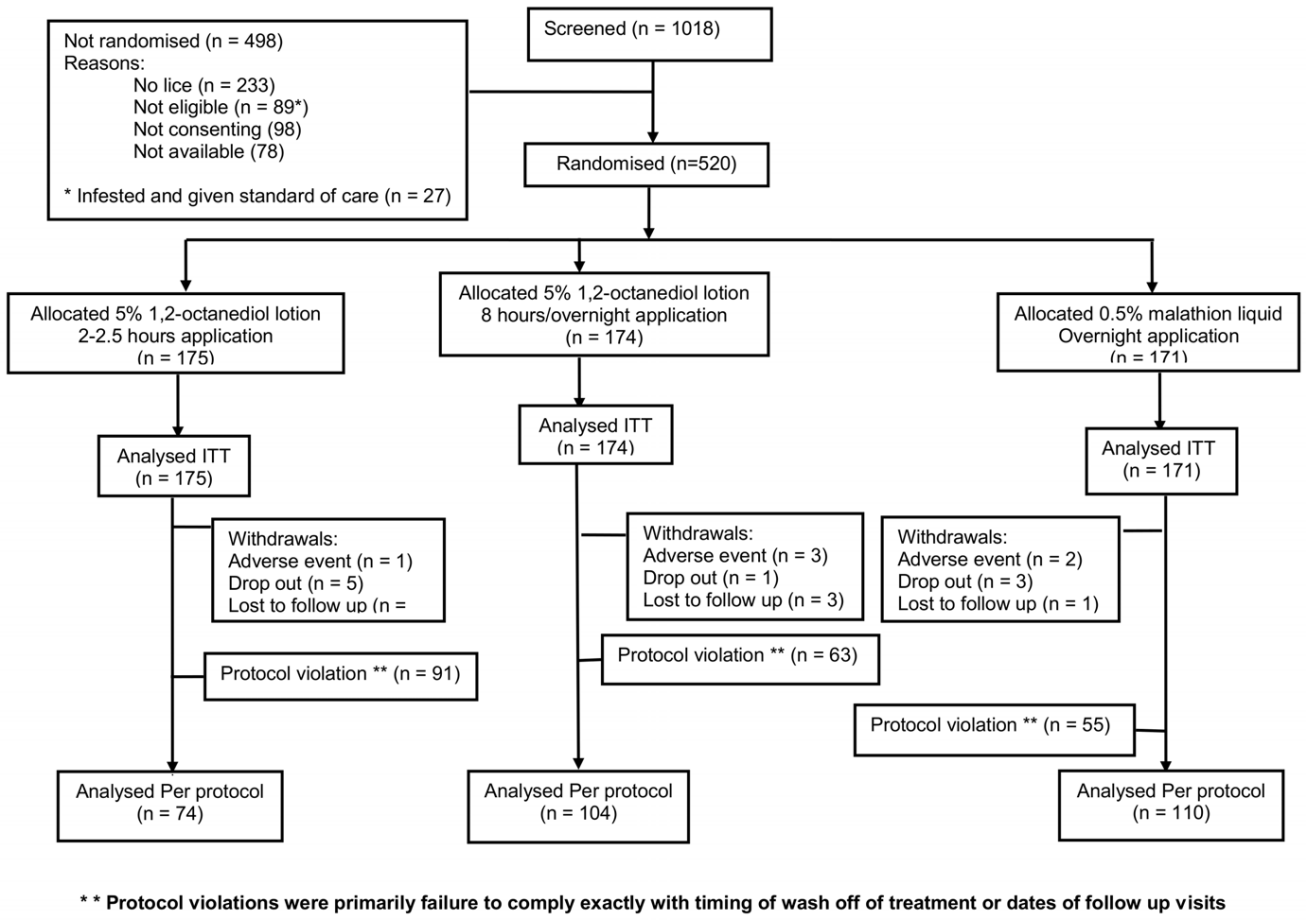
Flowchart of participants through Study 2. CONSORT flowchart of participants through Study 2. There was a large difference in numbers analysed by intention to treat (ITT) and per-protocol (PP) mainly due to a specific set of protocol violations indicated by (**). These protocol violations were primarily failure to comply exactly with timing of wash off of treatment or dates of follow up visits.

In Study 2, we recruited 520 participants from 275 families through three study centres: 180 in Cambridge, 166 in Leeds, and 174 in Swansea between October 2007 and May 2008. Of these, 175 were randomised to a 2 hour treatment and 174 to the 8 hour or overnight treatment, both using octanediol lotion, and 171 to 0.5% malathion liquid. Twenty-three participants did not complete the study: six left following adverse events, 9 dropped out, and 8 who were lost to follow up (Figure 3). However, most participants received two applications of treatment with 7 days between and were assessed on days 2, 6, 9 and 14 after the first treatment application.

**Figure 4:**
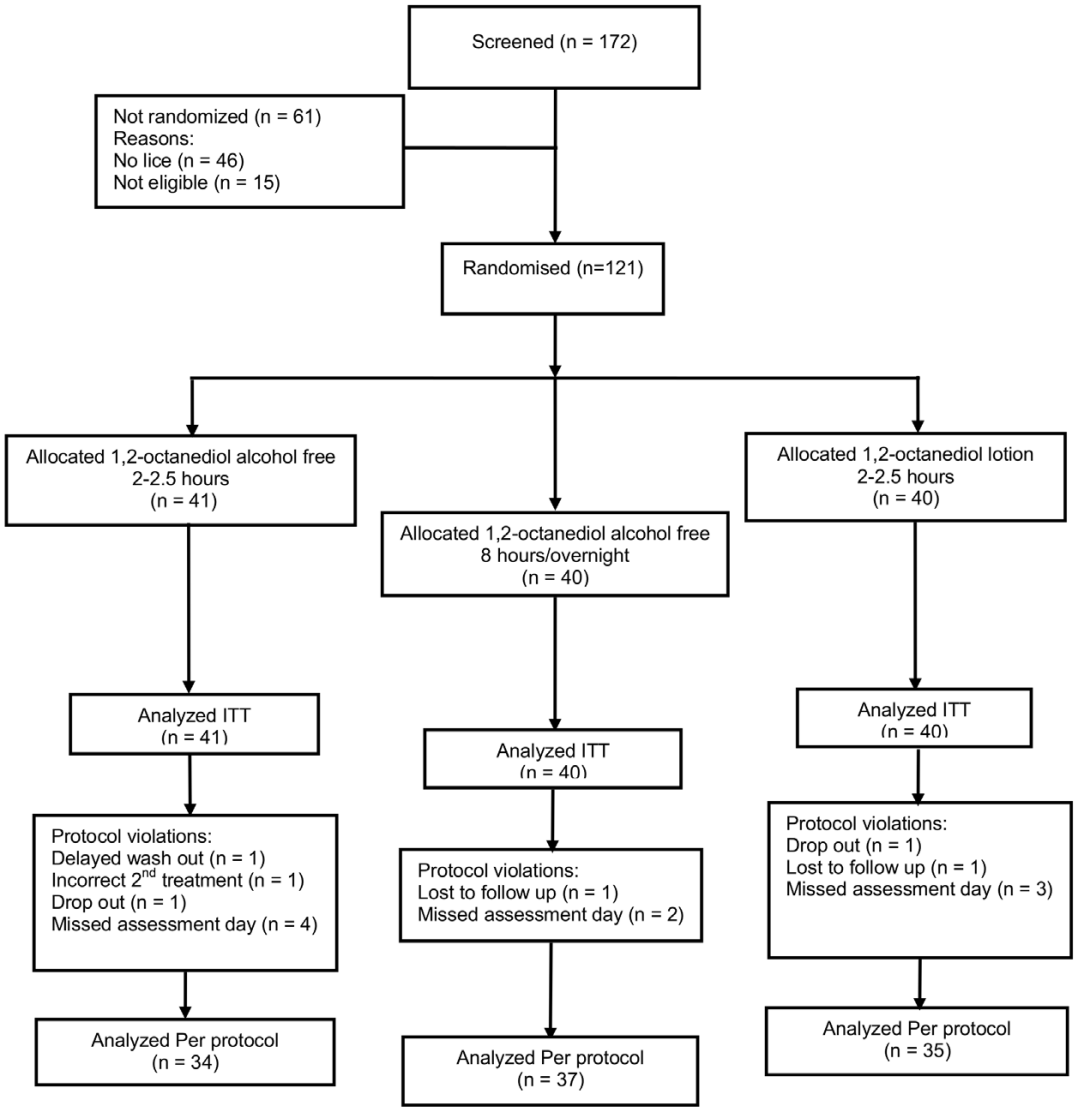
Flowchart of participants through Study 3. CONSORT flowchart of participants through Study 3. Although there few proportionately fewer protocol violations in the study they were essentially similar to those in Study 2: failure to keep appointments with only one participant in this study washing out the product at the wrong time.

In Study 3 we recruited 121 participants from 59 households, between October 2010 and March 2011, 97 in Cambridge and 24 in Leeds. Of these 40 were randomised to receive octanediol lotion for 2–2.5 hours, 41 to the 2–2.5 hour treatment and 40 to the 8 hour/overnight treatment with the octanediol-AF. Five participants did not complete the study: two dropped out, two were lost to follow up, and one received an incorrect second treatment (Figure 4). In this study participants were also treated twice with 7 days between and assessments were conducted on days 1, 6, 9, and 14.

#### Baseline data

In the 334 families enrolled in the two controlled studies, the mean household size was 4.52 and the number of participants enrolled ranged from 1 to 6 (mean 1.92) with no differences between groups. There was a non-significant trend towards larger household sizes and more people per household enrolled in Study 3 compared with Study 2.

In Study 2, the different groups showed no overall significant differences in demographic factors such as age, gender, hair length, hair thickness (density), degree of curl, or whether dry/greasy, or for intensity of initial infestation. However, some variations were identified between or within centres, e.g. in Leeds there was a significant (p = 0.019) overall group difference for age and the proportion of children younger than 10 was significantly greater in the malathion group compared with 1,2-octanediol applied for 8–12 hours (p = 0.01). Similarly, Swansea enrolled a high proportion of males with a significant between group difference for 1,2-octanediol applied for 2 hours compared with malathion (p = 0.01). The baseline characteristics of groups from the intention to treat population are summarized in Table 3. Similarly there were no significant differences between groups in Study 3, the only trend being the difference in average time since last treatment, which was twice as long in the group treated using octanediol-AF for 8 hours compared with octanediol-AF for 2 hours (Table 4).

**Table 3:** Demographic characteristics of the intention to treat population at baseline (Study 2).

Characteristic	Population	Octanediol 2 hours	Octanediol >8 hours	Malathion >12 hours	Total
Number of participants	All	175	174	171	520
	Cambridge	62	61	57	180
	Leeds	55	56	55	166
	Swansea	58	57	59	174
Age (<10)	All	49.1%	53.4%	56.1%	52.9%
	Cambridge	40.3%	54.1%	45.6%	46.7%
	Leeds	60.0%	50.0%++	67.3%	59.0%*
	Swansea	48.3%	56.1%	55.9%	53.5%
Sex (male)	All	24.0%	17.2%	20.5%	20.6%
	Cambridge	19.4%	18.0%	28.1%	21.7%
	Leeds	14.5%	16.1%	18.2%	16.3%
	Swansea	37.9%++	17.5%	15.3%	23.6%*
Family size (mean)	All	4.35	4.33	4.52	4.40
Participants per family (mean)	All	1.76	2.06	1.81	1.88
Hair length (below shoulders)	All	57.7%	63.8%	55.0%	58.8%
Hair thickness (thick)	All	36.0%	35.6%	34.5%	35.4%
Degree of curl (straight)	All	60.6%	57.5%	66.7%	61.5%
Hair type (normal)	All	84.0%	85.6%	86.6%	85.4%
Infestation (medium or heavy)	All	40.6%	45.4%	39.2%	41.7%
Days since last treatment (median)	All	55.0	52.0	60.0	56.0
Previous treatment successful	All	34.2%	31.2%	40.6%	35.4%

Key:

++ Two sided responses for 1,2-octanediol significantly greater (p<0.025) than for 0.5% malathion.

* Significant (p<0.05) variation between groups.

**Table 4:** Demographic characteristics of the intention to treat population at baseline (Study 3).

Characteristic	Population	Octanediol-AF 2 hours	Octanediol-AF >8 hours	Octanediol lotion 2 hours	Total
Number of participants	All	41	40	40	121
	Cambridge	33	32	32	97
	Leeds	8	8	8	24
Age (<10)	All	49.1%	53.4%	56.1%	52.9%
Sex (male)	All	19.5%	12.5%	27.5%	19.8%
Family size (mean)	All	5.50	5.52	5.50	5.51
Participants per family (mean)	All	2.44	2.41	2.45	2.44
Hair length (below shoulders)	All	46.3%	60.0%	45.0%	50.4%
Hair thickness (thick)	All	46.3%	42.5%	60.0%	49.6%
Degree of curl (straight)	All	51.2%	45.0%	67.5%	54.6%
Hair type (normal)	All	75.6%	80.0%	75.0%	76.9%
Infestation (medium or heavy)	All	63.4%	55.0%	60.0%	59.5%
Days since last treatment (median)	All	75.9	155.6	108.3	126.0*
Previous treatment successful	All	37.5%	32.5%	26.8%	32.2%

Key:

* Significant (p<0.05) variation between groups.

#### Outcomes

In Study 1, the proof of concept, the success rate using the outcome “cure” was 18/20 90.0%. Even on the most pessimistic assessment of success (after allowing for non-compliance) the true rate could be estimated at 68.4% with 95% confidence.

In Study 2, we compared participants for presence of live lice on each of days 2, 6, 9 and 14, with last value carried forward for any missing data. Day 2 analyses included 514 participants, and the remaining analyses 516 participants, because four people missed all assessments and two missed only the assessment at day 2.

In the intention to treat (ITT) population we saw cure, or reinfestation after cure, in 81/171 (47.4%) of participants treated with 0.5% malathion compared with 124/175 (70.9%) for 2–2.5 hours, and 153/174 (87.9%) for 8 hours/overnight treatments with 1,2-octanediol lotion. The overall relative rate (RR) versus malathion was 1.50 (97.5% CI, 1.22 to 1.85) (NNT = 4.26) for 1,2-octanediol 2–2.5 hours and 1.86 (97.5% CI, 1.54 to 2.26) (NNT = 4.19) for 1,2-octanediol 8 hours/overnight.

Over the course of the study 119/171 (69.6%) participants treated with malathion had lice at some stage after treatment compared with 72/174 (41.4%) people treated with 1,2-octanediol 8 hours/overnight. Similarly, the mean level of infestation on malathion treated participants (27.9 lice) was greater than on those treated using 1,2-octanediol for 8 hours/overnight (3.7 lice). The treatment success rate using malathion was higher in Swansea 34/59 (57.6%) compared with Cambridge 24/57 (42.1%) and Leeds 23/55 (41.8%), indicating differences in sensitivity to the insecticide.

At all time points (4 assessment days), and for all endpoints (live lice, total number of lice removed, numbers of adult males, numbers of adult females, and numbers of stage 1, stage 2, and stage 3 nymphs), lice were most frequent for malathion and least frequent for 1,2-octanediol 8 hours/overnight, with 1,2-octanediol 2–2.5 hours being intermediate. Overall variation between the groups and the difference between 1,2-octanediol 8 hours/overnight and malathion was highly significant (<0.0005) in all major analyses. The difference between 1,2-octanediol 2 hours and malathion was also significant at p<0.0005 for 17 of the 28 endpoint/day combinations studied. All the day 9 and 14 analyses were significant at least at p<0.025.

Outcomes for the per-protocol (PP) analyses were essentially similar to those for the ITT groups. After exclusion of 23 people who did not complete the study, two non-compliers, and those who either could not meet the assessment schedule or where the time of treatment wash off was incorrect or could not be calculated, 288 participants (74, 1,2-octanediol 2–2.5 hours; 104, 1,2-octanediol 8 hours/overnight; and 110, malathion) were analysed for the PP groups. Overall the relative rate of cure for 1,2-octanediol 2–2.5 hours (58/74) versus malathion (52/110) was 1.70 (97.5% CI, 1.31 to 2.22) and for 1,2-octanediol 8 hours/overnight (93/104) was 1.92 (97.5% CI, 1.50 to 2.44) (Table 2).

The comparison of the 275 youngest family members: 84 allocated to 1,2-octanediol 2–2.5 hours, 98 to 1,2-octanediol 8 hours/overnight, and 93 to malathion avoided issues of inter-correlations between members of the same family but reduced power. In this cohort no baseline between-group differences were significant at p<0.025. The only significant difference (p = 0.050) was for the outcome success of the most recent previous treatment, where the rates were 25.9% for 1,2-octanediol 8 hours/overnight and 40.3% for malathion. For the youngest member analysis, the relative success rate of treatments was 1.61 (97.5% CI, 1.16–2.23) for 1,2-octanediol 2–2.5 hours, 57/84 (67.9%), versus malathion 39/93 (41.9%) (p = 0.001); and for 1,2-octanediol 8 hours/overnight, 87/98 (88.8%), versus malathion the rate was 2.10 (97.5% CI, 1.58–2.79) (p<0.0005).

For Study 3, we conducted similar analyses to those used for Study 2 with the exception of youngest in family. For the ITT population, we found cure or reinfestation in 24/40 (60.0%) of those treated with 1,2-octanediol lotion (with 20% isopropanol) 2–2.5 hours, 17/41 (41.5%) in the group treated with alcohol free 1,2-octanediol 2–2.5 hours, and 31/40 (77.5%) for alcohol free 1,2-octanediol lotion 8 hours/overnight. In this study, the overall RR for alcohol free 1,2-octanediol 2–2.5 hours versus 1,2-octanediol lotion 2–2.5 hours was 0.69 (95% CI, 0.44 to 1.08), and for alcohol free 1,2-octanediol 8 hours/overnight versus 1,2-octanediol lotion 2–2.5 hours was 1.29 (95% CI, 0.95 to 1.75), which showed that leaving the alcohol free formulation on overnight significantly (p = 0.0021) improved the efficacy compared with using it for only 2–2.5 hours. However, there was no significant difference between 1,2-octanediol lotion (with 20% isopropanol) 2–2.5 hours and alcohol free 1,2-octanediol 8 hours/overnight.

In this study 15 people were excluded from the PP analysis: four early withdrawals and 11 who missed appointments or did not comply; leaving 34 participants in the alcohol free 1,2-octanediol 2–2.5 hours group, 37 in the 1,2-octandiol alcohol free 8 hours/overnight group, and 35 people treated using 1,2-octanediol lotion. The relative cure rates compared with 1,2-octanediol lotion (23/35) were 0.72 (95% CI, 0.47 to 1.10) for alcohol free 1,2-octanediol 2–2.5 hours (16/34) and 1.23 (95% CI, 0.93 to 1.64) for alcohol free 1,2-octanediol 8 hours/overnight (30/37).

#### Adverse events

In Study 1, adverse events were all application site events, generally stinging sensations of moderate severity, which the adverse events monitors concluded were probably caused by the high level of alcohol in the preparation. In the comparative studies, treatment related adverse events were also primarily application site reactions in which participants experienced some form of short term irritation, variously described as an itchy and “hot” paraesthesia-like sensation. Such events all passed within 15–20 minutes.

In Study 2 we recorded 103 adverse events in 68 participants, with 45 people having one event, 14 having two, six having three and three people having four events. The all-subject analyses showed significantly more participants experienced a treatment related adverse event after using 1,2-octanediol lotion with 20% alcohol at either 2–2.5 hours (21 = 12.0%, p = 0.001) or 8 hours/overnight (26 = 14.9%, p<0.0005), compared with 0.5% malathion liquid (2 = 2.3%). There were no serious adverse events associated with treatment. Two serious adverse events (SAEs) were reported, both in the group receiving 1,2-octanediol applied for 8–12 hours, in both cases the participants were involved in road traffic crashes, neither of which was related to study treatment.

In Study 3, a total of 39 adverse events were reported and no SAEs. Of these 21 events in 18 participants were considered possibly or probably related to treatment. Fifteen of these (12 participants) were following treatment with the original 1,2-octanediol lotion with 20% isopropanol. Those receiving alcohol free 1,2-octanediol mousse for 2–2.5 hours reported 4 events in the same number of people (p = 0.045) and only two participants (p = 0.008) in the alcohol free 1,2-octanediol mousse 8 hours/overnight group reported one adverse event each. This gave a RR of 0.2 (95% CI, 0.08 to 0.48) (NNH = −3.3) for experiencing any application site event using the alcohol free formula, irrespective of application time compared with the lotion containing 20% alcohol.

## Discussion

We have investigated a group of surface active chemicals with potential to disrupt insect cuticular lipids in treating head louse infestation. We found that 1,2-octandiol, which is commonly used in cosmetics and toiletries as a wetting and spreading agent, had the greatest overall activity. This material was evaluated in three clinical studies and in one of these was found to exhibit superior activity to 0.5% malathion. Since conducting these clinical investigations, further work has been performed to confirm that the mechanism of action involves disruption of components of the lipid coating of the louse (see Supporting Information files: Text file S1, Table S1, Table S2, Figure S1, Figure S2, Figure S3).

### Explanation of results

Most liquid preparations registered as Class I medical devices for elimination of louse infestation claim to obstruct the respiratory system [1], [5]–[7]. This is highly effective if the whole insect is coated and the preparation is able to penetrate all spiracular openings. However, if insufficient treatment is applied to thoroughly coat the hair and scalp, some lice can survive. This risk of “under dosing” is potentially even greater for louse eggs as the structure of the aeropyle openings of the eggshell cap restricts fluid penetration and blockage of the airway. Consequently, a physically acting treatment that does not depend on blockage of the respiratory system for its activity offers an attractive therapeutic alternative.

In order to exhibit activity against insects, a surface active material must be able to disrupt the water protective coating of the insect. Insect cuticular lipids vary from species to species but in most cases mainly comprise series of *n*-alkanes, with varying additional components such as *n*-alkenes, methylated alkanes, and secondary alcohols. The molecular size of these lipids mostly ranges from around 12 to 40 carbons in chain length depending upon species [2], [3], [11]. With such a wide range of molecular size it is unlikely that any single material would dissolve or disrupt the majority of the components but short chain alkanes could be readily displaced or dissolved by surfactants of similar molecular size.

It has long been recognised that some mono-alcohols can kill lice and it has been shown that activity increases with chain length over the range 8 to 12 carbons [12]. Longer chain alcohols also exhibit some activity but this is less simple to identify or quantify as those with carbon chain lengths of 14 and above can only be administered as emulsions. It appears from our *in vitro* investigations that the addition of a second alcohol moiety increases the activity of most medium chain alcohols, compared with mono-ols, and that this is most effective if placed in the 2-position on the molecule. Moving the second –OH group to the 3-position, as in non-vicinal diols, reduces activity significantly [10].

Our observations of the effects of structural activity in vicinal diols now need to be further evaluated in relation to the constitution of the cuticular lipid of human lice. In general terms the explanation is likely to be that the diol solution is able to emulsify elements of cuticular lipid and compromise the ability of lice to prevent water loss through the cuticle. This may seem strange in an insect that apparently needs to actively excrete water [1] but, in terms of overall water management, even active excretion is controlled to some degree by the insect whereas water loss through a cuticle that is compromised is not only uncontrolled but may accidentally result in the insect losing water at a rate too great for it to maintain its water integrity, resulting in rapid onset of dehydration. In the case of louse eggs, the activity is more difficult to interpret but, dissolved in the right vehicle, the 1,2-octanediol could be carried into the structure of the aeropyles to disrupt the lipid coat protecting the chorionic membrane surrounding the developing embryo, with resulting dehydration. This mode of action is unlikely to be affected by resistance.

### Comparison with previous studies

Hitherto surface active chemicals have not been investigated for their specific activity to treat head louse infestation. Consequently, it is not possible to make a direct comparison between these results and others. Other physically acting preparations, such as dimeticone [5], [7] and fatty acid esters [6] have produced similar results overall, and the differences between the efficacy of these physically acting materials and currently used insecticides were essentially similar to the results we found when comparing 1,2-octanediol with malathion. [6], [7] Our larger randomised study has provided not only a substantial body of evidence for the activity of 1,2-octanediol but also has confirmed the relative inefficacy of 0.5% malathion liquid in geographically widely separated sites, two of which have only previously been investigated using *in vitro* bioassays. [7], [13], [14] Malathion was selected for comparison because it was still recommended for prescribers by the British National Formulary at the time, despite evidence of resistance [13]; although our data from Swansea appear to confirm the suggestion [14] that prevalence of resistance to the insecticide may be lower in Wales than in Leeds and Cambridge. Malathion was also selected because it was, at the time, considered a “gold standard” in several countries in Western Europe, Australia, and in North America. No other insecticides were available in the UK apart from permethrin, which is even more affected by resistance problems than malathion, because materials such a carbaril and lindane were not only no longer available but also so little used in other territories that any results in comparison with them would have little value for most clinicians.

### Advantages of 1,2-octandiol as a pediculicide

Use of alcohols (ethanol or isopropanol) in the solvent vehicle for pediculicides has a long history, partly because they made formulation relatively easy and partly because it was believed they contributed towards the activity of the preparation. The negative effects of alcohol are flammability and irritation of the application site, especially on excoriated skin, yet several widely used products are still based on alcohol in Europe (Prioderm lotion), USA (Ovide), and Australia (KP24 lotion). In the development of the 1,2-octanediol product, isopropanol was first used for the reasons stated above but in clinical use it was clearly unsuitable as a vehicle due to the level of side effects incurred. Unlike conventional insecticides, which are all hydrophobic, 1,2-octanediol is partially hydrophilic and mixes into water with a little co-solvent assistance. We found that the resulting mixture was not quite as effective as when alcohol was included but this was outweighed by the greater acceptability and complete lack of flammability.

For all three formulations we have tested it was necessary to apply the product a second time because not all louse eggs were killed by the first treatment. Of course, the majority of consumers would prefer a treatment that required just one application but even the most effective preparations applied thoroughly can sometimes fail to kill one or two eggs, mostly through incomplete coverage. By making the alcohol free 1,2-octanediol product as a controllable mousse some issues with application technique were addressed. As with conventional insecticides, we showed that leaving the product on the hair for longer improved activity. Leaving 1,2-octanediol on the hair to allow it longer to act is much safer than doing this with neurotoxic compounds, especially carbaril, and lindane, and, combined with its relatively unobtrusive appearance and lack of odour could make it more acceptable to both children and their care givers.

### Strengths and weaknesses of the studies

Recruitment to these studies, as with all our clinical studies, was by response to advertising and as a result a group of participants with infestations that were more difficult to treat by conventional preparations may have been selected. However, such problems are only likely to have influenced the outcome of treatment using conventional insecticide, for example malathion resistance has been known in Cambridge since 1996 [15] and in Leeds since at least 2002 [13] and in both localities the intensity of resistance has increased rather than diminished (IF Burgess and K Kay, unpublished data). Problems with resistance to malathion would not, and did not, have any impact on the efficacy of the non-neuroactive 1,2-octanediol, demonstrating that this type of physically acting material is useful in treating infestations that are not responsive to conventional insecticide. In addition, our investigation method, in which each participant is followed up on four separate occasions in addition to the treatment days, has the advantage that it is rigorous in detection of treatment failure, although this again may result in some selection of participants who do not wish for this level of intervention in their routine.

### Conclusions and practice implications

Our observations of 5% 1,2-octanediol preparations show that it is an effective and well tolerated pediculicide. Where resistance to conventional insecticides is a potential problem, and for those people who dislike the oiliness of some other physically acting preparations, 1,2-octanediol, which appears to act by the novel mode of action to disrupt cuticular lipid on both head lice and their eggs, offers a cure rate of around 80% when applied for 8 hours/overnight on two occasions. When formulated in an alcohol free preparation this material was better tolerated and showed only transient side effects in a few participants.

## Supporting Information

Checklist S1 **CONSORT checklist.**
(DOCX)

Figure S1 **Environmental SEM of a head louse treated with a 5% 1,2-octanediol preparation containing zinc nanoparticles.** Figure S1A is the E-SEM image showing the head louse after treatment. There are no obvious indications of any effect of the treatment on the louse surface and no indication of the distribution of the preparation across the cuticle. Figure S1B is the same image using the X-ray mapping spectrograph facility of the E-SEM to show the distribution of zinc nanoparticles suspended in the preparation and deposited across the cuticle surface by the fluid.
(TIF)

Figure S2 **GC-MS chromatogram of cuticle lipid extract from untreated head lice.**
(TIF)

Figure S3 **GC-MS chromatogram of cuticle lipid extract from head lice treated using 5% 1,2-octandiol preparation.**
(TIF)

Protocol S1 **Protocol CTEP02, prepared on behalf of EctoPharma Ltd for Study 2.**
(DOCX)

Protocol S2 **Protocol CTMK12, prepared on behalf of Thornton & Ross Ltd for Study 3.**
(DOCX)

Table S1 **GC-MS recovery of hydrocarbons from lice using eicosane as the reference standard.**
(DOCX)

Table S2 **GC-MS output peak areas covering >1% of the total lipid extract.**
(DOCX)

Text file S1 **Preliminary work investigating the activity of 1,2-octandiol on cuticular lipids of human lice.**
(DOCX)
