# Supplementary figures and images for: 1,2-Octanediol, a Novel Surfactant, for Treating Head Louse Infestation: Identification of Activity, Formulation, and Randomised, Controlled Trials
Source: PLoS One. 2012 Apr 16;7(4):e35419. doi: 10.1371/journal.pone.0035419 (PMC3327678; doi:10.1371/journal.pone.0035419)

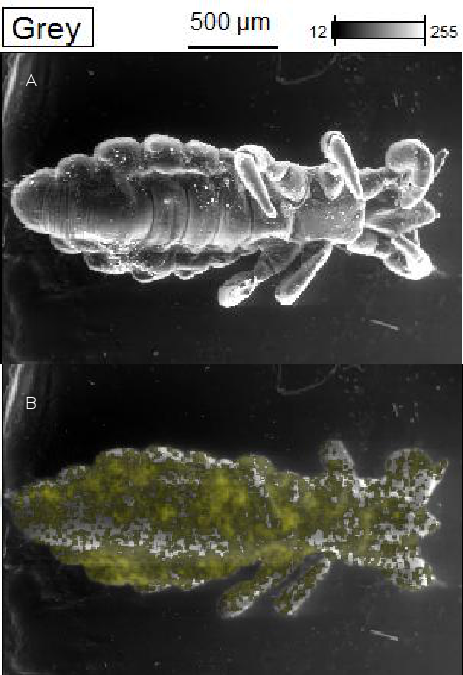

Supplement: Figure S1 — Environmental SEM of a head louse treated with a 5% 1,2-octanediol preparation containing zinc nanoparticles. Figure S1A is the E-SEM image showing the head louse after treatment. There are no obvious indications of any effect of the treatment on the louse surface and no indication of the distribution of the preparation across the cuticle. Figure S1B is the same image using the X-ray mapping spectrograph facility of the E-SEM to show the distribution of zinc nanoparticles suspended in the preparation and deposited across the cuticle surface by the fluid. (TIF) [file pone.0035419.s002.tif]

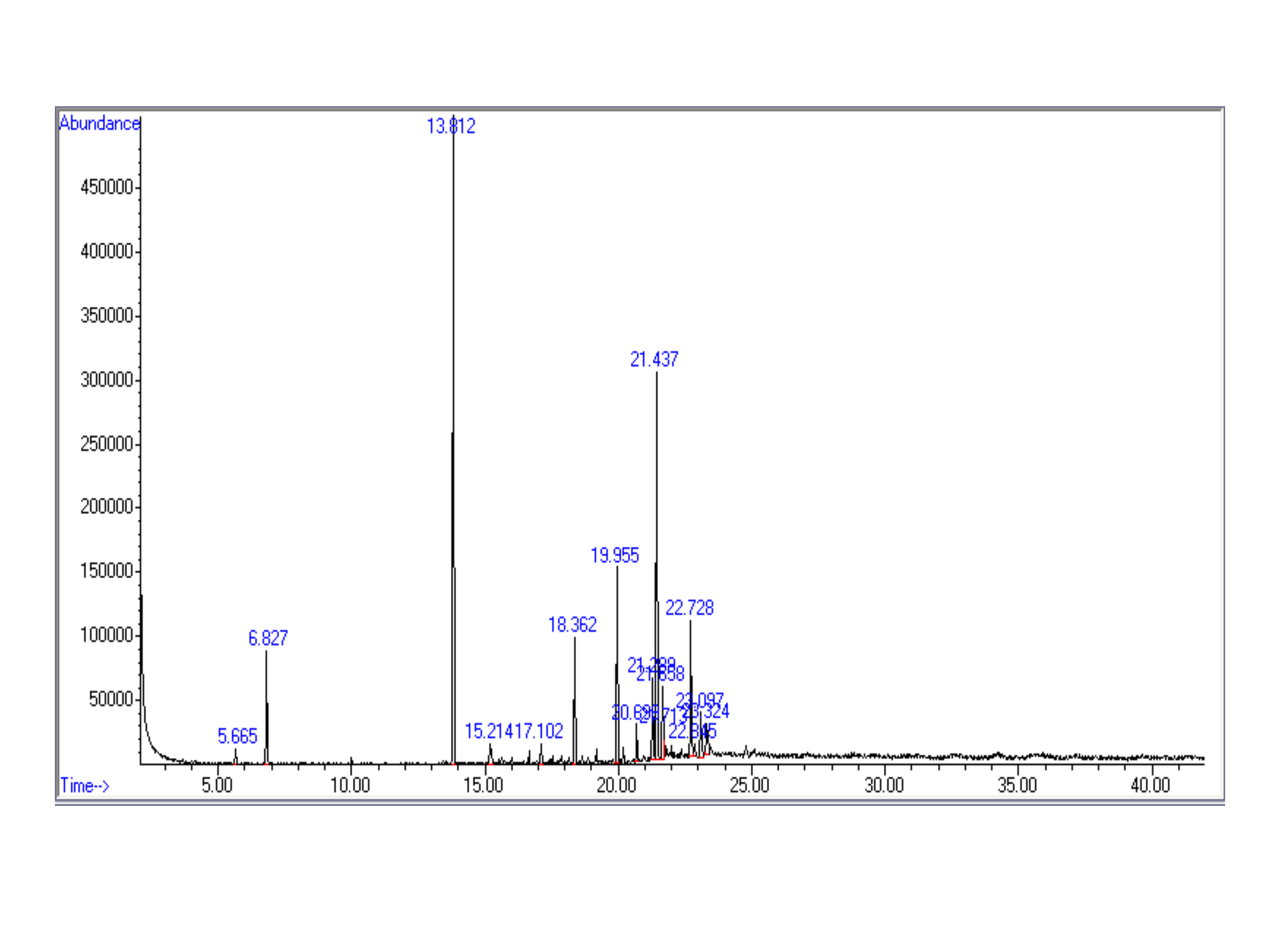

Supplement: Figure S2 — GC-MS chromatogram of cuticle lipid extract from untreated head lice. (TIF) [file pone.0035419.s003.tif]

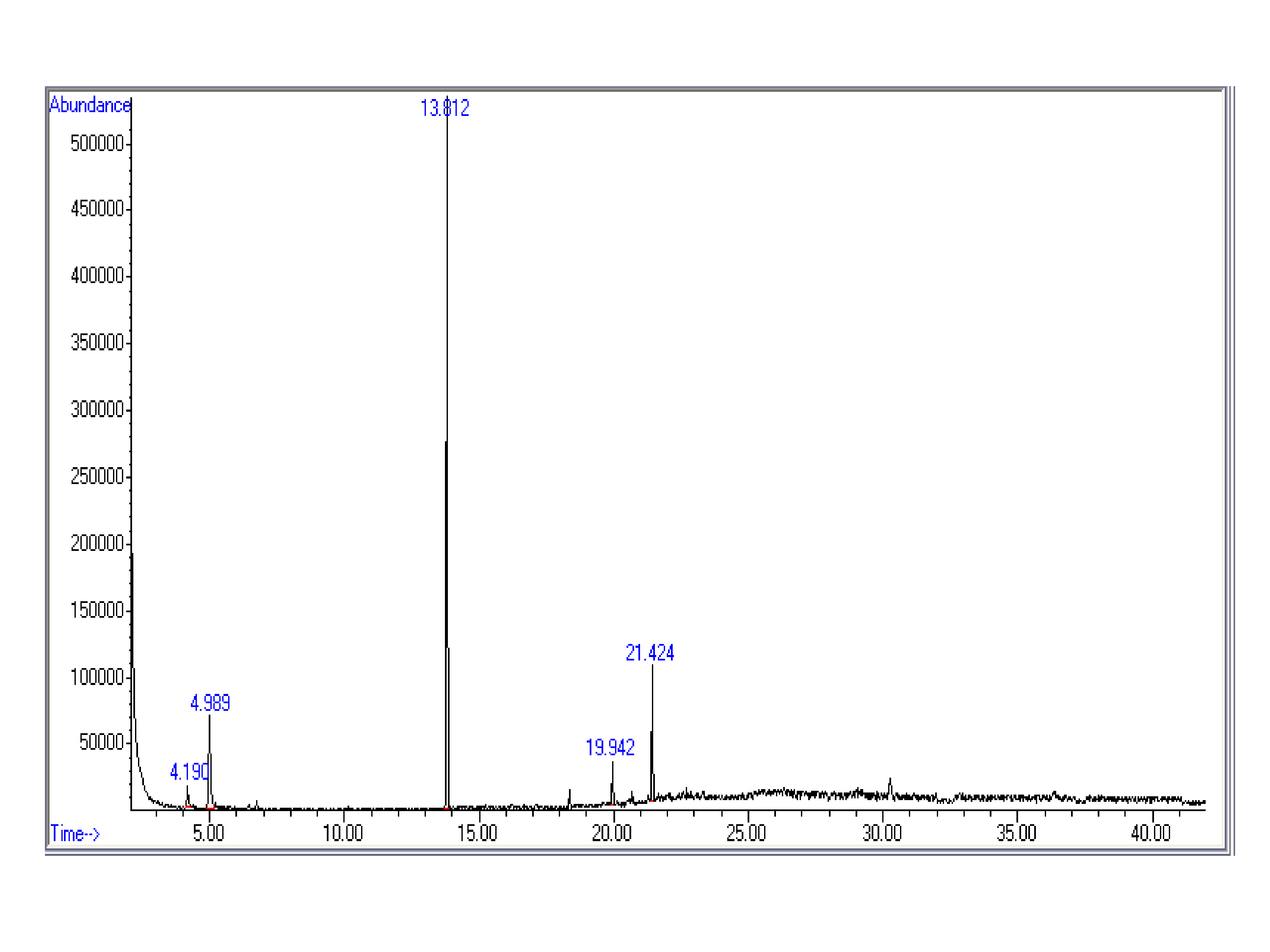

Supplement: Figure S3 — GC-MS chromatogram of cuticle lipid extract from head lice treated using 5% 1,2-octandiol preparation. (TIF) [file pone.0035419.s004.tif]
