# Supplementary material for: 1,2-Octanediol, a Novel Surfactant, for Treating Head Louse Infestation: Identification of Activity, Formulation, and Randomised, Controlled Trials
Source: PLoS One. 2012 Apr 16;7(4):e35419. doi: 10.1371/journal.pone.0035419 (PMC3327678; doi:10.1371/journal.pone.0035419)
Supplement: Protocol S1 — Protocol CTEP02, prepared on behalf of EctoPharma Ltd for Study 2. (DOCX) [file pone.0035419.s005.docx]

**EctoPharma Ltd**

Unit 2, Alpha Centre

Stirling University Innovation Park

Stirling FK9 4NF

Telephone number: 01786 440099

Fax number: 01786 440091

**CLINICAL TRIAL PROTOCOL**

**A Multicentre Phase III trial of 1,2-octanediol at 5%(w/v) (KindaPed™) compared with malathion 0.5% (w/v) (DERBAC-M LIQUID) in the treatment of head lice**

**TRIAL CODE:** **CT EP02**

**CHIEF INVESTIGATOR:** mr Ian Burgess, MEDICAL ENTOMOLOGY CENTRE,

HERTFORDSHIRE, UK

| Draft 1 | 28 March 2007 |
| --- | --- |
| Draft 2 | 13 April 2007 |
| Draft 3 | 18 April 2007 |
| Final Draft | 25 April 2007 |
| Final | 09 May 2007 |
| Final | 21 June 2007 |
| Amendment 1 | 16 July 2007 |
| Amendment 2 | 04 December 2007 |

| **THIS IS A CONFIDENTIAL DOCUMENT**  This document is the property of EctoPharma Limited. The information within it is confidential and is provided to you, for review by you and your staff. The document must be kept in a confidential manner and must be returned to EctoPharma upon request. No part of this document may be reproduced in any form without permission from EctoPharma. By accepting this document, you agree that the information contained therein will not be disclosed to a third party without written authorisation from EctoPharma. |
| --- |

# 1. TITLE PAGE, RESPONSIBLE PERSONNEL, PROTOCOL APPROVAL AND INVESTIGATOR SIGNATURES

## 1.1 TITLE PAGE

**PROTOCOL TITLE:**

A Multicentre Phase III trial of 1,2-octanediol at 5%(w/v) (KindaPed™) compared with malathion 0.5% (w/v) (DERBAC-M LIQUID) in the treatment of head lice

**TRIAL CODE:** CT EP02

**PHASE:** III

**CHIEF INVESTIGATOR:**

Mr Ian Burgess, Director

Medical Entomology Centre, Insect Research and Development Ltd,

Cambridge House, Barrington Road, Shepreth, Royston, Hertfordshire, SG8 6QZ

Telephone number: 01763 263011

Fax number: 01763 263022

E-mail: ian@insectresearch.com

:

**PRINCIPAL INVESTIGATORS:**

Geraldine Matlock, Senior Nurse

Medical Entomology Centre, Insect Research and Development Ltd

Cambridge House, Barrington Road, Shepreth, Royston, Hertfordshire SG8 6QZ

Telephone number: 01763 263011

Fax number: 01763 263022

E-mail: [gerry@insectresearch.com](mailto:gerry@insectresearch.com) / williamm141@aol.com

Ruth Jones, Senior Public Health Nurse

Fronallt, 2, Brondeg Lane, Alltwen, Pontardawe, Swansea, SA8 3AE Telephone number: 07772 780798

Fax number: 01792 862091

E-mail: Ruth1jones@btinternet.com

Katrina Kay, Infection Control Nurse - Ectoparasites

5 Wharfe Grange, Wetherby, Leeds LS22 6SS

Telephone: 07894 583 896 / 07957 378863

Fax number: 0113 2033426

E-mail: Katrina243@aol.com

## 1.2 OTHER RESPONSIBLE PERSONNEL

**SPONSOR/CONTRACT PERSONNEL**

**Medical/Safety Monitoring**

All Serious Adverse Events must be reported by phone and fax directly to:

Carol Markwell, Drug Safety Solutions Ltd., Silver Lining, Londonthorpe, Grantham NG31 9RU

Telephone number: 01476 570819

Fax number: 01476 570819

E-mail: [carol@drugsafetysolutions.co.uk](mailto:carol@drugsafetysolutions.co.uk)

Medical Advisor: Dr Veronica Tebbs

##

**Scientific**

Dr D Magnus Nicolson, EctoPharma Ltd., Unit 2, Alpha Centre

Stirling University Innovation Park, Stirling FK9 4NF

Telephone number: 01786 440 099

Fax number: 01786 440 091

E-mail: [magnusnicolson@ectopharma.com](mailto:magnusnicolson@ectopharma.com)

**Regulatory**

Mrs Fiona MacLeod

13 Liniclate, Isle of Benbecula, Western Isles HS7 5PJ

Telephone number: 01870 602226

Fax number: 0870 134 5343

E-mail: fiona.macleod@theballantynes.co.uk

**Project Management**

Dr Graeme Scott,

PSCR, 7 St Catherine’s Place, Edinburgh, EH9 1NU

Telephone number: 0131 668 2500

Fax number: 0870 120 6210

E-mail: [graemescott@pscr.co.uk](mailto:graemescott@pscr.co.uk)

**Clinical Trial Supplies**

Penn Pharmaceuticals Ltd.,

Units 23-24, Tafarnaubach Industrial Estate, Tafarnaubach, Tredegar, Gwent NP22 3AA

Telephone number: 01495 711222

Fax number: 01495 711225

**Data Management and Statistics**

P.N. Lee Statistics and Computing Ltd

Hamilton House, 17 Cedar Road, Sutton, Surrey SM2 5DA

Telephone number: 0208 6428265

Fax number: 0208 6422135

E-mail: [PeterLee@pnlee.co.uk](mailto:PeterLee@pnlee.co.uk)

**Monitoring Organisations**

Clinical Development & Support Services Limited

Suite 5, Silk House, Park Green, Macclesfield, Cheshire SK11 7QJ

Telephone number: 01625 617 447

Fax number: 0870 0940730

E-mail: [jill.overend@cdssltd.com](mailto:jill.overend@cdssltd.com)

Mrs Mary Mumford

19 Charles Jarvis Court, Cupar, Fife KY15 5EJ

Telephone number: 01334 654282

E-mail: mm.mumford@virgin.net

## 1.3 PROTOCOL APPROVAL

| **PROTOCOL APPROVAL SHEET** |
| --- |

| TRIAL CODE | CT EP02 |
| --- | --- |
| PROTOCOL TITLE | A MULTICENTRE PHASE III TRIAL OF 1,2-OCTANEDIOL AT 5%(W/V) (KINDAPED^TM^) COMPARED WITH MALATHION 0.5% (W/V) (DERBAC-M LIQUID) IN THE TREATMENT OF HEAD LICE |
| AUTHOR(S) | **Author** Graeme Scott, PSCR        (Signature) (Date)    **Statistics Section** Peter N Lee, P.N Lee Statistics and Computing Ltd.  (Signature) (Date) |
| APPROVAL | **Medical Advisor** Dr Veronica Tebbs  (Signature) (Date)  **Sponsor Representative** Dr Magnus Nicolson  (Signature) (Date)  **Chief Investigator** Mr Ian Burgess  (Signature) (Date) |

## 1.4 INVESTIGATOR SIGNATURE SHEET

**Investigator Signature Sheet**

By signing below, I agree to the conditions relating to this trial as set out in this protocol (CT EP02 dated 04 December 2007).

I agree to conduct this clinical trial according to Good Clinical Practice (ICH GCP) and European Regulatory Requirements.

I fully understand that any changes instituted by me without previous discussion with EctoPharma Ltd. or their designated representative constitute a violation of the protocol.

I agree to adhere to the protocol in all circumstances other than where necessary to protect the well-being of the subject.

I will ensure that the trial products supplied by EctoPharma will be used only for administration to subjects included in this trial protocol and for no other purpose.

Principal Investigator’s

Name:___________________________________

Signature:__________________________ Date____________

## 1.5 TABLE OF CONTENTS

[1. TITLE PAGE, RESPONSIBLE PERSONNEL, PROTOCOL APPROVAL AND INVESTIGATOR SIGNATURES.................................................................................2](#_Toc165697325)

[1.1 TITLE PAGE...............................................................................................................2](#_Toc165697326)

[1.2 OTHER RESPONSIBLE PERSONNEL......................................................................3](#_Toc165697331)

[1.3 PROTOCOL APPROVAL...........................................................................................5](#_Toc165697341)

[1.4 INVESTIGATOR SIGNATURE SHEET.....................................................................6](#_Toc165697342)

[1.5 TABLE OF CONTENTS.............................................................................................7](#_Toc165697343)

[1.6 PROTOCOL SYNOPSIS..........................................................................................10](#_Toc165697344)

[1.7 GLOSSARY OF TERMS...........................................................................................14](#_Toc165697345)

[2. INTRODUCTION......................................................................................................15](#_Toc165697348)

[3. OBJECTIVES...........................................................................................................16](#_Toc165697349)

[3.1 PRIMARY OBJECTIVE.............................................................................................16](#_Toc165697350)

[3.2 SECONDARY OBJECTIVES....................................................................................16](#_Toc165697351)

[4. TRIAL DESIGN.........................................................................................................16](#_Toc165697352)

[4.1 OVERVIEW...............................................................................................................16](#_Toc165697353)

[4.2 DISCUSSION OF TRIAL DESIGN............................................................................18](#_Toc165697354)

[4.3 STUDY CENTRES....................................................................................................18](#_Toc165697355)

[4.4 SUBJECT NUMBERS……………………………………............................................18](#_Toc165697356)

[4.5 SUBJECT INCLUSION CRITERIA...........................................................................18](#_Toc165697357)

[4.6 SUBJECT EXCLUSION CRITERIA..........................................................................19](#_Toc165697358)

[4.7 CONDUCT OF THE TRIAL......................................................................................19](#_Toc165697359)

[4.8 SCHEDULE OF TRIAL ACTIVITIES........................................................................24](#_Toc165697360)

[5. TRIAL PRODUCTS..................................................................................................25](#_Toc165697361)

[5.1 INVESTIGATIONAL product...............................................................................25](#_Toc165697362)

[5.2 Comparator........................................................................................................25](#_Toc165697363)

[5.3 DOSAGE AND ADMINISTRATION..........................................................................25](#_Toc165697364)

[5.4 STORAGE................................................................................................................26](#_Toc165697365)

[5.5 PACKAGING AND LABELLING...............................................................................26](#_Toc165697366)

[5.6 ASSIGNMENT OF TREATMENT.............................................................................27](#_Toc165697367)

[5.7 BLINDING.................................................................................................................27](#_Toc165697368)

[5.8 ACCOUNTABILITY OF DRUG AND SUBJECT COMPLIANCE..............................28](#_Toc165697369)

[5.9 CONCOMITANT TREATMENT................................................................................28](#_Toc165697370)

[5.10 WARNINGS AND PRECAUTIONS..........................................................................29](#_Toc165697371)

[5.11 AVAILABILITY OF TREATMENT AFTER THE TRIAL.............................................29](#_Toc165697372)

[6. TRIAL METHODS AND ASSESSMENTS...............................................................29](#_Toc165697373)

[6.1 MEASURES OF OUTCOME....................................................................................29](#_Toc165697374)

[6.1.1 ASSESSMENT OF OUTCOME................................................................................30](#_Toc165697375)

[6.1.2 PRIMARY OUTCOME..............................................................................................31](#_Toc165697376)

[6.1.3 OTHER OUTCOMES................................................................................................31](#_Toc165697377)

[6.2 SAFETY ASSESSMENTS........................................................................................31](#_Toc165697378)

[6.2.1 ADVERSE EVENTS.................................................................................................31](#_Toc165697379)

[6.2.2 SERIOUS ADVERSE EVENTS................................................................................32](#_Toc165697380)

[6.3 PREMATURE DISCONTINUATION.........................................................................33](#_Toc165697381)

[6.4 REPLACEMENT POLICY FOR SUBJECTS WHO DISCONTINUE.........................34](#_Toc165697382)

[7. STATISTICAL ANALYSIS.......................................................................................34](#_Toc165697383)

[7.1 PRIMARY ENDPOINT..............................................................................................34](#_Toc165697384)

[7.2 Secondary endpoints.....................................................................................34](#_Toc165697385)

[7.3 Definition of Populations to be analysed.............................................35](#_Toc165697386)

[7.4 Statistical Methods........................................................................................35](#_Toc165697387)

[7.5 SAMPLE SIZE..........................................................................................................35](#_Toc165697388)

[8. ETHICAL AND REGULATORY CONSIDERATIONS..............................................37](#_Toc165697389)

[8.1 REGULATORY ISSUES...........................................................................................37](#_Toc165697390)

[8.2 INDEMNITY, INSURANCE AND COMPENSATION................................................37](#_Toc165697391)

[8.3 INFORMED CONSENT............................................................................................37](#_Toc165697392)

[8.4 APPROVALS............................................................................................................38](#_Toc165697393)

[8.4.1 ETHICS COMMITTEE APPROVAL..........................................................................38](#_Toc165697394)

[8.4.2 MANAGEMENT APPROVAL....................................................................................38](#_Toc165697395)

[8.5 TRIAL DOCUMENTATION AND TRIAL CONFIDENTIALITY..................................39](#_Toc165697396)

[8.5.1 TRIAL DOCUMENTATION, CRFS AND DOCUMENT KEEPING...........................39](#_Toc165697397)

[8.5.2 DATA MANAGEMENT.............................................................................................41](#_Toc165697398)

[8.5.3 CONFIDENTIALITY OF TRIAL DOCUMENTATION AND SUBJECT RECORDS..41](#_Toc165697399)

[8.5.4 CONFIDENTIALITY OF PROPRIETARY INFORMATION.......................................41](#_Toc165697400)

[8.5.5 DATA PROTECTION................................................................................................42](#_Toc165697401)

[9. PUBLICATION OF DATA........................................................................................42](#_Toc165697402)

[10. ADMINISTRATIVE PROCEDURES.........................................................................42](#_Toc165697403)

[10.1 QUALITY ASSURANCE...........................................................................................42](#_Toc165697404)

[10.2 MONITORING..........................................................................................................42](#_Toc165697405)

[10.3 PROTOCOL AMENDMENTS...................................................................................43](#_Toc165697406)

[10.4 ADMINISTRATIVE AND FINANCIAL AGREEMENT...............................................43](#_Toc165697407)

[10.5 TRIAL SCHEDULE...................................................................................................44](#_Toc165697408)

[10.6 END OF TRIAL.........................................................................................................44](#_Toc165697409)

[10.7 INVESTIGATOR RESPONSIBILITIES.....................................................................44](#_Toc165697410)

[11. REFERENCES...........................................................................................................44](#_Toc165697411)

**LIST OF APPENDICES**

Appendix 1: Sample subject/guardian consent/assent forms

Appendix 2: Schedule of Trial Activities

Appendix 3: Serious Adverse Event Form

## 1.6 PROTOCOL SYNOPSIS

| TITLE | A Multicentre Phase III trial of 1,2-octanediol at 5%(w/v) (KindaPed™) compared with malathion 0.5% (w/v) (DERBAC-M LIQUID) in the treatment of head lice |
| --- | --- |
| TRIAL CODE | CT EP02 |
| PHASE | Phase III |
| OBJECTIVE | The primary objective of the clinical trial is to compare the efficacy of each of two different application regimens (2-hour and 8-hour) of KindaPed™ with the standard regimen of Derbac-M Liquid in eliminating infestation (achieving cure or re-infestation) following two treatment applications.  The secondary objectives of the clinical trial include the comparison of each of two different application regimens of KindaPed™ with the standard regimen of Derbac-M Liquid in respect of the following:   - Efficacy in achieving ‘cure’, based only on the assessments at Days 2 and 6, following the first treatment application. - Efficacy in killing head lice after two applications. - Efficacy in killing louse eggs after two applications. - Safety profile, as determined by the occurrence of adverse events. - Ease of application. - Subject assessment of acceptability. |
| TRIAL DURATION | Subjects will be in the clinical trial for approximately two weeks with treatment applied on Days 0 and 7. |
| TRIAL DESIGN | A phase III, randomised, assessor-blind, multicentre, parallel group trial of two treatment regimens of KindaPed™ and a comparator product (Derbac-M Liquid). |
| NUMBER OF SUBJECTS | A total of 510 subjects (170 in each of three treatment groups) will be recruited. |
| MAIN SELECTION CRITERIA  . | 1. Subjects aged four or over.  2. Subjects who upon examination are confirmed to have live head lice.  3. Subjects who have given written informed consent, or, if the subject is less than 16 years of age, whose parent/guardian has given written informed consent to participate in the study.  4. Subjects who will be available for home visits from research staff over the 15 days of the study. |
| TREATMENTS | KindaPed™ contains:  Active: 1, 2-octanediol at 5% (w/v).  Excipients:Propan-2-ol (IPA)/water 20% (v/v); Sodium Dodecyl Sulphate (SDS) 0.64%  The product will packed in 100 mL low density polyethylene (LDPE) plastic bottles with an applicator nozzle.  Derbac-M Liquid contains:  Active: Malathion at 0.5% (w/v)  Excipients:Methylhydroxybenzoate; Propylhydroxybenzoate; Lanette Wax SX; Potassium Citrate; Citric Acid; Perfume HT 52; Water.  The product is packed in 200 mL clear or amber glass bottles with polyethylene caps. |
| ADMINISTRATION | Treatment will be administered on Days 0 and 7 by research staff, who will not be involved in the assessment of the treated subjects.  **KindaPed™**  KindaPed™ will be applied directly to dry hair. Sufficient product will be applied to thoroughly moisten the hair and scalp. More than one bottle may be used if required (maximum of two bottles).  The period of time for which the product will be left in the hair prior to removal by shampooing will be determined by randomisation. This will be either:   1. At least two hours and no more than two and a half hours before being shampooed, using non-medicated, conditioner-free shampoo which will be supplied for the study, and rinsed off with water. 2. At least eight hours and no more than twelve hours before being shampooed, using non-medicated, conditioner-free shampoo which will be supplied for the study, and rinsed off with water.   Following application of KindaPed™ the hair will be left to dry naturally in a well-ventilated room. Once the treatment has been removed from the hair it can be styled as normal and a hair dryer can be used.  **Derbac-M Liquid**  Derbac-M Liquid will be rubbed into the scalp until all the hair and scalp are thoroughly moistened. The hair will be left to dry naturally in a well-ventilated room. After 12 hours (or the next day if preferred) the hair will be shampooed using a non-medicated, conditioner-free shampoo which will be supplied for the study, and rinsed off with water. Once the treatment has been removed from the hair it can be styled as normal and a hair dryer can be used.  A maximum of one bottle will be used for each treatment application. |
| PROCEDURE | At the recruitment visit (Day 0, Visit 1) verbal consent will be obtained to check for the presence of live head lice by dry combing the hair using a fine-toothed plastic louse detection comb. Lice found during the assessment will not be removed. Other family members who give their verbal permission can also be assessed for the presence of living lice. After the preliminary assessment, subjects can be enrolled to the study, provided they comply with the inclusion/exclusion criteria, and any further questions they may have are fully dealt with. Family members or other members of the household who have lice but are unable or unwilling to join the study will be offered an acceptable, commercially available alternative treatment (Hedrin 4% Lotion).  Subjects (or their parents/guardians if they are aged less than 16 years) will be asked to give written informed consent before participation in the trial. Baseline data will be recorded in the CRF and subjects will be randomised to determine which study treatment they will receive. Treatment will be applied on Day 0 and again on Day 7 (+/- 1 day) by a member of the study team not involved in the subjects’ follow up assessments. The subject will be asked to shampoo the treatment out, using a non-medicated, conditioner-free shampoo. Subjects will be assessed, at their home, for the presence of live head lice on Days 2, 6, 9 and 14 (+/- 1 day at each assessment) by a different assessor from the person who treated them. Any lice found at these assessments will be taped on to a sample form within the subject’s case report form (CRF). Shampoo to be used by the subjects will be supplied.  The study team member who applied the treatment will complete a questionnaire after the first treatment application. At the final visit (Day 14) the subject and/or parent/guardian will complete a questionnaire on the treatment that was applied at Day 0 and Day 7. |
| STATISTICS | For each regimen of KindaPed™, a test will be carried out of the superiority of the KindaPed™ rate of cure or re-infestation over that for Derbac-M Liquid.  Analyses will be conducted based on both the intention to treat (ITT) and the efficacy populations. 97.5% confidence intervals (CI) will be estimated for the primary and some secondary endpoints, 97.5% rather than 95% CI being used to take into account multiple comparisons (of two regimens of KindaPed™ each with Derbac-M Liquid). The distribution of baseline characteristics, safety and ease of use, acceptability and efficacy will be tabulated and analysed. |
| FOLLOW UP | The results of this phase III study will be used to support an application for Marketing Authorisation for KindaPed™. |

## 1.7 GLOSSARY OF TERMS

| ABPI | Association of British Pharmaceutical Industry |
| --- | --- |
| CI | Confidence interval |
| CRF | Case report form |
| CV | Curriculum vitae |
| GCP | Good clinical practice |
| GP | General Practitioner |
| ICH | International Conference on Harmonisation |
| IEC | Independent ethics committee |
| IPA | Isopropyl alcohol |
| ITT | Intention to treat |
| LDPE | Low density polyethylene |
| mL | Millilitre |
| MREC | Multicentre Research Ethics Committee |
| NHS | National Health Service |
| REC | Research Ethics Committee |
| SAE | Serious adverse event |
| SDS | Sodium dodecyl sulphate |
| SSI | Site specific information |
| SUSAR | Suspected, unexpected serious adverse reactions |
| v/v | Volume per unit volume |
| w/v | Weight per unit volume |

# 2. INTRODUCTION

The incidence and prevalence of head lice is no longer monitored in the UK, but recent work commissioned by the Welsh National Assembly showed 8% of children in primary schools had head lice on the day of the check, indicating that almost 1 in 10 of primary school children within the Principality have the infection at any one time (1). Further work carried out in the former North Essex Health Authority gave a prevalence of 2.03% on the day of inspection whilst the results of a questionnaire showed an annual incidence of 37.4% (2).

There is now undisputed evidence from many industrialised countries, including the UK, that strains of head lice have developed resistance to the insecticidal treatments which have been available for many years (3, 4, 5). Added to this is the growing unease from families about the continual use of insecticidal treatments, especially if the claims made by these treatments are perceived as incorrect because family members still have lice after their use.

Alternative “non-insecticidal” measures have been promoted throughout the country but for many families these are not only difficult to perform but do not guarantee success (6,7), and all involve several sessions of combing the hair through with a fine toothed comb, metal or plastic, using conditioner or some other combing agent. Some of these combs have been registered as medical devices but no claims about killing head lice can be made for any combing agents packaged with the combs.

It is advantageous to have a product containing an active ingredient which has a high lice kill-rate with a manageable contact time and which can be easily and effectively applied by the subject and/or their family. The active ingredient in KindaPed™, 1, 2-octanediol, is widely used as an emollient in the skin care and cosmetic industries in products such as facial moisturisers, hair care products and sun care products, as well as in barrier creams used on infants.

A previous clinical trial of KindaPed™, formulated in 50% v/v isopropylalcohol (IPA)/water, showed a high rate of successful cure of head lice using two applications; 65% of subjects said they would use the product again. Nevertheless, there were some issues over the acceptability of the formulation, including odour and a stinging/burning sensation on initial application.

In order to address these issues of subject acceptability, KindaPed™ has been reformulated with a lower alcohol concentration (Section 5.1), and the current study, sponsored by EctoPharma Ltd., is designed to evaluate the efficacy and safety of two application regimens in comparison with a marketed head louse product (Derbac-M Liquid).

# 3. OBJECTIVES

## 3.1 PRIMARY OBJECTIVE

The primary objective of the clinical trial is to compare the efficacy of each of two different application regimens (2-hour and 8-hour) of KindaPed™ with the standard regimen of Derbac-M Liquid in eliminating infestation (achieving cure or re-infestation) following two treatment applications (see algorithm in section 6.1 for definitions of cure and re-infestation).

## 3.2 SECONDARY OBJECTIVES

The secondary objectives of the clinical trial include the comparison of each of two different application regimens of KindaPed™ with the standard regimen of Derbac-M Liquid in respect of the following:

- Efficacy in achieving ‘cure’, based only on the assessments at Days 2 and 6, following the first treatment application.
- Efficacy in killing head lice after two applications.
- Efficacy in killing louse eggs after two applications.
- Safety profile, as determined by the occurrence of adverse events.
- Ease of application.
- Subject assessment of acceptability.

# 4. TRIAL DESIGN

## 4.1 OVERVIEW

This is a phase III, randomised, assessor-blind, multicentre, parallel group trial of two treatment regimens of KindaPed™ and a comparator product (Derbac-M Liquid). It is planned to recruit 510 subjects who meet the selection criteria (Sections 4.5 & 4.6), at three study centres; 170 subjects into each of the three treatment arms.

Potential subjects who respond to advertising will be sent information about the trial. The subject will be invited to make further contact with the study centre by telephone and if, following discussion, it is anticipated that the trial is suitable for the household, a recruitment visit will be arranged at the subjects’ home (Visit 1, Day 0). Subjects must have been in receipt of the study information for at least 24 hours before the recruitment visit takes place. At the recruitment visit verbal consent will be obtained to check for the presence of live head lice by dry combing the hair using a fine-toothed plastic louse detection comb. Lice found during the assessment will not be removed. Other family members who give their verbal permission can also be assessed for the presence of living lice. After the preliminary assessment, subjects can be enrolled to the study, provided they comply with the inclusion/exclusion criteria, and any further questions they may have are fully dealt with. Family members or other members of the household who have lice but are unable or unwilling to join the study will be offered an acceptable, commercially available alternative treatment (Hedrin 4% Lotion).

Subjects (or their parents/guardians if they are aged less than 16 years) will be asked to give written informed consent before participation in the trial (Section 8.3). Baseline data will be recorded in the CRF and subjects will be randomised to determine which study treatment they will receive. Subjects will be treated on Day 0 and again on Day 7 (+/- 1 day) by a member of the study team not involved in the subjects’ follow up assessments. The study product will be applied as described in Section 5.3 and will be removed after the appropriate time by shampooing the hair using a non-medicated, conditioner-free shampoo, which will be supplied for the study.

Derbac-M Liquid will be applied according to instructions supplied with the product (Section 5.3). The subject will be asked to shampoo the treatment out, using a non-medicated, conditioner-free shampoo. Subjects will be assessed, at home, for the presence of live head lice on Days 2, 6, 9 and 14 (+/- 1 day at each assessment) by a different assessor from the person who treated them. Any lice found at these assessments will be taped on to a sample form within the subject’s case report form (CRF) for subsequent characterisation. The shampoo to be used by the subjects will be supplied.

All adverse events will be monitored during the study (Section 6.2.1) and all changes in concomitant illness and medication will be recorded (Section 5.9). A Completion/Withdrawal Form will be completed at the end of the study period.

Each subject will be involved in the trial for approximately two weeks, with treatment being applied on Days 0 and 7.

## 4.2 DISCUSSION OF TRIAL DESIGN

Subjects will be randomised to determine which trial treatment they will receive. A simple randomisation scheme will be used in which each subject has an equal chance of receiving any one of the three treatments.

The difference between the application regimens for the study groups means that it is not possible to ensure that subjects and all trial personnel are blind to treatment. However the assessments for the presence of live head lice and the characterisation of lice found will be carried out by members of the study team who were not involved in handling or administration of the study treatment and are blind to the treatment received. Therefore the clinical trial is single (assessor) blind. This will help to reduce the possibility of bias in the primary outcome measure, which is based on the types of head lice present.

# 4.3 STUDY CENTRES

It is planned that three trial centres in the UK will participate in the clinical trial. Each centre will be expected to recruit at least 100 subjects as part of the overall total of 510. The role of Principal Investigator at each centre will be undertaken by a health professional, who has been trained in the conduct of clinical research, and particularly in the taking of informed consent. At each centre a doctor will have a role in looking after the safety of subjects in the event that they are called to do so.

# 4.4 SUBJECT NUMBERS

It is planned to recruit a total of 510 subjects with 170 in each of three treatment arms.

The trial has been designed bearing in mind that each application regimen of KindaPed™ will separately be compared with Derbac-M Liquid. Details of the sample size calculation are provided in Section 7.5. One hundred and sixty-three (163) subjects per group provides 90% power to detect superiority of KindaPed™ using a 1-tailed alpha of 2.5%. To allow for early withdrawal, or other reasons for subjects being non-evaluable, a total of 170 per arm will be recruited.

## 4.5 SUBJECT INCLUSION CRITERIA

To be eligible for the trial all of the following criteria must be fulfilled:

1. Subjects aged four or over.

2. Subjects who upon examination are confirmed to have live head lice.

3. Subjects who have given written informed consent, or, if the subject is less than 16 years of age, whose parent/guardian has given written informed consent to participate in the study.

4. Subjects who will be available for home visits from research staff over the 15 days of the study.

## 4.6 SUBJECT EXCLUSION CRITERIA

The subject is not eligible to participate in the trial in any of the following circumstances.

1. Subjects with a known sensitivity to any of the ingredients in the products.

2. Subjects with a secondary bacterial infection of the scalp (*e.g.* impetigo) or who have a long-term scalp condition (*e.g.* psoriasis of the scalp).

3. Subjects currently on prescribed medication for the prophylaxis or treatment of asthma. (‘Currently’ will be interpreted as meaning that the subject has used asthma medication within the previous four months.). .

4. Subjects who have been treated with other head lice products within the last two weeks. There must have been a 14-day gap since treatment for head lice was last used before the subject can be accepted into this trial.

5. Subjects who have bleached hair, or hair that has been colour treated or permanently waved within the last four weeks (wash in/wash out colours are acceptable).

6. Subjects who have been treated with the antibiotics Co-Trimoxazole, Septrin or Trimethoprim within the previous four weeks, or who are currently taking such a course.

7. Pregnant or nursing mothers.

8. Subjects who have participated in another clinical trial within one month prior to entry to this study.

9. Subjects who have already participated in this clinical study or any clinical study of KindaPed™.

## 4.7 CONDUCT OF THE TRIAL

**Pre-recruitment**

Subjects will be invited to join the study by advertisements in local newspapers and radio. General Practitioners, Pharmacies, School Nurses and Health Visitors, as well as the Primary Care Trusts and the Health Protection Agency, will be provided with information about the clinical trial. A contact telephone number will be given and potential study recruits will be asked to telephone for more information if they would like to take part in the trial.

A member of the study team will conduct a brief telephone interview to establish whether the subject is suitable for entry into the trial. If it appears that a subject is suitable for entry a detailed Subject Information Sheet, giving all the information about the trial, will be posted to them. This will include a children’s section explaining what will happen if they enter the trial.

After reading the information sheet, prospective subjects wishing to take part will be invited to telephone the centre to arrange a recruitment visit at their home. Subjects must have been in receipt of the information sheet for at least 24 hours before the recruitment visit can take place.

**Visit 1 (Day 0)**

**Recruitment/Screening/Treatment**

At the first visit (Visit 1, Day 0), verbal consent will be obtained to check for the presence of live head lice. This check will be performed by dry combing the hair using a fine-toothed plastic louse detection comb. Lice found during the assessment will not be removed. Details of how many people share the place of residence with the subject, the number of people assessed and found to have lice, anyone unable to join the trial and the number of people enrolled into the study from the household will also be recorded. Subjects (or their parents/guardians if they are aged less than 16 years) will be asked to give written informed consent before participation in the trial (Section 8.3).

Subjects who are enrolled into the study will have baseline information recorded in the CRF, including hair characteristics, previous treatment for lice infection, infestation level, current medication. The team member applying the treatment will randomise the subject to determine which treatment they will receive by selecting the next available treatment kit from her current block of treatments. The randomised treatment will be applied as described in Section 5.3. The subject’s trial number and initials will be added to the label of the bottle used. Any product remaining after the treatment will be retained and returned to the study centre so that the weight can be recorded and the quantity of product used calculated. A log will be kept of which numbered bottles each subject received.

Subjects will be reminded that a second treatment will be applied on Day 7 and a card giving the dates and times of the appointments for the second treatment and for the assessments will be left with the household.

The following information will be recorded in the CRF:

1. Declaration of Receipt of Informed Consent.

Confirmation that informed consent and assent (where relevant) has been obtained, that a copy of the consent has been given to the subject and/or parent/guardian and that the original will be retained.

1. Identification.

Subject's initials, gender, age, date of birth.

1. Hair Characteristics.

Characteristics of the subject’s hair:

- 1. Length: closely cropped, above ears, ears to shoulders, below shoulders.
  2. Thickness: fine, medium, thick.
  3. Degree of curl: straight, wavy, slight curl, tight curl.
  4. Type: dry, normal, greasy.

1. Head Lice Details.

When the subject was last treated for head lice (an exact date if treated within the previous four weeks), the treatment that was used and the outcome (success or failure). The severity of the current louse infestation will be estimated using the following scale:

a) None^[[1]](#footnote-1)^

b) Light infestation: lice only found after 5-6 combs of the hair.

c) Moderate infestation: single louse found on the first comb of the hair.

d) Heavy infestation: more than one louse found on the first comb of the hair.

The severity of the infestation will be re-assessed and recorded in the CRF at the end of the treatment period.

1. Medication Current at Entry.

Any medication being taken; the date the medication started; the total dose per day; and the reason for the medication.

1. Medical History.

Medical history and any current illnesses will be recorded.

1. Inclusion/Exclusion Criteria.

Confirmation that the subject meets the inclusion/exclusion criteria for entry into the study.

Investigator Questionnaire:

The study team member who applied the treatment will complete a questionnaire after the first treatment application. This will include questions on how easy the product was to apply to the hair and scalp, how easy it was to work the product in, how long it took to apply the product and how satisfied they were that the product saturated the hair and scalp.

**Visit 2 (Day 2), Visit 3 (Day 6)**

Follow-up assessments will take place on Days 2 and 6 (+/- 1 day at each assessment). The subject’s hair will be combed with a head louse detection comb, and any lice found will be taped (with clear adhesive tape) on to a tear-out page in the subject’s CRF. The study team members conducting the assessments on Days 2 and 6 will be different from the team member who applied the treatment at Day 0. Changes in medication and any adverse events occurring since the previous visit will be recorded.

**Visit 4 (Day 7)**

On Day 7 a second application of the randomised treatment will be made, where possible, by the same team member who applied treatment at Day 0. Changes in medication and any adverse events occurring since the previous visit will be recorded.

**Visit 5 (Day 9), Visit 6 (Day 14)**

Follow-up assessments will take place on Days 9 and 14 (+/- 1 day at each assessment). The subject’s hair will be combed with a head louse detection comb, and any lice found will be taped (with clear adhesive tape) into the subject’s CRF. The study team members conducting the assessments on Days 9 and 14 will be different from the team member who applied the treatment at Days 0 and 7. Changes in medication and any adverse events occurring since the previous visit will be recorded.

At the final assessment on Day 14 the severity of louse infection will be assessed and the subject and/or parent/guardian will complete a questionnaire on the treatment that was applied at Day 0 and Day 7. This will include questions on how the subject’s scalp felt, how the hair felt after treatment, how the product smelled, how easily the product washed out and whether the subject and/or parent/guardian would use the product again.

The Completion/Withdrawal Form will be completed after the CRF has been returned to the study centre.

**Characterisation of Lice**

Any lice found and removed on Days 2, 6, 9 or 14 will be examined under the microscope to establish the sex and/or stage of development. The presence of small lice (nymphs) will be evidence that not all of the eggs were killed by the first treatment. Continued monitoring will enable the investigation to determine whether the presence of any lice is due to surviving lice or surviving eggs from which nymphs emerge. The criteria for re-infection, after the initial infestation has been cleared, are defined in Section 6.1.

The personnel involved in the characterisation of the lice will be blind to the treatment received by the subject.

The schedule of trial activities is shown in Section 4.8 and details of the assessment parameters are given in Section 6.

## 4.8 SCHEDULE OF TRIAL ACTIVITIES

| **Procedure** | **Visit 1**  **Day 0** | **Visit 2**  **Day 2** (±1) | **Visit 3**  **Day 6** (±1) | **Visit 4**  **Day 7** (±1) | **Visit 5**  **Day 9** (±1) | **Visit 6**  **Day 14** (±1) |
| --- | --- | --- | --- | --- | --- | --- |
| Verbal consent | **X**^1^ |  |  |  |  |  |
| Eligibility criteria | **X** |  |  |  |  |  |
| Presence/absence of lice | **X** | **X** | **X** |  | **X** | **X** |
| Collection of lice for characterisation |  | **X** | **X** |  | **X** | **X** |
| Written informed consent | **X**^2^ |  |  |  |  |  |
| Randomisation | **X** |  |  |  |  |  |
| Hair characteristics | **X** |  |  |  |  |  |
| Previous treatment for lice infection | **X** |  |  |  |  |  |
| Infestation level | **X** |  |  |  |  | **X** |
| Current medication | **X** |  |  |  |  |  |
| Application of study product | **X** |  |  | **X** |  |  |
| Investigator questionnaire | **X^3^** |  |  |  |  |  |
| Changes in medication |  | **X** | **X** | **X** | **X** | **X** |
| Adverse events | **X** | **X** | **X** | **X** | **X** | **X** |
| Subject questionnaire |  |  |  |  |  | **X** |

^1^ Verbal consent required before any study procedures were undertaken

^2^  Written informed consent to be obtained after screening for head lice and checking of inclusion/exclusion criteria before any other study procedures were carried out

^3^ Following application of study product

#

# 5. TRIAL PRODUCTS

## 5.1 INVESTIGATIONAL product

KindaPed contains:

Active: 1, 2--octanediol at 5% (w/v).

Excipients: Propan-2-ol (IPA)/water 20% (v/v);

Sodium Dodecyl Sulphate (SDS) 0.64%

The product will packed in 100 mL low density polyethylene (LDPE) plastic bottles with an applicator nozzle.

## 5.2 Comparator

Derbac-M Liquid contains:

Active: Malathion at 0.5% (w/v)

Excipients: Methylhydroxybenzoate; Propylhydroxybenzoate; Lanette Wax SX; Potassium Citrate; Citric Acid; Perfume HT 52; Water.

The product is packed in 200 mL clear or amber glass bottles with polyethylene caps.

##

## 5.3 DOSAGE AND ADMINISTRATION

All products supplied in connection with this clinical trial must be used only for this and no other purpose.

Treatment will be administered on Days 0 and 7 by research staff, who will not be involved in the assessment of the treated subjects or characterisation of lice found.

**KindaPed™**

KindaPed™ will be applied directly to dry hair. Sufficient product will be applied to thoroughly moisten the hair and scalp. More than one bottle may be used if required (maximum of two bottles).

The period of time for which the product will be left in the hair prior to removal by shampooing will be determined by randomisation. This will be either:

1. At least two hours and no more than two and a half hours, before being shampooed using non-medicated, conditioner-free shampoo which will be supplied for the study, and rinsed off with water.

2. At least eight hours and no more than twelve hours, before being shampooed using non-medicated, conditioner-free shampoo which will be supplied for the study, and rinsed off with water.

Following application of KindaPed™ the hair will be left to dry naturally in a well-ventilated room. The treatment should be applied at least one hour before the subject goes to bed (if treatment is to be left on overnight) so that the hair has sufficient time to dry naturally after treatment. Once the treatment has been removed from the hair it can be styled as normal and a hair dryer can be used.

**Derbac-M Liquid**

Derbac-M Liquid will be rubbed into the scalp until all the hair and scalp are thoroughly moistened. The hair will be left to dry naturally in a well-ventilated room. The treatment should be applied at least one hour before the subject goes to bed (if treatment is to be left on overnight) so that the hair has sufficient time to dry naturally after treatment. After 12 hours (or the next day if preferred) the hair will be shampooed using a non-medicated, conditioner-free shampoo which will be supplied for the study, and rinsed off with water. Once the treatment has been removed from the hair it can be styled as normal and a hair dryer can be used.

A maximum of one bottle will be used for each treatment application.

## 5.4 STORAGE

All supplies used in the study must be maintained securely, at room temperature, under the direct responsibility of the Principal Investigator or under that delegated by the investigator.

## 5.5 PACKAGING AND LABELLING

**KindaPed™**

The product will packed in 100 mL LDPE plastic bottles with an applicator nozzle. A clinical trial label will be affixed identifying the individual bottle number and a blank section will be provided for completion of subject number and initials. The product label will contain appropriate clinical trial wording, including ‘For Clinical Trial Use Only’. Labelling will comply with applicable regulatory guidelines.

**Derbac-M Liquid**

The product is packed in 200 mL clear or amber glass bottles with polyethylene caps. A clinical trial label will be affixed identifying the individual bottle number and a blank section will be provided for completion of subject number and initials. The product label will contain appropriate clinical trial wording, including ‘For Clinical Trial Use Only’. Labelling will comply with applicable regulatory guidelines.

**Shampoo**

A non-medicated, conditioner-free shampoo will be supplied in 30 mL plastic universal containers, which will be labelled with clinical trial labels.

## 5.6 ASSIGNMENT OF TREATMENT

Subjects will be randomised at Visit 0 to one of three treatment groups (KindaPed™ 2-hour application, KindaPed™ 8-hour application, Derbac-M Liquid).

The randomisation will be prepared in blocks of 12 and the treatment kits numbered according to the randomisation scheme.

Each study site will be provided with stocks of pre-numbered treatment kits and each investigator / designated team member will initially be assigned a block of 12 treatment kits from this stock. They will randomise a subject by allocating them to the next available treatment kit number from their block. Each investigator / designated team member will use a complete block before allocating treatments from a new block

## 5.7 BLINDING

Due to the different application regimens of the study treatments, it is not possible to blind either the subject or the member of the study team applying the product to the treatment received. Although no attempt will be made to package the study products in a blinded fashion, the number of the treatment pack will not be an indication of which treatment it contains. Therefore knowledge of the treatment number alone will not unblind the study. The member of the study team performing the assessment for the presence of live head lice at the study visits will be blind to the treatment received by the subject, as will be the person characterising any lice found, and therefore the trial is single (assessor) blind. Assessors will remind subjects to refrain from remarking on the treatment received, other than to respond to the questions asked. Access to treatment logs will be restricted to site staff involved in the treatment of subjects.

Emergency code break envelopes will be produced to provide details of which treatment each subject was allocated. Sealed code break envelopes will be held by the investigator and at Drug Safety Solutions, which is responsible for safety monitoring. An envelope may only be opened in the case of a serious adverse event and only when knowledge of the study treatment is essential to the subsequent management of the subject. Where a code break occurs, the Investigator must provide a written record of the circumstances surrounding the event but should take care not to record details of the unblinded treatment on any trial documentation. Such details should be disclosed only to those persons who have responsibility for the immediate management of the patient. EctoPharma must be notified as soon as possible.

At the end of the trial, all code break envelopes must be returned, along with the drug dispensing records to the trial monitor. The envelopes will be checked to ensure that the seals have not been broken, unless in the previously described circumstances.

Every attempt should be made to ensure that all persons directly involved in the trial are blind to the randomisation code, other than study team members who are involved in treatment application.

## 5.8 ACCOUNTABILITY OF DRUG AND SUBJECT COMPLIANCE

All supplies will be dispensed in accordance with the Investigator's prescription and it is the Investigator's responsibility to ensure that an accurate record of supplies issued and returned is maintained. All supplies will be used only for subjects during their participation in the clinical trial. The bottles of test and comparator products used in the trial will be numbered and weighed on calibrated scales before use.

Any product remaining after the treatment will be retained and returned to the study centre so that the weight can be recorded and the quantity of product used can be calculated. The weights of the bottles before and after use will be recorded on a Treatment Allocation Log. This will be managed in such a way so as to ensure that staff involved in study assessments are not unblinded to the treatment received by individual subjects. All used, partly used and unused bottles of study products will be collected by the trial monitor and returned to Penn Pharmaceuticals at the end of the trial for destruction.

5.9 CONCOMITANT TREATMENT

The subject should not use any other form of pediculicide treatment whilst taking part in the clinical trial; this includes the use of a fine-toothed comb to remove head lice. If the use of such treatment occurs, the subject will be withdrawn from the study.

Other medication can be prescribed in the normal way, although subjects requiring Co-Trimoxazole, Septrin or Trimethoprim should be withdrawn from the study.

All concomitant medicines should be listed in the CRF and any changes to such medicines during the course of the study recorded.

## 5.10 WARNINGS AND PRECAUTIONS

It is advisable that protective plastic or rubber gloves be worn by the nurse applying the study product for the clinical trial.

Allow the hair to dry in a well-ventilated room and do not cover the head before the lotion has dried completely.

If there is persistent irritation, seek medical advice immediately.

Avoid naked flames or lighted objects.

## 5.11 AVAILABILITY OF TREATMENT AFTER THE TRIAL

It is anticipated that, if this trial is successful, marketing authorisation applications will be made. Until such time as authorisations are granted, KindaPed™ will not be available for use outside of clinical trials. Family members, or other members of the household who have lice but are unable or unwilling to participate in the clinical trial, will be offered an acceptable, commercially available alternative treatment (Hedrin 4% Lotion).

Subjects who still have live lice at the end of the study will be offered an acceptable, commercially available alternative treatment (Hedrin 4% Lotion).

# 6. TRIAL METHODS AND ASSESSMENTS

## 6.1 MEASURES OF OUTCOME

The primary measure will be the number of subjects satisfying the criteria for cure or re-infestation as defined by the treatment outcome algorithm below. These subjects will all be regarded as having been cured of the infestation evident at Day 0.

### 6.1.1 ASSESSMENT OF OUTCOME

Based on the assessments at Days 2, 6, 9 and 14 the subjects will initially be divided into five groups:

**1. Cure**

No lice present on Day 9 and Day 14.

These subjects will all be cured of the infestation evident at Day 0.

**2. Re-infestation**

Occurs following a successful treatment but the subject is re-infested from a contact during the post treatment assessment period. This is identified by discovery of limited* numbers of new lice invading the head.

The following must all apply:

- Lice must be present at Day 9 and/or at Day 14.
- Adult lice and/or stage 3 nymphs must not be present at Day 2 or Day 6.
- Stage 1 or 2 nymphs must not be present at Day 9 or Day 14.
- If stage 3 nymphs are present at Day 9 there must be no stage 1 or 2 nymphs present at Day 6.
- Stage 3 nymphs may be present at Day 9 or Day 14 although they must not exceed 2* in number.
- Adult lice should not exceed 2* at either Day 9 or Day 14.

* The number 2 is an interpretation of the limits of “limited”.

**3. Ovicidal Failure Only**

Ovicidal failure is evidenced by the presence of stage 1 nymphs within 2 days of the second treatment administered on Day 7

Thus specific lice must be present at Day 9 and Day 14 as below:

- At Day 9 there must be no adult lice and no stage 2 or stage 3 nymphs.
- At Day 14 there must be no adult lice or stage 3 nymphs.

This is equivalent to requiring stage 1 nymphs at Day 9 and/or stage 1 and/or stage 2 nymphs at Day 14.

**4. Licidal Failure Only**

All other outcomes are licidal failures. Those in group 4 have no stage 1 nymphs at Day 9 and no stage 1 or stage 2 nymphs at Day 14, so do not satisfy the condition for ovicidal failure.

1. **Licidal and Ovicidal Failure**

Any subject not falling into groups 1-4.

The five groups defined above are used for assessing outcome from both treatments. One of the secondary endpoints assesses the outcome of the first treatment, based only on the assessments made at Days 2 and 6. Here subjects are simply defined as being cured or not, with “cure” defined as no lice present at either Day 2 or Day 6.

### 6.1.2 PRIMARY OUTCOME

The primary measure will be the number of subjects satisfying the criteria for cure or re-infestation, groups 1 and 2 in the treatment outcome algorithm defined above in section 6.1.1. These subjects will all be regarded as having been cured of the infestation evident at Day 0.

### 6.1.3 OTHER OUTCOMES

The primary outcome measure is concerned with success in killing the lice and eggs present at the initial infestation. The outcome assessment algorithm can also be used to determine success in killing lice (groups 1, 2 and 3 combined) and success in killing eggs (groups 1, 2 and 4 combined).

## 6.2 SAFETY ASSESSMENTS

### 6.2.1 ADVERSE EVENTS

An adverse event is any illness, sign or symptom, whether or not it is considered to be related to the study product. Details of adverse events will be collected at every trial visit and will include:

- Serious (Yes, No).
- Onset date.
- Severity (mild, moderate, severe).
- Action taken (none, study product stopped, concomitant medication required, outpatient referral).
- Relationship to study product (none, unlikely, possible, probable).
- Outcome (resolved, resolved with sequelae, persists, death).
- Resolution date (if applicable).

All adverse events will be followed until resolution or stabilisation.

Adverse events must be recorded in the CRF on the corresponding assessment form and on an Adverse Event Form.

### 6.2.2 SERIOUS ADVERSE EVENTS

Serious adverse events (SAE) are defined according to the International Conference on Harmonisation (ICH) Guidelines as any untoward medical occurrence that at any dose:

- Results in death.
- Is life-threatening, requires in-patient hospitalisation or prolongs existing hospitalisation.
- Results in persistent or significant disability/incapacity.
- Is a congenital anomaly/birth defect.

Any adverse event that requires medical or surgical intervention to prevent one of the outcomes listed above is also classified as an SAE.

If the adverse event is serious, it shall be reported immediately, by telephone / facsimile to the Pharmacovigilance Consultant. A full written report must be sent, by facsimile, within 24 hours of notification of the event.

SAEs must be reported to:

| Carol Markwell  Parmacovigilance Consultant  Drug Safety Solutions Ltd.,  Silver Lining, Londonthorpe,  Grantham NG31 9RU  Tel: 01476 570819  Fax: 01476 570819 |
| --- |
| Mobile: 07733 300 742 |

The Investigator will assess whether the event is causally related to the study product, and a research physician will also make an assessment of causality on behalf of the sponsor. If causality, by either assessment, is deemed to be probable, possible or unknown, and the SAE is unexpected (not previously documented in the Investigator Brochure), then the event will be reported as a suspected, unexpected serious adverse reaction (SUSAR). The sponsor will notify the independent ethics committee (IEC) that approved the protocol of any SUSARs that are fatal or life-threatening within seven days, with any further relevant information being submitted within an additional eight days. All other SUSARs will be notified within 15 days. The sponsor will also notify the appropriate regulatory agency and all participating investigators of all SUSARs occurring during the trial. The individual investigators are responsible for notifying their IEC of SAEs, according to current local requirements.

All SAEs must be reported in the CRF on the corresponding assessment form and on an Adverse Event Form, as well as being reported separately on the SAE form (Appendix 3).

## 6.3 PREMATURE DISCONTINUATION

Subjects may be withdrawn from the study at any time for whatever reason. Details of the reason will be recorded according to the following convention:

**Adverse Event**

The subject is withdrawn from the study by the Investigator because of an adverse event, whether or not the Investigator believes it to be serious or caused by the study medication, and provided that the Investigator considers it is in the subject's best interest to be withdrawn. There must be a corresponding entry on the assessment form and on the Adverse Event Form.

**Non-compliance**

The subject is withdrawn because of failure to comply with the treatment regimen, or comply with the investigations as required, but is still accessible to the Investigator.

**Drop Out**

The subject withdraws consent to continue in the study, but the Investigator would otherwise consider it appropriate for him/her to continue.

**Lost to Follow-up**

The subject, without explanation, fails to keep appointments as scheduled for study assessments and is not seen again despite the Investigator's effort (letter, telephone, home visit *etc.*) to re-establish contact.

**Death**

All deaths will be treated as SAEs and EctoPharma must be informed within 24 hours of the clinical trial team being notified. All associated documentation must be completed within three working days. Full details will be required including a post-mortem examination if possible.

**Lack of Efficacy**

The subject elects to withdraw, because the study medication is not adequately effective.

By agreement between EctoPharma and the Chief Investigator, the study may be terminated at any time if the recruitment rate is such that the required number of subjects will not be recruited within the specified time, if the products being used are deemed to be failing unacceptably or if any safety concerns arise. In the event of early termination the relevant ethics committee and regulatory authority will be notified within the statutory 15 days.

Should the decision be made by EctoPharma to terminate the study at any time, such decision will be communicated to the Chief Investigator in writing, and appropriate arrangements will be agreed upon and specified in writing. Conversely, should the Investigator decide to withdraw from execution of the study he/she will communicate immediately such decision in writing to EctoPharma.

## 6.4 REPLACEMENT POLICY FOR SUBJECTS WHO DISCONTINUE

Subjects who withdraw will not be replaced. The number of subjects recruited is sufficient to allow for 10% of subjects withdrawing or being otherwise non-evaluable.

# 7. STATISTICAL ANALYSIS

## 7.1 PRIMARY ENDPOINT

The primary endpoint is the proportion of subjects satisfying the criteria for cure or re-infestation as defined by the treatment outcome algorithm (Section 6.1). These subjects will all have been cured of the infestation evident at Day 0.

## 7.2 Secondary endpoints

Secondary measures will be:

1. The proportion of subjects satisfying the criteria for cure based only on the assessments at days 2 and 6 following the first treatment application. (see Section 6.1).

2. The proportion of subjects satisfying the criteria for killing lice following two treatment applications (see Section 6.1).

3. The proportion of subjects satisfying the criteria for killing eggs following two treatment applications (see Section 6.1).

4. The safety of the study products, as determined by the occurrence of adverse events.

5. Ease of application of the study products as assessed by an investigator questionnaire.

6. Subject assessment of acceptability.

## 7.3 Definition of Populations to be analysed

**Efficacy Population**

This will include all randomised subjects who are treated according to the study protocol.

**Intention to treat (ITT) Population**

This will include all randomised subjects who are treated at least once. Premature terminations due to treatment failure, adverse events *etc*., will be included.

## 7.4 Statistical Methods

**Analytical Methods**

An independent statistician (P. N. Lee Statistics and Computing Ltd) will undertake the statistical analysis on behalf of EctoPharma.

For each regimen of KindaPed™, a test will be carried out of the superiority of the KindaPed™ rate of cure or re-infestation over that for Derbac-M Liquid.

Analyses will be conducted based on both the ITT and the efficacy populations. 97.5% CI will be estimated for the primary and some secondary endpoints, 97.5% rather than 95% CI being used to take into account multiple comparisons (of two regimens of KindaPed™ each with Derbac-M Liquid). The distribution of baseline characteristics, safety and ease of use, acceptability and efficacy will be tabulated and analysed, the details to be described in a Statistical Analysis Plan.

## 7.5 SAMPLE SIZE

It is planned to recruit a total of 510 subjects, 170 in each of three treatment arms.

The trial has been designed bearing in mind that each application regimen of KindaPed™ will separately be compared with Derbac-M Liquid. It is believed that the efficacy of Derbac-M Liquid when applied by professionals is 35% and that the efficacy of each application regimen of KindaPed™ might exceed this by at least 25%. One hundred and twenty-six (126) subjects per group provides 95% power to detect superiority of KindaPed™ using a 1-tailed alpha of 2.5%. However, as this is the only trial being conducted for planned registration of KindaPed™, the possibility of a poorer outcome was also considered. The outcomes considered were KindaPed™ having superior efficacy of only 20% (55% vs 35% and 60% vs 40%). These scenarios require 162 or 163 subjects per group to provide 90% power to detect superiority of KindaPed™ on an otherwise similar basis. To allow for early withdrawal or other reasons for subjects being unevaluable a total of 170 per arm will be recruited

Recruitment will continue until the number of subjects is at least 510.

# 8. ETHICAL AND REGULATORY CONSIDERATIONS

## 8.1 REGULATORY ISSUES

The investigators are responsible for ensuring that the trial is conducted at their sites in accordance with the principles of the Declaration of Helsinki (Edinburgh, 2000) and, where applicable, in compliance with the NHS Research Governance Framework, ICH Good Clinical Practice (GCP) (CPMP/ICH/135/95, July 1996), the European Directive on Clinical Trials (2001/20/EC, 04 April 2001 and subsequent amendments) and the European Directive on Good Clinical Practice (2005/28/EC).

The sponsor will be responsible for ensuring that the approval is obtained from the local regulatory authority prior to the start of the trial. The relevant documents will be provided to the Investigator. The sponsor will forward any protocol amendments to the regulatory authority and will ensure that SAEs are reported, and progress reports and details of any serious protocol violations are provided as required. The regulatory authority will be informed should the trial be terminated early.

## 8.2 INDEMNITY, INSURANCE AND COMPENSATION

EctoPharma confirms that this clinical trial is protected by insurance cover which provides an indemnity to the investigators and their co-workers, subject to the Policy terms, conditions and limitations, and provided always that the clinical trial is conducted and the data are reported according to the standards determined by the protocol.

The health professionals engaged in the work of the trial at the study sites are covered by Professional Indemnity Insurance.

EctoPharma maintains in force a ‘no fault’ compensation insurance indemnity in accordance with the current version of the Association of the British Pharmaceutical Industry (ABPI) Guidelines on Clinical Trials: ‘Compensation for Medicine Induced Injury’. In the event that the compensation on a ‘no fault basis’ is unacceptable to the claimant, the Policy will, subject to its terms, conditions and limitations, respond to an action for legal liability arising out of this clinical study.

## 8.3 INFORMED CONSENT

Subjects and/or parents/guardians will be asked if they understand the requirements of the clinical trial and whether they have any further questions concerning it. Provided they still wish to take part in the trial and meet the inclusion/exclusion criteria for entry, the subject or their parent/guardian (when the subject is under the age of 16) will read and sign the consent form (Appendix 1). The Investigator carrying out the enrolment procedure is also required to countersign the consent form. A separate assent form will be available for those below the age of 16 who are able to sign their name and a verbal assent form will be available for children not capable of signing their name. The Investigator and the parent/guardian will countersign the assent forms.

A copy of the signed consent form and of any assent forms will be returned to the subject or their parent/guardian. The original copy will be kept under separate cover at the study site.

The subject’s General Practitioner (GP) will be informed by letter from the Principal Investigator that their subject is participating in the clinical trial, provided consent has been obtained for this to be done. Lack of consent to inform the GP will not prevent subject participating.

## 8.4 APPROVALS

### 8.4.1 ETHICS COMMITTEE APPROVAL

The final protocol and all related documents will be submitted by the Chief Investigator to the IEC that is to undertake ethical review. On receipt of confirmation of the IEC application by the Chief Investigator, the Principal Investigator at each site can complete the site specific information (SSI) form to apply simultaneously for approval from their local IEC and for R&D approval. All approvals must be received in writing by EctoPharma before they will release clinical trial supplies to a site and recruitment can commence. The membership and constitution (written operating procedures) of the IEC will also be obtained.

All substantial revisions (before the start of the trial) and amendments (after the start of the trial) to the protocol must be reported to the IEC from which ethical approval was obtained. A standard notice of amendment form must be submitted. Approval of the revision or amendment must be received in writing before it can be implemented at any site except in the circumstances described in Section 10.3. The Investigator must ensure that progress reports, details of SAEs and any serious protocol violations are provided annually, or more frequently, as required by the IECs. The IECs will be informed should the trial be terminated early.

### 8.4.2 MANAGEMENT APPROVAL

Management approval at the participating site is required before the trial can begin. Application for management approval may be made alongside the application for ethics approval.

## 8.5 TRIAL DOCUMENTATION AND TRIAL CONFIDENTIALITY

### 8.5.1 TRIAL DOCUMENTATION, CRFS AND DOCUMENT KEEPING

The Investigator at each study site must generate and maintain adequate records to enable the conduct of this trial to be fully documented. A record will be made of those items of data for which the CRF is and is not the source.

Each subject enrolled into the trial must have a CRF completed and this must be reviewed and signed off by the Principal Investigator. This also applies to those subjects who fail to complete the trial. Data on CRF pages will be collected manually by the monitor on an ongoing basis and will be forwarded to the Data Centre for entry into the clinical trial database. Data clarification and query resolution will be conducted on an ongoing basis by the monitor and data management. EctoPharma will have overall responsibility for the data.

The Investigator must be aware of his responsibility to retain subject identification codes for 25 years after completion or discontinuation of the trial.

If a subject ceases treatment prematurely, then the reason must be noted in the CRF. If a subject ceases treatment because of an adverse event, reasonable efforts must be made to follow up the event to resolution or stabilisation and to clearly document the outcome in the CRF.

A source data verification plan will be prepared. This will describe the proportion of study data that will be verified by the monitor against the subjects’ source data.

The Investigator will allow representatives of EctoPharma, and personnel acting on the company’s behalf, access on request to all study records including source data that are available outside of the CRFs, such as records of telephone screens etc. Access may also be required by regulatory authorities for the purpose of audit. The Investigator will comply on request with any required background data from such records. This is particularly important when CRFs are illegible or when errors in data transcription are suspected. In the case of special problems and/or regulatory agency queries, it may also be necessary to have access to the complete trial records, provided that subject confidentiality is protected.

Copies of protocols, CRF pages, originals of test results, reports, drug dispensing logs, correspondence, records of informed consent or other documents pertaining to the conduct of the trial must be kept on file by the Investigator for a minimum of 25 years. No trial documents should be destroyed without prior written agreement between EctoPharma and the Investigator. Should the Investigator wish to assign the trial records to another party, or move them to another location, EctoPharma must be informed.

A record must be kept of all subjects considered for the trial and subsequently excluded. This list should include any subject considered eligible but who subsequently did not give informed consent. The reason for non-participation in the trial should be recorded.

The following documents must be provided before or at site initiation. It must be ensured that these documents are in place before a subject is enrolled at the site.

- Signed and dated protocol and amendments (if any).
- Signed and dated IEC approval.
- Approved subject/guardian Information and consent/assent Forms and advertisement for recruitment (if any).
- Regulatory approval.
- CRF.
- Confidentiality agreement.
- Trial products / Shipping records. Trial products should not be delivered to site until the initiation visit and may be delivered after initiation.
- Up to date signed CVs for personnel who have signed the site personnel log (Principal Investigator and trained, qualified, designated assistants).
- Authorised signature log / delegation list.
- Investigator’s Brochure and signed and dated Investigator’s Brochure receipt.
- Signed and dated Clinical Trial Agreement.
- Signed and dated Indemnity/Insurance statement.
- Randomisation codes (code-break envelopes).
- Initiation visit completed (report to be provided for trial files within 15 working days of the visit).
- Pharmacy Agreement (if any).
- Instructions for handling Investigational Product.
- Sample label.
- SAE Forms.

The following documents should be generated during the trial.

- Any amendments to the protocol with relevant approvals.
- Copies of CRFs and data clarification documents.
- Signed consent forms.
- Trial supply records.
- Monitoring visit log.
- All correspondence.
- Subject identification logs.
- SAE report forms.
- Updates to any documents *e.g.* CVs, Investigator’s Brochure *etc*.

### DATA MANAGEMENT

Data collected for the trial will be written into a printed CRF and all data will be sent to the Data Centre. The data will be entered into a clinical database which will undergo verification and validation. A Data Management Plan will be produced to describe the procedures to be undertaken.

### 8.5.3 CONFIDENTIALITY OF TRIAL DOCUMENTATION AND SUBJECT RECORDS

The Investigator must ensure the anonymity of subjects in the trial is maintained. On CRFs, or other documents leaving the centre and submitted to EctoPharma or its designates, subjects must not be identified by name, but by an identification code (usually trial number) and by their initials. The Investigator must keep a separate log of subjects’ codes, names and addresses.

### 8.5.4 CONFIDENTIALITY OF PROPRIETARY INFORMATION

All trial related documentation is confidential, whether obtained by the Investigator or provided by EctoPharma. Disclosure of such information is restricted to those involved in the scientific, ethical and clinical trial procedures.

### 8.5.5 DATA PROTECTION

In order to conform to the requirements of European Union Directive 95/46/EC, subjects will be explicitly asked to consent to transmission of their data outside the European Economic Area.

# 9. PUBLICATION OF DATA

Submission of results for publication will not take place without prior discussion with EctoPharma, allowing the company sufficient time to analyse such results and provide written agreement to publication, which will not be unreasonably withheld. EctoPharma reserves the right to use the results and reports of this study for any purpose.

# 10. ADMINISTRATIVE PROCEDURES

## 10.1 QUALITY ASSURANCE

In accordance with GCP guidelines and recommendations, EctoPharma may undertake an independent quality assurance audit of the clinical trial and related documentation during the course of this study. The purpose of the audit is to determine whether the trial related activities were conducted, and the data were recorded, analysed and accurately reported according to the protocol, relevant Standard Operating Procedures, GCP and the applicable regulatory requirements. At any stage during the study, the Investigator has the responsibility to make all data available to EctoPharma and/or relevant authority (where required) for auditing purposes. Such audits will at all times be conducted in accordance with national, legal and ethical requirements.

## 10.2 MONITORING

At regular intervals during the study, representatives of the monitoring team arranged by EctoPharma Ltd will visit the study centre. The average monitoring frequency will be dependent on the rate of recruitment at each site.

At each monitoring visit, the Investigator and the monitor will review study progress, compliance with the study protocol, CRFs and any emergent problems. The monitor will ensure compliance with the protocol, adherence to regulatory and ICH obligations, accurate reporting of adverse events, maintenance of trial records including study product accountability records and correct administration of trial procedures including supply and storage of trial materials. Consent forms will be reviewed to verify that they are being correctly signed and dated by the subject/guardian and the Investigator.

The monitor will require direct access to all records other than the CRF needed to verify the entries on the CRF. It is the responsibility of the Investigator to ensure that the subject is aware that such access will be required and that this information is available to the monitor.

The Investigator (or his/her designated deputy) agrees to co-operate with the monitor and other clinical research personnel to ensure that any problems detected in the course of these monitoring visits are quickly resolved.

## 10.3 PROTOCOL AMENDMENTS

Any changes to the protocol will be documented as a protocol revision (before the start of the trial) or amendment (after the start of the trial). Revisions/amendments will be submitted for approval to the IEC from which ethical approval for the trial was obtained and to the regulatory authority. Documented approvals of the revision/amendment by the IEC and the regulatory authority must have been received by EctoPharma before being implemented. The exceptions to this are when the change is necessary to eliminate an immediate hazard(s) to the trial subjects or for administrative (non-substantial) amendments involving only logistical or administrative aspects of the trial (*e.g.* change of responsible personnel, change of telephone numbers). Administrative amendments do not need ethical review but should be advised to the IEC. The sponsor is responsible for submitting protocol amendments to the relevant regulatory authority.

## 10.4 ADMINISTRATIVE AND FINANCIAL AGREEMENT

Agreed costs for each participating centre will be met by EctoPharma. For each centre, an agreement will be prepared and signed by the relevant authority on behalf of the institution (*e.g.* NHS Trust, University) and by EctoPharma before the clinical phase of the trial commences.

Subjects will be reimbursed by EctoPharma, through the Investigator, for any travel costs to and from the centre should these be required.

## 10.5 TRIAL SCHEDULE

Recruitment to the trial is planned to commence in July 2007 once all necessary approvals have been granted. It is anticipated that recruitment to the trial will take approximately six months. Including the post-treatment follow-up, subjects will remain in the trial for about two weeks.

## 10.6 END OF TRIAL

The end of the trial is defined as being when all recruited subjects have completed Visit 6, or have withdrawn before completing the trial.

## 10.7 INVESTIGATOR RESPONSIBILITIES

The primary responsibility of all investigators participating in the trial is for the well-being and interests of his/her subjects. The Investigator has overall responsibility for the conduct of the trial at his/her centre and may delegate specific duties to appropriately trained qualified members of his/her research team or to other hospital staff *e.g.* pharmacy. Any delegation must be clearly documented in a centre specific delegation list.

#

# 11. REFERENCES

1. Press Release October 6^th^ 2003. National Public Health Service for Wales
2. Harris, J., Crawshaw, J. G., Millership, S. (2003) Incidence and prevalence of head lice in a district health authority area. *Communicable Disease and Public Health*. **6** (3): 246 – 249.
3. Burgess I. (1991) Malathion lotion for head lice – a less reliable treatment than commonly believed. *Pharmaceutical Journal*; 247:630–632
4. Burgess, I.F.; Brown, C. M.; Peock, S.; Kaufman, J. (1995) Head lice resistant to pyrethroid insecticides in Britain. *British Medical Journal* **311**: 752
5. Downs, A.M.R.; Stafford, K.A.; Harvey, I.; Coles, G. C. (1999) Evidence for double resistance to permethrin and malathion in head lice. *British Journal of Dermatology* **141**: 508-511.
6. Roberts, R. J.; Casey, D.; Morgan, D. A.; Petrovic, M. (2000) Comparison of wet combing with malathion for treatment of head lice in the UK: a pragmatic randomised controlled trial. *Lancet* **356** 540-544.
7. Hill, N. et al. (2005) Single blind, randomised, comparative study of the Bug Buster kit and over the counter pediculicide treatments against head lice in the United Kingdom. *British Medical Journal* **331:** 384 -387.

**APPENDIX 1**

**SAMPLE SUBJECT/GUARDIAN CONSENT/ASSENT FORMS**

**APPENDIX 2**

**SCHEDULE OF TRIAL ACTIVITIES**

**APPENDIX 3**

**SERIOUS ADVERSE EVENT FORM**

1. If no infestation is present at Screening, the subject will be ineligible for the study. [↑](#footnote-ref-1)
