# Supplementary material for: 1,2-Octanediol, a Novel Surfactant, for Treating Head Louse Infestation: Identification of Activity, Formulation, and Randomised, Controlled Trials
Source: PLoS One. 2012 Apr 16;7(4):e35419. doi: 10.1371/journal.pone.0035419 (PMC3327678; doi:10.1371/journal.pone.0035419)
Supplement: Protocol S2 — Protocol CTMK12, prepared on behalf of Thornton & Ross Ltd for Study 3. (DOCX) [file pone.0035419.s006.docx]

**A randomized, assessor blinded, clinical trial to confirm the efficacy of a CE marked class I Medical Device formulation in the treatment of head lice**

#### CTMK12

Version: 1.2, dated 1st November 2010A randomized, assessor blinded, clinical trial to confirm the efficacy of a CE marked class I Medical Device formulation in the treatment of head lice

**Version Number:** Version 1.0, 29^th^ July 2010

**Product Name:** 1,2-octanediol lotion foam

**Country:** UK

**Clinical Study Number:** CTMK12

**Chief Investigator:** Ian Burgess

Medical Entomology Centre, Insect Research and Development Ltd, 6 Quy Court, Colliers Lane, Stow-cum-Quy, Cambridge, CB25 9AU

Tel: +44 1223 813696

Fax: +44 1223 810078

**Clinical Research Manager:** Elizabeth Brunton

Medical Entomology Centre

STUDY MONITOR**:** Harrison Clinical Research Ltd

Ely, Cambridgeshire

**Study Sponsor Representative:** Ashley Brierley

Thornton & Ross Ltd, Linthwaite, Huddersfield,

HD7 5QH

Tel: +44 1484 842217

Fax: +44 1484 847301

**Estimated Start Date:** September 2010

**Estimated Completion Date:** January 2011

"Notice: Part or all of the information contained herein may be unpublished material and should be treated as the confidential property of Thornton & Ross Ltd, not to be divulged to unauthorised persons in any form, including publications and presentations, without the expressed written consent of Thornton & Ross Ltd."

# Investigator's Agreement

We have read this Thornton & Ross Ltd approved protocol, number CTMK12, dated 1^st^ November 2010, entitled "A randomized, assessor blinded, clinical trial to confirm the efficacy of a CE marked class I Medical Device formulation in the treatment of head lice”, and have discussed it to our satisfaction with Thornton & Ross Ltd.

We agree to conduct the study according to this protocol and to comply with its obligations, subject to ethical and safety considerations.

We understand that should we be in breach of any of the terms of this protocol, or if we are negligent, that Thornton & Ross Ltd would not be held responsible for any resulting losses, damages, costs and expenses of whatever kind made by or on behalf of a participant.

Chief Investigator:

________________________Dated ___/___/___

Ian Burgess

Clinical Research Manager:

________________________Dated ___/___/___

Elizabeth Brunton

Thornton & Ross Ltd

Study Sponsor Representative: ________________________Dated ___/___/___

Ashley Brierley

Should the decision be made by Thornton & Ross Ltd to terminate the study at any time, such decision will be communicated to the Investigator in writing, and appropriate arrangements will be agreed upon and specified in writing. Conversely, should the Investigator decide to withdraw from execution of the study he/she will communicate immediately such decision in writing to Thornton & Ross Ltd.

**Contents**

[Investigator's Agreement 3](#_Toc268172662)

[1. Introduction 6](#_Toc268172663)

[1.1 Summary of the Study 6](#_Toc268172664)

[1.2 Rationale 7](#_Toc268172665)

[1.3 Aims (Objectives) 8](#_Toc268172666)

[Design in Brief 8](#_Toc268172667)

[2. Materials and Methods 9](#_Toc268172668)

[2.1 Participant Selection 9](#_Toc268172669)

[2.1.1 Total Numbers of Participants and Study Duration 9](#_Toc268172670)

[2.1.2 Inclusion Criteria 10](#_Toc268172671)

[2.1.3 Exclusion Criteria 10](#_Toc268172672)

[2.2 Clinical Supplies and Materials 10](#_Toc268172673)

[2.2.1 Physical Forms of the Study Supplies 10](#_Toc268172674)

[2.2.2 Packaging and Labelling 11](#_Toc268172675)

[2.2.3 Care of Supplies 11](#_Toc268172676)

[2.2.4 Study Materials 11](#_Toc268172677)

[2.2.5 Compliance 11](#_Toc268172678)

[2.3 Procedures and Investigations 11](#_Toc268172679)

[2.3.1 Treatment Regimen/Allocation 12](#_Toc268172680)

[2.3.2 Randomisation 12](#_Toc268172681)

[2.3.3 Study Methodology 12](#_Toc268172682)

[2.3.3.1 Pre-recruitment 12](#_Toc268172683)

[2.3.3.2 Recruitment (Day 0) 13](#_Toc268172684)

[2.3.3.3 Follow up Assessments (Days 1, 6 and 9) 15](#_Toc268172685)

[2.3.3.4 Follow up Treatment (Day 7) 15](#_Toc268172686)

[2.3.3.5 Final Assessment (Day 14) 15](#_Toc268172687)

[2.3.4 Concomitant Medication 16](#_Toc268172688)

[2.3.5 Adverse Events 16](#_Toc268172689)

[2.3.6 Serious Adverse Events 16](#_Toc268172690)

[2.3.7 Withdrawals 17](#_Toc268172691)

[3. Analysis and Reports 18](#_Toc268172692)

[3.1 Definition of End Points 18](#_Toc268172693)

[3.1.1 Safety 18](#_Toc268172694)

[3.1.2 Efficacy 18](#_Toc268172695)

[3.2 Definition of Populations to be analysed 19](#_Toc268172696)

[3.3 Proposed Primary and Secondary Analyses 20](#_Toc268172697)

[3.4 Statistical Methods 20](#_Toc268172698)

[3.5 Final Study Report 21](#_Toc268172699)

[4 Administrative Procedures 21](#_Toc268172700)

[4.1 Regulatory Documentation 21](#_Toc268172701)

[4.2 Ethics Committee Approval 21](#_Toc268172702)

[4.3 Informed Consent 22](#_Toc268172703)

[4.4 Insurance Policy 22](#_Toc268172704)

[4.5 Compensation 22](#_Toc268172705)

[4.6 Investigator's Responsibilities 22](#_Toc268172706)

[4.7 Curriculum Vitae 23](#_Toc268172707)

[4.8 Case Record Form 23](#_Toc268172708)

[4.9 Monitoring of the Study 24](#_Toc268172709)

[4.10 Quality Assurance 24](#_Toc268172710)

[4.11 Protocol Appendices 24](#_Toc268172711)

[4.12 Protocol Amendments 24](#_Toc268172712)

[4.13 Publication Policy 25](#_Toc268172713)

[4.14 Early Termination of the Study 25](#_Toc268172714)

###### Appendices

Appendix 1 Declaration of Helsinki.

Appendix 2 European (CPMP) Guidelines on Good Clinical Practice for Trials on Medicinal Products.

# Introduction

## 1.1 Summary of the Study

Title: A randomized, assessor blinded, clinical trial to confirm the efficacy of a CE marked class I Medical Device formulation in the treatment of head lice

**Chief Investigator:** Ian F Burgess

**Estimated Study Start:** September 2010

**Estimated Study Finish:** January 2011

**Participants:** One hundred and twenty (120) evaluable participants will be recruited to the study, randomised between three groups and treated with two vairants of the same CE marked product 5% 1,2-octanediol lotions.

**Type:** Children (aged 6 months and over) and adults who, upon inspection, are found to have live head lice.

**Products:** KindaPed AF (5% 1,2-octanediol alcohol free lotion)

KindaPed (5% 1,2-octanediol lotion with 20% alcohol).

**Methods of Application:** KindaPed AF 5% 1,2-octanediol alcohol free lotion (2-2.5 hours) and KindaPed 5% 1,2-octanediol lotion: These products will be applied directly to dry hair. Sufficient product will be applied to saturate the hair and scalp. The product will be left in place for 2 hours (maximum 2.5 hours) before being removed with proprietary shampoo. The product will be reapplied at Day 7.

KindaPed AF 5% 1,2-octanediol alcohol free lotion (8 hours or overnight): This product will be applied directly to dry hair. Sufficient product will be applied to saturate the hair and scalp. The product will be left in place for a minimum of 8 hours, or left on overnight, before being removed with proprietary shampoo. The product will be reapplied at Day 7.

**Study Design:** This is a randomised, assessor blind clinical investigation designed to confirm efficacy in use and obtain further information about routine usage of two variants of the same CE marked class I Medical Device product with the same active principle in the control and elimination of head louse infestation.

Participants will be assessed at Day 0 (recruitment) and then treated. An investigator will apply the treatment. The same treatment will be reapplied on Day 7.

Participants will be assessed for the presence of live head lice on Days 1, 6, 9 and 14 (+/- 1 day). The assessors will be blind to the treatment group. Any lice found at assessments will be taped into the Case Record Form (CRF) for size analysis.

**Aims of the Study:** To obtain directly comparative information to show that KindaPed AF 5% 1,2-octanediol alcohol free lotion is equally effective in the eradication of head lice as KindaPed 1,2-octanediol lotion.

## 1.2 Rationale

Infestation with the human head louse (*Pediculus capitis*) has been at a high level within the European Union since the appearance of strains of head lice resistant to one or more of the currently available insecticidal products were first described (1, 2, 3). Families are aware of a resistance problem but have no way of knowing if the lice they are trying to eradicate are resistant to the product they are using. Many people have attempted alternative methods of treatment such as combing (4) but for the most hard pressed families (as described in reference 3) this frequently has a poor outcome with children suffering infestations for many months (5).

Given a choice, a majority of care givers would choose to use a safe and simple chemical treatment to eliminate infestation form their children. Various products have been introduced into the European market as class I Medical Devices based on their physical mode of action and their general safety profile.

KindaPed (5% 1,2-octanediol lotion) and KindaPed AF (5% 1,2-octanediol alcohol free lotion) are class I Medical Device preparations based upon a cosmetic moistening and spreading agent.

The original KindaPed (5% 1,2-octanediol lotion) was originally investigated in a large randomised controlled trial in 2007-08 in comparison with 0.5% malathion liquid. In this study KindaPed was shown to be significantly superior to malathion using applications times of 2-2.5 hours and 8 hours/overnight. However, the KindaPed preparation was formulated with 20% isopropanol, which it is believed was the cause of a number of mild adverse reactions on excoriated skin.

KindaPed AF (5% 1,2-octanediol alcohol free lotion) contains no alcohol and should not cause irritation even on excoriated skin. This clinical investigation is designed to confirm no loss of efficacy of Kindaped AF versus Kindaped.

Both 5% 1,2-octanediol lotion preparations are used with a repeat treatment applied after one week as recommended by The British National Formulary (BNF) for all currently available treatments.

##### References

1. Burgess IF, Brown CM, Peock S and Kaufman J. Head lice resistance to pyrethroid insecticides in Britain. British Medical Journal, 1995; 311: 752.
2. Downs AM, Stafford KA, Harvey I and Coles G. Evidence for double resistance to permethrin and malathion in head lice. British Journal of Dermatology, 1999; 141: 508-511.
3. Willems S, Lapeere H, Haedens N, Pasteels I, Naeyaert J-M, De Maeseneer J. The importance of socio-economic status and individual characteristics on the prevalence of head lice in schoolchildren. European Journal of Dermatology 2005; 15: 387-392.
4. Hill N, Moor G, Cameron MM, Butlin A, Preston S, Williamson MS, Bass C. Single blind, randomised, comparative study of the Bug Buster kit and over the counter pediculicide treatments against head lice in the United Kingdom. British Medical Journal 2005; 331: 384-387.
5. Burgess IF, Brown CM, Lee PN. Treatment of head louse infestation with 4% dimeticone lotion: randomised controlled equivalence trial. British Medical Journal 2005; 330: 1423–1425.

## 1.3 Aims (Objectives)

1. To compare efficacy of KindaPed AF (5% 1,2-octanediol alcohol free lotion ) with KindaPed (5% octanediol lotion with 20% alcohol) for elimination of infestation with head lice.
2. To compare KindaPed AF (5% 1,2-octanediol alcohol free lotion ) with KindaPed (5% octanediol lotion with 20% alcohol) with regard to prevention of louse egg hatching (ovicidal action) by monitoring the presence/absence of juvenile stages of lice following the first and second treatments.
3. To compare KindaPed AF (5% 1,2-octanediol alcohol free lotion ) with KindaPed (5% octanediol lotion with 20% alcohol) To investigate 5% 1,2-octanediol alcohol free lotion foam with regard to safety and participant acceptability.

## Design in Brief

This is a randomised, controlled, assessor blind, study of two variants of the same class I Medical Device product for control of head louse infestation. One hundred and twenty (120) evaluable participants, minimum age 6 months^[[1]](#footnote-1)^, who upon examination are confirmed to have head lice and who fit the selection criteria (see section 2.1.2 and 2.1.3) will be recruited to the study.

At the first visit (Day 0), verbal consent will be obtained to check for the presence of live head lice using a fine-toothed plastic detection comb. After the preliminary assessment, participants can be enrolled to the study, provided they comply with the inclusion/exclusion criteria, and any further questions they may have are fully dealt with.

Participants (or their parents/guardians if they are aged below 16 years) will be asked to give written informed consent and sign a Consent Form before participation in the study. There will be a separate Assent Form for younger children capable of giving written assent. Children too young to give assent can be entered in the study on the consent of a parent/guardian. Assent Forms will be witnessed by the parent/guardian and signed by the Investigator. Consent will be sought for permission to inform the participant’s GP that their patient is taking part in the study. Other infested household members who are unable to participate in the study for any reason, e.g. not being available for the assigned follow up visits, will be offered a standard of care treatment (Hedrin 4% liquid gel) in order to reduce the risk of reinfestation of participants during the course of the study.

The Investigator will assign each participant a study number, this being the next available number allocated to that investigator from a randomised treatment allocation sequence. Participants will be divided between three groups as follows:

Group A: KindaPed AF (5% 1,2-octanediol alcohol free lotion ), applied for 2 hours (maximum 2.5 hours) before washing off using shampoo, with a repeat treatment one week later.

Group B: KindaPed AF (5% 1,2-octanediol alcohol free lotion ), applied for 8 hours (minimum) or overnight before washing off using shampoo, with a repeat treatment one week later.

Group C: KindaPed lotion (5% 1,2-octanediol lotion with 20% alcohol), applied for 2 hours (maximum 2.5 hours) before washing off using shampoo, with a repeat treatment one week later.

Participants will be treated at Day 0 and Day 7 (+/- 1 day). A member of the study team will apply the treatment on both occasions. Participants will be assessed on Days 1, 6, 9 and 14 (+/- 1 day at each assessment) by a different member of the study team. Any lice found at assessments will be taped into the participant’s CRF.

All adverse events will be monitored during the study (see sections 2.3.5 and 2.3.6) and all changes in concomitant illness and medication will be recorded (see section 2.3.4). A Completion/Withdrawal Form will be completed at the end of the study period.

# 2. Materials and Methods

## 2.1 Participant Selection

### Total Numbers of Participants and Study Duration

One hundred and twenty (120) evaluable participants will be recruited to the study, randomised between three groups. The duration of the study will be 15 days (+/- 1 day), Day 0 being the first treatment day, with 14 days follow up.

### 2.1.2 Inclusion Criteria

1. Children and adults aged 6 months^[[2]](#footnote-2)^ and over of both sexes with no upper age limit.
2. People who upon examination, are confirmed to have live head lice.
3. People who give written informed consent, or if the participant is under 16 years of age whose parent/guardian gives written informed consent to participate in the study.
4. People who will be available for follow up visits by study team members over the 14 days following first treatment.

### 2.1.3 Exclusion Criteria

1. People with a known sensitivity to any of the ingredients in KindaPed AF (5% 1,2-octanediol alcohol free lotion) or in KindaPed lotion (5% 1,2-octanediol lotion with 20% alcohol).
2. People with a secondary bacterial infection of the scalp (e.g. impetigo) or who have an active long term scalp condition (e.g. psoriasis of the scalp).
3. People who have been treated with other head lice products within the previous two weeks.
4. People who have bleached hair, or hair that has been permanently waved within the previous four weeks.
5. People who have been treated with the antibiotics Co-Trimoxazole or Trimethoprim within the previous four weeks, or who are currently taking such a course.
6. Pregnant or nursing mothers.
7. People who have participated in another clinical study within 1 month before entry to this study.
8. People who have already participated in this clinical study.

## 2.2 Clinical Supplies and Materials

### 2.2.1 Physical Forms of the Study Supplies

KindaPed AF (5% 1,2-octanediol alcohol free lotion):

Active: 1,2-octanediol 5%

Excipients: Polyethylene glycol 6, water.

KindaPed lotion (5% 1,2-octanediol lotion):

Active: 1,2-octanediol 5%

Excipients: Sodium dodecyl sulphate 0.64%, propan-2-ol (isopropanol) 20%, water.

### 2.2.2 Packaging and Labelling

###### Packaging:

###### The products will be packed in plastic foamer bottles with each container holding 100mL of material. The bottles will be made from high-density polyethylene (HDPE) with screw fit, finger pump foamer nozzles.

Labelling:

The bottles of test product used in the study will be numbered and weighed on calibrated scales before use. A clinical trial label will be affixed identifying the individual bottle number and a blank section will be provided for completion of participant number and initials. The product will be labelled with appropriate clinical trial labelling that will also state that it is “For Clinical Trial Use Only”.

### 2.2.3 Care of Supplies

All supplies used in the study must be maintained securely under the direct responsibility of the Chief Investigator or under that delegated by the Investigator.

All supplies shall be dispensed in accordance with the Investigator's direction and it is the Investigator's responsibility to ensure an accurate record of supplies issued and returned is maintained.

All supplies should be stored at room temperature, out of direct sunlight and protected from extremes of environmental conditions.

All supplies will be used only while participating in the study and returned to MEC at the end of the study for weighing before being returned to Thornton & Ross Ltd.

### 2.2.4 Study Materials

Thornton & Ross Ltd will supply all the clinical study materials required for the duration of the study. In addition, numbered CRFs will be supplied for each participant.

### 2.2.5 Compliance

All supplies used, partly used, or unused will be maintained for collection by the study monitor.

## 2.3 Procedures and Investigations

### 2.3.1 Treatment Regimen/Allocation

This is a randomised, controlled, comparative study of KindaPed AF (5% 1,2-octanediol alcohol free lotion) in comparison with KindaPed lotion (5% 1,2-octanediol lotion with 20% alcohol) in the treatment of head lice. Each participant who satisfies the inclusion/exclusion criteria and agrees to participate will be randomised to one of three groups. Two groups will be treated with KindaPed AF (5% 1,2-octanediol alcohol free lotion) but using different application times (2-2.5 hours or 8 hours/overnight), the third group will be treated with KindaPed lotion (5% 1,2-octanediol lotion with 20% alcohol).

### 2.3.2 Randomisation

The randomised treatment allocation code will be generated using the free online randomisation service provided at <http://www.randomization.com/>. The treatment allocation will be made in 11 balanced blocks of 12 treatments and the Seed number and date of randomisation will be recorded on the randomisation plan.

The treatment allocations will be prepared as sheets bearing the identification of the product to be used and instructions for application of the relevant product. The product identification/instruction sheets will be sealed in opaque envelopes numbered sequentially on the outside with the participant number taken from the randomisation schedule. Each envelope will be enclosed in a CRF prior to use. Each investigator delegated to enrol participants will allocate the numbered CRFs to participants in numerical sequence. However, where two or more investigators, each allocated separate blocks of numbers, enrol participants in parallel the overall numerical sequence will not follow chronologically.

Only after written informed consent has been obtained will the investigator allocate a study number and open the randomisation envelope for that number. The investigating team will keep a second batch of envelopes, and a copy of the randomisation code also in a sealed envelope, in case an adverse event, reaction, or any other emergency circumstance necessitates that the code be broken.

### 2.3.3 Study Methodology

2.3.3.1 Pre-recruitment

Participants will be invited to join the study via advertising in public media (newspapers, magazines, as appropriate), via posters in schools, pharmacies, GP surgeries, and through letters or telephone calls to families who have expressed an interest in joining further clinical investigations and are listed on a database held at the Medical Entomology Centre. A detailed Participant Information Booklet (PIB) will be provided to explain the purpose of the study. This will include a children’s section explaining what will happen if the person enters the study.

A member of the study team will conduct a brief interview to establish whether the person will be suitable for entry into the study. If the person wishes to enter the study, they will be conducted through a standardised consent procedure. Potential participants must have had access to the PIB for at least 24 hours before the recruitment takes place.

2.3.3.2 Recruitment (Day 0)

Screening:

Each potential participant will be asked for permission to assess their hair to confirm the presence of live head lice. The assessment will be made by dry combing the hair with a plastic fine-toothed head louse detection comb. Lice found during the assessment will not be removed. Other family members who give their verbal permission can also be assessed for the presence of living lice and may join the study provided they meet the inclusion/exclusion criteria.

Details will be recorded of how many people share the place of residence with the participant. Details will also be recorded of the number of people assessed and found to have lice, the number assessed and found not to have lice and the number that were not assessed. The number of people enrolled in the study will also be recorded.

Consent/Assent:

Participants and/or parents/guardians will be asked if they understand the requirements of the study and if they have any further questions concerning it. Provided they still wish to enter the study and meet the inclusion/exclusion criteria for entry, the participant or parent/guardian (when the participant is below the age of 16) will read and sign the Consent Form. The Investigator will countersign the Consent Form.

A separate Assent Form will be available for those under the age of 16 provided they are capable of signing their name. The Investigator and the parent/guardian will countersign the Assent Forms.

Children too young to provide signed assent may be enrolled on the basis of parental consent.

Case Record Form completion:

Personal data allowing identification of an individual will not be recorded in the CRF. However, as there are no source medical documents (i.e. patient medical records) available to the investigators a Source Data Verification Sheet will be completed for each participant that will be maintained separately from all other study documentation. This Sheet will include information such as name, date of birth, address, and contact details for the General Practitioner.

The following information will be recorded in the CRF:

1. **Declaration of Receipt of Informed Consent**: Confirmation that informed consent and assent (where relevant) was obtained, that a copy of the consent has been given to the participant and/or parent/guardian and that the original will be retained.
2. **Identification**: Participant's study number and age.
3. **Hair Characteristics**: Characteristics will be recorded of the participant’s hair:
   1. Length: closely cropped, above ears, ears to shoulders, below shoulders
   2. Thickness: fine, medium, thick
   3. Degree of curl: straight, wavy, slight curl, tight curl
   4. Type: dry, normal, greasy
4. **Head Lice Details**: When the participant was last treated for head lice (an exact date if treated within the previous four weeks), the treatment that was used and the outcome (success or failure). The severity of the current louse infestation will be assessed using the following scale:
   1. Light infestation: single louse found only after 5-6 combs of the hair
   2. Moderate infestation: single louse found on the first comb of the hair
   3. Heavy infestation: more than one louse found on the first comb of the hair
5. **Medication Current at Entry**: Any medication being taken along with the date the medication started the total dose and the reason for the medication.
6. **Medical History**: Medical history and any current illnesses will be recorded.
7. **Inclusion/Exclusion Criteria**: Confirmation that the participant meets the inclusion/exclusion criteria for entry into the study.

Randomisation:

The Investigator will carry a block of sequential numbered envelopes, which correspond to the randomisation numbers on the CRFs. Each envelope will contain a randomised treatment allocation. After consent has been received, the next sequential numbered envelope will be opened and the specified treatment allocated.

Treatment:

A member of the study team will apply the treatment to the participant and the participant number and participant initials will be added to the label of the bottle used. Any unused product will be retained and returned to so that the weight can be recorded and the amount of product used calculated.

The products will be applied in the following way:

**KindaPed AF (5% 1,2-octanediol alcohol free lotion) 2-2.5 hours** will be applied directly to dry hair. Sufficient product will be applied to wet all the hair and scalp. More than one bottle can be used if required. The product will be left in place for 2 hours, however for practical purposes and convenience of the participants a small flexibility has been included in the treatment time so that the product may remain on the hair for up to 2.5 hours. The product will then be washed off using non-medicated shampoo. The hair will then be rinsed with water. Hair can be dried in the usual way following hair washing after treatment.

**KindaPed AF (5% 1,2-octanediol alcohol free lotion) 8 hours or overnight** will be applied directly to dry hair. Sufficient product will be applied to wet all the hair and scalp. More than one bottle can be used if required. The product will be left in place for at least 8 hours, if applied early enough during the day, but if applied late it will be left on overnight. The product will then be washed off using non-medicated shampoo. The hair will then be rinsed with water. Hair can be dried in the usual way following hair washing after treatment.

**KindaPed lotion (5% 1,2-octanediol with 20% alcohol)** will be applied directly to dry hair. Sufficient product will be applied to wet all the hair and scalp. More than one bottle can be used if required. The product will be left in place for 2 hours, however for practical purposes and convenience of the participants as small flexibility has been included in the treatment time so that the product may remain on the hair for up to 2.5 hours. The product will then be washed off using non-medicated shampoo. The hair will then be rinsed with water. Hair can be dried in the usual way following hair washing after treatment.

In all cases the participants will be reminded that a second treatment will be applied on Day 7.

Any household members who have lice but are unable to participate in the study for any reason will be offered a standard of care treatment (Hedrin 4% liquid gel) in order to reduce the risk of reinfestation of participants during the course of the study.

2.3.3.3 Follow up Assessments (Days 1, 6 and 9)

Assessments will take place on Days 1, 6, and 9 (+/- 1 day) – see section 2.3.3.5 for Day 14. At assessments, the participant’s hair will be combed with a head louse detection comb, and any lice found will be taped (with clear cellulose tape) into the participant’s CRF. In order to provide blinding and to minimise bias the study team member that conducts the follow up assessments will be different from the team member that applied the treatment at Day 0 and 7.

All adverse events and changes in concomitant medication will be recorded in the CRF.

- - - 1. Follow up Treatment (Day 7)

Treatment will be reapplied at Day 7 following the same procedure as at Day 0 (see section 2.3.3.2).

- - - 1. Final Assessment (Day 14)

The final assessment will take place 14 days (+/- 1 day) after the first treatment (the 15^th^ day of the study). The participant’s hair will be combed with a head louse detection comb and any lice found will be taped (with clear tape) into the participant’s CRF.

Participants who still have live lice at the end of the study will be offered a standard of care treatment (Hedrin 4% liquid gel).

All adverse events and changes in concomitant medication will be recorded in the CRF.

At the Day 14 assessment, the participant and/or parent/guardian will be asked to complete a questionnaire on the treatment that was applied at Day 0. This will include questions on how the participant’s scalp felt, how the hair felt, how easily the product washed out, and how convenient the participant and/or parent/guardian considered this treatment application time.

The Completion/Withdrawal Form will then be completed.

**2.3.3.6 Assessment Analysis**

Any lice found during the course of the study will be examined under the microscope to establish the sex and/or stage of development of the insects. It is expected that few adult lice will be found during the monitoring period. The presence of small lice (nymphs) will be evidence that not all of the eggs were killed by the first treatment and will be used as a measure of ovicidal activity. Continued monitoring enables the investigation to determine whether the presence of any lice is due to surviving lice or surviving eggs from which nymphs emerge. The occasional adult louse (1 or 2 only) found after the initial infestation is cleared would be indicative of re-infection (see section 3.1.2).

### 2.3.4 Concomitant Medication

The participant should not use any other form of pediculicide treatment while taking part in the study. If the use of such treatment occurs, the participant will be withdrawn from the study.

Other medication can be prescribed in the normal way although participants requiring Co-Trimoxazole or Trimethoprim should be withdrawn from the study.

All concomitant medicines should be listed in the CRF and any changes to such medicines, during the course of the study, recorded.

### 2.3.5 Adverse Events

Space will be provided in the CRF specifically for recording observed and reported adverse events. All unwanted effects, whether considered to be caused by the study medication or not, will be reported to Thornton & Ross Ltd by completing the Adverse Event form.

### 2.3.6 Serious Adverse Events

If the adverse event is serious, it shall be reported immediately, by e-mail and telephone and by facsimile to the Medical Contact and Thornton & Ross Ltd. A full written report will be forwarded to Thornton & Ross Ltd, by facsimile, within 3 working days.

Serious adverse events are defined as events that are fatal, life threatening, disabling or incapacitating, cause or prolong hospitalisation, overdose (of any kind, with or without symptoms), newly diagnosed cancer or clinically abnormal laboratory values (with or without symptoms).

The contacts for all serious adverse events are:

| **Study Sponsor contact**:  Dr Joanne Talbot  Pharmacovigilance and Medical Information Department  Thornton & Ross Ltd  Linthwaite  Huddersfield  HD7 5QH  Tel: +44 1484 848251 - Direct line with voicemail.  +44 1484 842217 - Main switchboard.  Fax: +44 1484 847301 - Address to “Pharmacovigilance and Medical Information Department” and mark as **Urgent**  E: [phv@thorntonross.com](mailto:steveskilleter@thorntonross.com) or [joannetalbot@thorntonross.com](mailto:joannetalbot@thorntonross.com) |
| --- |
| **Local Medical contact**:  Dr Paul Silverston  Oakfield Surgery,  Vicarage Road,  Newmarket,  Suffolk, CB8 8HP  Tel: 01638 662018  Fax: 01638 660294 |

### 2.3.7 Withdrawals

Participants may be withdrawn from the study at any time for the following reasons:

Adverse Event:

The participant is withdrawn from the study by the Investigator because of an adverse event, whether or not the Investigator believes it to be serious or caused by the study medication, and provided that the Investigator considers it is in the participant's best interest to be withdrawn. There must be a corresponding entry on the Adverse Events and/or the Serious Adverse Events Form in this instance.

Non-compliance:

The participant is withdrawn because of failure to comply with the treatment regimen, or comply with the investigations as required, but is still accessible to the Investigator.

Drop Out:

The participant withdraws consent to continue in the study, but the Investigator would otherwise consider it appropriate for him/her to continue. The participant remains accessible to the Investigator.

Lost to Follow-up:

The participant, without explanation, fails to keep appointments as scheduled for study assessments and is not seen again despite the Investigator's effort (letter, telephone, home visit etc.) to re-establish contact.

Death:

All deaths will be treated as Serious Adverse Events and Thornton & Ross Ltd must be informed within 24 hours. All associated documentation must be completed within 3 working days. Full details will be required including a post-mortem examination if possible.

Lack of Efficacy:

The participant elects to withdraw, because the medication is not adequately effective.

# 3. Analysis and Reports

## 3.1 Definition of End Points

### 3.1.1 Safety

Participants will be observed and all untoward effects will be recorded, whether or not they are thought to be related to the study treatment.

Details of the recording of adverse events are shown in section 2.3.5 and 2.3.6.

### 3.1.2 Efficacy

The primary endpoint is cure, indicated by no evidence of active head louse infestation following the second application of treatment, or cure followed by reinfestation, indicated by limited numbers of adult or stage 3 nymphs on Days 9 or 14, as set out in the following treatment outcome algorithm.

The secondary endpoints are to confirm whether the treatment inhibits louse eggs from hatching (as demonstrated by no stage 1 or 2 nymphs after the first treatment) and the safety and acceptability of the treatment regimen.

**Treatment Outcome Algorithm**

This algorithm has been developed by Medical Entomology Centre and P. N. Lee Statistics and Computing Ltd in order to impose limits within which the outcomes of treatment and any lice recovered can be interpreted.

Cure:

No lice present on Day 9 and Day 14.

Re-infestation

The following must all apply:

- - - Lice must be present at Day 9 and/or Day 14.
    - Adult lice and/or stage 3 nymphs must not be present at Days 1, 2 or 6.
    - Stage 1 or 2 nymphs must not be present at Day 9 or Day 14.
    - If stage 3 nymphs are present at Day 9 there must be no stage 1 or 2 nymphs present at Day 6.
    - Stage 3 nymphs may be present at Day 9 or Day 14 although they must not exceed 2* in number.
    - Adult lice should not exceed 2* at either Day 9 or Day 14.

In addition, the following may apply:

- If adult lice are present at Day 9 Stage 1 and stage 2 nymphs may be present at Day 6.

* The number 2 is an interpretation of the limits of “occasional”.

Ovicidal Failure Only

Ovicidal failure is evidenced by the presence of stage 1 nymphs within 2 days of the second treatment administered on Day 7

For ovicidal failure only, specific lice must not be present at Day 9 or Day 14:

- - - At Day 9 there must be no adult lice and no stage 2 or stage 3 nymphs.
    - At Day 14 there must be no adult lice or stage 3 nymphs.

This is equivalent to requiring stage 1 nymphs only at Day 9 and/or stage 1 and/or stage 2 nymphs only at Day 14.

Licidal Failure Only

All other outcomes are licidal failures.

For licidal failure only, there must be no stage 1 nymphs at Day 9 and no stage 1 or stage 2 nymphs at Day 14, so as not to satisfy the condition for ovicidal failure.

Licidal and Ovicidal Failure

Any subject not falling into groups 1, 2, 3 or 4, including those with missing values for the data necessary to make the classification into groups 1-4. This includes any subjects with missing data at days 9 or 14, as well as those subjects with evidence of lice at days 9 or 14, but missing data at days 2 or 6.

.

## 3.2 Definition of Populations to be analysed

The Per-protocol Population:

Includes all participants treated according to the study protocol.

"Intention-to-treat" Population:

Includes all participants consenting and treated at least once. Premature terminations, due to treatment failure, adverse events etc., are included.

## 3.3 Proposed Primary and Secondary Analyses

1. To compare efficacy of KindaPed AF (5% 1,2-octanediol alcohol free lotion) with KindaPed (5% octanediol lotion with 20% alcohol) for elimination of infestation by head lice.
2. To compare KindaPed AF (5% 1,2-octanediol alcohol free lotion) with KindaPed (5% octanediol lotion with 20% alcohol) with regard to prevention of louse egg hatching (ovicidal action) by monitoring the presence/absence of juvenile stages of lice following the first and second treatments.
3. To compare KindaPed AF (5% 1,2-octanediol alcohol free lotion) with KindaPed (5% octanediol lotion with 20% alcohol) To investigate 5% 1,2-octanediol alcohol free lotion with regard to participant acceptability.

## 3.4 Statistical Methods

Sample Size Determination:

The study has been designed to confirm that KindaPed AF (5% 1,2-octanediol alcohol free lotion) has an essentially similar efficacy comparable to that of KindaPed lotion (5% 1,2-octanediol with 20% alcohol). It is not designed as a full equivalence study but is designed to be able to identify any significant differences between the treatments in pair wise comparisons.

This study is based upon the same sample size as that used in four previous studies of head louse treatments conducted on behalf of Thornton & Ross Ltd (CTMK06, CTMK08, CTMK09, and CTMK10) for basic comparative purposes and is estimated to provide adequate data for demonstration of effect. The actual sample size of 40 per group makes allowance for dropout. Comparison of the results from this study can be made with the results from those studies with respect to similarities of population demographics, and with previous studies of 1,2-octandiol lotion (CTEP01 and CTEP02) with respect to efficacy outcome and acceptability.

Interim analysis

It is proposed to conduct an interim analysis of treatment outcome only after enrolment and completion of sixty (60) participants (approximately 20 in each group). This analysis will be conducted blind of treatment allocation using outcome data entered into a spreadsheet which would then be overlaid with treatment allocation identified only as “a”, “b”, or “c”. This will enable the Sponsor to identify whether one treatment is performing exceptionally well or exceptionally poorly and allow them to make a decision in good time whether they wish to extend or to curtail any aspect of the study. The results of this analysis will be conveyed to the Sponsor in narrative form only.

Analytical Methods:

The primary endpoint is the frequency of cure tested using the "intention-to-treat" population in comparison with the body of data obtained during previous studies using overnight or 2-2.5 hour application times for the product. Secondary endpoints will be the frequency of ovicidal failure and of reinfestation and the numbers of lice seen at the different assessment times. Other endpoints will be the safety of the product and participant acceptance.

Analyses will be conducted based on both the "intention-to-treat" and the "per-protocol" populations. The study group will be compared with study groups from previous studies in which participants were treated using an 8 hour/overnight treatment or a 2-2.5 hour treatment on baseline characteristics, safety, acceptability, and efficacy using Fisher's exact test for yes/no variables and the Mann-Whitney U test for ranked variables. Where analysis shows important differences in baseline characteristics, chi squared and rank tests stratified for these characteristics may also be conducted. 95% confidence limits will be presented for the difference between this study group and previous groups in the primary endpoint.

These analyses will be used to determine whether the study provides evidence of essential similarity between KindaPed AF (5% 1,2-octanediol alcohol free lotion) and KindaPed (5% 1,2-octanediol lotion) in the elimination of infestation.

## 3.5 Final Study Report

A clinical report, integrating the study design and the results will be prepared for the study and agreed by the Chief Investigator and the Study Managers. The Chief Investigator, the Clinical Research Manager, and representatives of Thornton & Ross Ltd will sign a copy of the final study report.

# 4 Administrative Procedures

## 4.1 Regulatory Documentation

Any required legislative procedures will be undertaken before the commencement of the study. The study will not proceed without granted written approval.

This study will be conducted according to the recommendations of the European (CPMP) Guidelines on Good Clinical Practice for Trials on Medicinal Products and with the European Standard, and the EU Directive on Good Clinical Practice (2001/20/EU).

## 4.2 Ethics Committee Approval

The Chief Investigator will be required to obtain the written approval of the relevant Research Ethics Committee before commencing the study. In accordance with Good Clinical Research Practice, a copy of this approval together with the constitution of the ethics committee will be forwarded to Thornton & Ross Ltd before the release of trial supplies from Thornton & Ross Ltd.

## 4.3 Informed Consent

This study will be conducted in accordance with the principles laid down in the Declaration of the World Medical Assembly of Helsinki, and subsequent revisions (see Appendix 1).

Each participant or parent/guardian (where the participant is not legally competent) will be requested to provide written informed consent after receiving written information and a verbal explanation of what the study involves. A copy of the Consent Form will be returned to the participant and/or parent/guardian.

The Investigator will retain the original of the Consent Form, but will also complete a Declaration of Receipt of Consent Form to confirm that written informed consent was obtained. The Investigator shall arrange for the retention of participant identification codes for at least 25 years after the completion or discontinuation of the study.

## 4.4 Insurance Policy

Thornton & Ross Ltd confirms that this specific clinical study is protected by insurance cover which provides an indemnity to the Investigators and their co-workers, subject to the Policy terms, conditions and limitations and provided always that the study is conducted and the data as reported agree to the standards fixed by the protocol. Indemnity, in the event of negligent acts by investigators in the field, must be covered by the professional liability insurance of the appropriate institution employing them.

## 4.5 Compensation

Thornton & Ross Ltd maintains in force a "no fault" compensation insurance indemnity in accordance with the current version of the ABPI Guidelines on Clinical Trials: "Compensation for Medicine Induced Injury". In the event that the compensation on a "no fault basis" is unacceptable to the claimant, the Policy will, subject to its terms, conditions and limitations, respond to an action for legal liability arising out of this clinical study.

## 4.6 Investigator's Responsibilities

##### Good Clinical Practice

It is the responsibility of the Investigators to ensure that this study is carried out in accordance with this protocol in respect of ethical, legal and technical aspects and conforming to the European (CPMP) Guidelines on Good Clinical Practice for Trials on Medicinal Products. In this context, the Investigator shall arrange for the retention of participant identification codes for at least 25 years after completion or discontinuation of the study. Thornton & Ross Ltd will render all support necessary to assist the Investigator in discharging this responsibility.

##### Replacement of Chief/Principal Investigator

In the event of a Chief/Principal Investigator being unable to continue the study, another responsible person may be designated Investigator and documentation testifying to this will be submitted to the study monitor and the Research Ethics Committee within 10 days. The new Investigator must be appropriately qualified and be approved by Thornton & Ross Ltd and the Research Ethics Committee before the study can be continued.

##### Study Report

The Chief Investigator will submit a summary study report within approximately 2 months of completion of the study. This report will include:

1. Details of the investigative procedures involved.
2. The numbers of participants entered, completed, and withdrawn from the study.
3. Deviations from the study protocol on a general basis and for individual participants, with explanations.
4. Explanations for each participant withdrawn from the study.
5. Methodology and normal ranges for laboratory investigations (where appropriate)
6. Summary of the safety and tolerance data, including details of all Adverse Drug Events (ADE) including any follow-up. Case histories of all serious ADEs or ADEs leading to withdrawal should be provided.
7. If appropriate, details of any statistical analysis carried out by the Investigators, and a summary of efficacy data including clinical observations.
8. Conclusions

A copy of the report will be forwarded to the Research Ethics Committee.

## 4.7 Curriculum Vitae

In accordance with international standards, and Good Clinical Research Practice, a signed copy of the curriculum vitae of the Chief/Principal Investigators, Research Physician/Co-Investigator, Statistician and members of the MEC study team will be provided to Thornton & Ross Ltd.

## 4.8 Case Record Form

The Investigator is required to prepare and maintain adequate and accurate case records that have been approved by Thornton & Ross Ltd to record all observations and other data pertinent to the clinical study. All CRFs should be completed in their entirety in a neat, legible manner to ensure accurate interpretation of data. Black ballpoint pen should be used to ensure the clarity of reproduced copies. Any alterations or errors to the CRF should be crossed through once only, and signed and dated by the person making the change, using black ballpoint pen.

The study monitor will examine the original CRFs at each monitoring visit and will approve them when the CRF is complete and any necessary amendments have been made. The Investigator will not sign off the CRFs until the study monitor has approved them. The Investigator will retain the CRFs until completion of data collection when they will be given to the study monitor for transfer of data to Thornton & Ross Ltd and/or the sponsor’s approved statistician. The Investigator will retain a copy together with other source data for his/her own files.

The CPMP Guidelines on Good Clinical Practice for Trials on Medicinal Products in the European Community require that the Investigator shall arrange for the retention of the participant identification codes for at least 25 years after the completion or discontinuation of the study. Participant files and other source data shall also be kept for the maximum period permitted by the institution but not less than 25 years.

## 4.9 Monitoring of the Study

At regular intervals during the study, a representative of the monitoring team appointed by Thornton & Ross Ltd will visit the study centre. At each monitoring visit, the Investigator and the monitor will review study progress, compliance with the study protocol, CRF’s, and any emergent problems.

## 4.10 Quality Assurance

In accordance with Good Clinical Practice Guidelines and recommendations, Thornton & Ross Ltd may undertake an independent quality assurance audit of the clinical study and related documentation during the course of this study. The purpose of the audit is to determine whether the evaluated trial related activities were conducted, and the data were recorded, analysed and accurately reported according to the protocol, Thornton & Ross Ltd’s Standard Operating Procedures, Good Clinical Practice and the applicable regulatory requirements. At any stage during the study, the Investigator has the responsibility to make all data available to Thornton & Ross Ltd and/or relevant authority (where required) for auditing purposes. Such audits will at all times be conducted in accordance with national, legal and ethical requirements.

## 4.11 Protocol Appendices

It is specified that the appendices attached to this protocol, and referred to in the main text of this protocol, form an integral part of the protocol.

## 4.12 Protocol Amendments

Neither Thornton & Ross Ltd nor the Investigators may make any changes or amendments to this protocol, after the protocol has been agreed and signed by both parties, unless such change(s) or amendment(s) have been fully discussed and agreed by both the Investigator and Thornton & Ross Ltd. Any change or amendment agreed will be recorded in writing, the written amendment will be signed by the Investigator and by Thornton & Ross Ltd and the signed amendment will be appended to this protocol.

Any substantive changes will be forwarded to the Research Ethics Committee and to the appropriate regulatory authority for approval before implementation of the amendments.

## 4.13 Publication Policy

Submission of results for publication will not take place without prior discussion with Thornton & Ross Ltd, allowing the company sufficient time to analyse such results and provide written agreement to publication, which will not be unreasonably withheld. Thornton & Ross Ltd reserves the right to use the results and reports of this study for any purpose.

## 4.14 Early Termination of the Study

By agreement between Thornton & Ross Ltd and the Principal Investigators, the study may be terminated at any time if the recruitment rate is such that the required number of participants will not be recruited within the specified time, if the product being used is deemed to be failing unacceptably, or if any safety concerns arise.

5. Appendix 1: Declaration of Helsinki

**WORLD MEDICAL ASSOCIATION DECLARATION OF HELSINKI**

**Ethical Principles for Medical Research Involving Human Subjects**

Adopted by the 18th WMA General Assembly, Helsinki, Finland, June 1964, and amended by the:

29th WMA General Assembly, Tokyo, Japan, October 1975

35th WMA General Assembly, Venice, Italy, October 1983

41st WMA General Assembly, Hong Kong, September 1989

48th WMA General Assembly, Somerset West, Republic of South Africa, October 1996

52nd WMA General Assembly, Edinburgh, Scotland, October 2000

53th WMA General Assembly, Washington 2002 (Note of Clarification on paragraph 29 added)

55th WMA General Assembly, Tokyo 2004 (Note of Clarification on Paragraph 30 added)

59th WMA General Assembly, Seoul, October 2008

**A. INTRODUCTION**

1. The World Medical Association (WMA) has developed the Declaration of Helsinki as a statement of ethical principles for medical research involving human subjects**,** including research on identifiable human material and data. The Declaration is intended to be read as a whole and each of its constituent paragraphs should not be applied without consideration of all other relevant paragraphs.

2. Although the Declaration is addressed primarily to physicians, the WMA encourages other participants in medical research involving human subjects to adopt these principles.

3. It is the duty of the physician to promote and safeguard the health of patients, including those who are involved in medical research. The physician's knowledge and conscience are dedicated to the fulfilment of this duty.

4. The Declaration of Geneva of the WMA binds the physician with the words, “The health of my patient will be my first consideration,” and the International Code of Medical Ethics declares that, “A physician shall act in the patient's best interest when providing medical care.”

5. Medical progress is based on research that ultimately must include studies involving human subjects. Populations that are underrepresented in medical research should be provided appropriate access to participation in research.

6. In medical research involving human subjects, the well-being of the individual research subject must take precedence over all other interests.

7. The primary purpose of medical research involving human subjects is to understand the causes, development and effects of diseases and improve preventive, diagnostic and therapeutic interventions (methods, procedures and treatments). Even the best current interventions must be evaluated continually through research for their safety, effectiveness, efficiency, accessibility and quality.

8. In medical practice and in medical research, most interventions involve risks and burdens.

9. Medical research is subject to ethical standards that promote respect for all human subjects and protect their health and rights. Some research populations are particularly vulnerable and need special protection. These include those who cannot give or refuse consent for themselves and those who may be vulnerable to coercion or undue influence.

10. Physicians should consider the ethical, legal and regulatory norms and standards for research involving human subjects in their own countries as well as applicable international norms and standards. No national or international ethical, legal or regulatory requirement should reduce or eliminate any of the protections for research subjects set forth in this Declaration.

**B. PRINCIPLES FOR ALL MEDICAL RESEARCH**

11. It is the duty of physicians who participate in medical research to protect the life, health, dignity, integrity, right to self-determination, privacy, and confidentiality of personal information of research subjects.

12. Medical research involving human subjects must conform to generally accepted scientific principles, be based on a thorough knowledge of the scientific literature, other relevant sources of information, and adequate laboratory and, as appropriate, animal experimentation. The welfare of animals used for research must be respected.

13. Appropriate caution must be exercised in the conduct of medical research that may harm the environment.

14. The design and performance of each research study involving human subjects must be clearly described in a research protocol. The protocol should contain a statement of the ethical considerations involved and should indicate how the principles in this Declaration have been addressed. The protocol should include information regarding funding, sponsors, institutional affiliations, other potential conflicts of interest, incentives for subjects and provisions for treating and/or compensating subjects who are harmed as a consequence of participation in the research study. The protocol should describe arrangements for post-study access by study subjects to interventions identified as beneficial in the study or access to other appropriate care or benefits.

15. The research protocol must be submitted for consideration, comment, guidance and approval to a research ethics committee before the study begins. This committee must be independent of the researcher, the sponsor and any other undue influence. It must take into consideration the laws and regulations of the country or countries in which the research is to be performed as well as applicable international norms and standards but these must not be allowed to reduce or eliminate any of the protections for research subjects set forth in this Declaration. The committee must have the right to monitor ongoing studies. The researcher must provide monitoring information to the committee, especially information about any serious adverse events. No change to the protocol may be made without consideration and approval by the committee.

16. Medical research involving human subjects must be conducted only by individuals with the appropriate scientific training and qualifications. Research on patients or healthy volunteers requires the supervision of a competent and appropriately qualified physician or other health care professional. The responsibility for the protection of research subjects must always rest with the physician or other health care professional and never the research subjects, even though they have given consent.

17. Medical research involving a disadvantaged or vulnerable population or community is only justified if the research is responsive to the health needs and priorities of this population or community and if there is a reasonable likelihood that this population or community stands to benefit from the results of the research.

18. Every medical research study involving human subjects must be preceded by careful assessment of predictable risks and burdens to the individuals and communities involved in the research in comparison with foreseeable benefits to them and to other individuals or communities affected by the condition under investigation.

19. Every clinical trial must be registered in a publicly accessible database before recruitment of the first subject.

20. Physicians may not participate in a research study involving human subjects unless they are confident that the risks involved have been adequately assessed and can be satisfactorily managed. Physicians must immediately stop a study when the risks are found to outweigh the potential benefits or when there is conclusive proof of positive and beneficial results.

21. Medical research involving human subjects may only be conducted if the importance of the objective outweighs the inherent risks and burdens to the research subjects.

22. Participation by competent individuals as subjects in medical research must be voluntary. Although it may be appropriate to consult family members or community leaders, no competent individual may be enrolled in a research study unless he or she freely agrees.

23. Every precaution must be taken to protect the privacy of research subjects and the confidentiality of their personal information and to minimize the impact of the study on their physical, mental and social integrity.

24. In medical research involving competent human subjects, each potential subject must be adequately informed of the aims, methods, sources of funding, any possible conflicts of interest, institutional affiliations of the researcher, the anticipated benefits and potential risks of the study and the discomfort it may entail, and any other relevant aspects of the study. The potential subject must be informed of the right to refuse to participate in the study or to withdraw consent to participate at any time without reprisal. Special attention should be given to the specific information needs of individual potential subjects as well as to the methods used to deliver the information. After ensuring that the potential subject has understood the information, the physician or another appropriately qualified individual must then seek the potential subject’s freely-given informed consent, preferably in writing. If the consent cannot be expressed in writing, the non-written consent must be formally documented and witnessed.

25. For medical research using identifiable human material or data, physicians must normally

seek consent for the collection, analysis, storage and/or reuse. There may be situations where consent would be impossible or impractical to obtain for such research or would pose a threat to the validity of the research. In such situations the research may be done only after consideration and approval of a research ethics committee.

26. When seeking informed consent for participation in a research study the physician should be particularly cautious if the potential subject is in a dependent relationship with the physician or may consent under duress. In such situations the informed consent should be sought by an appropriately qualified individual who is completely independent of this relationship.

27. For a potential research subject who is incompetent, the physician must seek informed consent from the legally authorized representative. These individuals must not be included in a research study that has no likelihood of benefit for them unless it is intended to promote the health of the population represented by the potential subject, the research cannot instead be performed with competent persons, and the research entails only minimal risk and minimal burden.

28. When a potential research subject who is deemed incompetent is able to give assent to decisions about participation in research, the physician must seek that assent in addition to the consent of the legally authorized representative. The potential subject’s dissent should be respected.

29. Research involving subjects who are physically or mentally incapable of giving consent, for example, unconscious patients, may be done only if the physical or mental condition that prevents giving informed consent is a necessary characteristic of the research population. In such circumstances the physician should seek informed consent from the legally authorized representative. If no such representative is available and if the research cannot be delayed, the study may proceed without informed consent provided that the specific reasons for involving subjects with a condition that renders them unable to give informed consent have been stated in the research protocol and the study has been approved by a research ethics committee. Consent to remain in the research should be obtained as soon as possible from the subject or a legally authorized representative.

30. Authors, editors and publishers all have ethical obligations with regard to the publication of the results of research. Authors have a duty to make publicly available the results of their research on human subjects and are accountable for the completeness and accuracy of their reports. They should adhere to accepted guidelines for ethical reporting. Negative and inconclusive as well as positive results should be published or otherwise made publicly available. Sources of funding, institutional affiliations and conflicts of interest should be declared in the publication. Reports of research not in accordance with the principles of this Declaration should not be accepted for publication.

**C. ADDITIONAL PRINCIPLES FOR MEDICAL RESEARCH COMBINED WITH MEDICAL CARE**

31. The physician may combine medical research with medical care only to the extent that the research is justified by its potential preventive, diagnostic or therapeutic value and if the physician has good reason to believe that participation in the research study will not adversely affect the health of the patients who serve as research subjects.

32. The benefits, risks, burdens and effectiveness of a new intervention must be tested

against those of the best current proven intervention, except in the following circumstances:

• The use of placebo, or no treatment, is acceptable in studies where no current proven intervention exists; or

• Where for compelling and scientifically sound methodological reasons the use of placebo is necessary to determine the efficacy or safety of an intervention and the patients who receive placebo or no treatment will not be subject to any risk of serious or irreversible harm. Extreme care must be taken to avoid abuse of this option.

33. At the conclusion of the study, patients entered into the study are entitled to be informed about the outcome of the study and to share any benefits that result from it, for example, access to interventions identified as beneficial in the study or to other appropriate care or benefits.

34. The physician must fully inform the patient which aspects of the care are related to the research. The refusal of a patient to participate in a study or the patient’s decision to withdraw from the study must never interfere with the patient-physician relationship.

35. In the treatment of a patient, where proven interventions do not exist or have been ineffective, the physician, after seeking expert advice, with informed consent from the patient or a legally authorized representative, may use an unproven intervention if in the physician's judgement it offers hope of saving life, re-establishing health or alleviating suffering. Where possible, this intervention should be made the object of research, designed to evaluate its safety and efficacy. In all cases, new information should be recorded and, where appropriate, made publicly available.

Appendix 2: ICH guidelines on Good Clinical Practice

**Responsibilities of the Investigator**

**Investigator’s Qualifications and Agreements**

1. The investigator(s) should be qualified by education, training, and experience to assume responsibility for the proposed conduct of the trial, should meet all the qualifications specified by the applicable regulatory requirement(s), and should provide evidence of such qualifications through up-to-date curriculum vitae and/or other relevant documentation requested by the sponsor, the IRB/IEC, and/or the regulatory authority(ies).

2. The investigator should be thoroughly familiar with the appropriate use of the investigational product(s), as described in the protocol, in the current Investigator’s Brochure, in the product information and in other information sources provided by the sponsor.

3. The investigator should be aware of, and should comply with, GCP and the applicable regulatory requirements.

4. The investigator/institution should permit monitoring and auditing by the sponsor, and inspection by the appropriate regulatory authority(ies).

5. The investigator should maintain a list of appropriately qualified persons to whom the investigator has delegated significant trial-related duties.

**Adequate Resources**

1. The investigator should be able to demonstrate (e.g. based on retrospective data) a potential for recruiting the required number of suitable subjects with the agreed recruitment period.

2. The investigator should have sufficient time to properly conduct and complete the trial within the agreed trial period.

3. The investigator should have available an adequate number of qualified staff and adequate facilities for the foreseen duration of the trial to conduct the trial properly and safely.

4. The investigator should ensure that all persons assisting with the trial are adequately informed about the protocol, the investigational product(s), and their trial-related duties and functions.

**Medical Care of Trial Subjects**

1. A qualified physician (or dentist, when appropriate), who is an investigator or a sub-investigator for the trial, should be responsible for all trial-related medical (or dental) decisions.

2. During and following a subject’s participation in a trial, the investigator/institution should ensure that adequate medical care is provided to a subject for any adverse events, including clinically significant laboratory values, related to the trial. The investigator/institution should inform a subject when medical care is needed for intercurrent illness(es) of which the investigator becomes aware.

3. It is recommended that the investigator inform the subject’s primary physician about the subject’s participation in the trial if the subject has a primary physician and if the subject agrees to the primary physician being informed.

4. Although a subject is not obliged to give his/her reason(s) for withdrawing prematurely from a trial, the investigator should make a reasonable effort to ascertain the reason(s), while fully respecting the subject’s rights.

**Communication with IRB/IEC**

1. Before initiating a trial, the investigator/institution should have written and dated approval/favourable opinion from the IRB/IEC for the trial protocol, written informed consent form, consent form updates, subject recruitment procedures (e.g. advertisements) and any other written information to be provided to subjects.

2. As part of the investigator’s/institution’s written application to the IRB/IEC, the investigator/institution should provide the IRB/IEC with a current copy of the Investigator’s Brochure. If the Investigator’s Brochure is updated during the trial, the investigator/institution should supply a copy of the updated Investigator’s Brochure to the IRB/IEC.

3. During the trial, the investigator/institution should provide to the IRB/IEC all documents subject to review.

**Compliance with Protocol**

1. The investigator/institution should conduct the trial in compliance with the protocol agreed to by the sponsor and, if required, by the regulatory authority(ies) and which was given approval/favourable opinion by the IRB/IEC. The investigator/institution and the sponsor should sign the protocol, or an alternative contract, to confirm agreement.

2. The investigator should not implement any deviation from, or changes of the protocol without agreement by the sponsor and prior review and documented approval/ favourable opinion from the IRB/IEC of an amendment, except where necessary to eliminate an immediate hazard(s) to trial subjects, or when the change(s) involves only logistical or administrative aspects of the trial (e.g. change in monitor(s), change of telephone number(s).

3. The investigator, or person designated by the investigator, should document and explain any deviation from the approved protocol.

4. The investigator may implement a deviation from, or a change of, the protocol to eliminate an immediate hazard(s) to trial subjects without prior IRB/IEC approval/ favourable opinion. As soon as possible, the implemented deviation or change, the reasons for it, and, if appropriate, the proposed protocol amendment(s) should be submitted:

1. to the IRB/IEC for review and approval/favourable opinion,
2. to the sponsor for agreement and, if required,
3. to the regulatory authority(ies).

**Investigational Product(s)**

1. Responsibility for investigational product(s) accountability at the trial site(s) rests with the investigator/institution.

2. Where allowed/required, the investigator/institution may/should assign some or all the investigator’s/institution’s duties for investigational product(s) accountability at the trial site(s) to an appropriate pharmacist or another appropriate individual who is under the supervision of the investigator/institution.

3. The investigator/institution and/or a pharmacist or other appropriate individual, who is designated by the investigator/institution should maintain records of the product’s delivery to the trial site, the inventory at the site, the use by each subject, and the return to the sponsor or alternative disposition of unused product(s). These records should include dates, quantities, batch/serial numbers, expiration dates (if applicable), and the unique code numbers assigned to the investigational product(s) and trial subjects. Investigators should maintain records that document adequately that the subjects were provided the doses specified by the protocol and reconcile all investigational product(s) received from the sponsor.

4. The investigational product(s) should be stored as specified by the sponsor and in accordance with applicable regulatory requirement(s).

5. The investigator should ensure that the investigational product(s) are used only in accordance with the approved protocol.

6. The investigator, or a person designated by the investigator/institution, should explain the correct use of the investigational product(s) to each subject and should check, at intervals appropriate for the trial, that each subject is following the instructions properly.

**Randomisation Procedures and Unblinding**

1. The investigator should follow the trial’s randomisation procedures, if any, and should ensure that the code is broken only in accordance with the protocol. If the trial is blinded, the investigator should promptly document and explain to the sponsor any premature unblinding (e.g. accidental unblinding, unblinding due to a serious adverse event) of the investigational product(s).

**Informed Consent of Trial Subjects**

1. In obtaining and documenting informed consent, the investigator should comply with the applicable regulatory requirement(s), and should adhere to GCP and to the ethical principles that have their origin in the Declaration of Helsinki. Before the beginning of the trial, the investigator should have the IRB/IEC’s written approval/favourable opinion of the written informed consent form and any other written information to be provided to subjects.

2. The written informed consent form and any other written information to be provided to subjects should be revised whenever important new information becomes available that may be relevant to the subject’s consent. Any revised written informed consent form, and written information should receive the IRB/IEC’s approval/favourable opinion in advance of use. The subject or the subject’s legally acceptable representative should be informed in a timely manner if new information becomes available that may be relevant to the subject’s willingness to continue participation in the trial. The communication of this information should be documented.

3. Neither the investigator, nor the trial staff, should coerce or unduly influence a subject to participate or to continue to participate in a trial.

4. None of the oral and written information concerning the trial, including the written informed consent form, should contain any language that causes the subject or the subject’s legally acceptable representative to waive or to appear to waive any legal rights, or that releases or appears to release the investigator, the institution, the sponsor, or their agents from liability for negligence.

5. The investigator, or a person designated by the investigator, should fully inform the subject or, if the subject is unable to provide informed consent, the subject’s legally acceptable representative, of all pertinent aspects of the trial including the written information given approval/favourable opinion by the IRB/IEC.

6. The language used in the oral and written information about the trial, including the written informed consent form, should be as non-technical as practical and should be understandable to the subject or the subject’s legally acceptable representative and the impartial witness, where applicable.

7. Before informed consent may be obtained, the investigator, or a person designated by the investigator, should provide the subject or the subject’s legally acceptable representative ample time and opportunity to enquire about details of the trial and to decide whether or not to participate in the trial. All questions about the trial should be answered to the satisfaction of the subject or the subject’s legally acceptable representative.

8. Prior to a subject’s participation in the trial, the written informed consent form should be signed and personally dated by the subject or the subject’s legally acceptable representative, and by the person who conducted the informed consent discussion.

9. If a subject is unable to read or if a legally acceptable representative is unable to read, an impartial witness should be present during the entire informed consent discussion. After the written informed consent form and any other written information to be provided to subjects, is read and explained to the subject or the subject’s legally acceptable representative, and after the subject or the subject’s legally acceptable representative has orally consented to the subject’s participation in the trial and, if capable of doing so, has signed and personally dated the informed consent form, the witness should sign and personally date the consent form. By signing the consent form, the witness attests that the information in the consent form and any other written information was accurately explained to, and apparently understood by, the subject or the subject’s legally acceptable representative, and that informed consent was freely given by the subject or the subject’s legally acceptable representative.

10. Both the informed consent discussion and the written informed consent form and any other written information to be provided to subjects should include explanation of the following:

1. That the trial involves research.
2. The purpose of the trial.
3. The trial treatment(s) and the probability for random assignment to each treatment.
4. The trial procedures to be followed, including all invasive procedures.
5. The subject’s responsibilities.
6. Those aspects of the trial that are experimental.
7. The reasonably foreseeable risks or inconveniences to the subject and, when applicable, to an embryo, foetus, or nursing infant.
8. The reasonably expected benefits. When there is no intended clinical benefit to the subject, the subject should be made aware of this.
9. The alternative procedure(s) or course(s) of treatment that may be available to the subject, and their important potential benefits and risks.
10. The compensation and/or treatment available to the subject in the event of trial-related injury.
11. The anticipated prorated payment, if any, to the subject for participating in the trial.
12. The anticipated expenses, if any, to the subject for participating in the trial.
13. That the subject’s participation in the trial is voluntary and that the subject may refuse to participate or withdraw from the trial, at any time, without penalty or loss of benefits to which the subject is otherwise entitled.
14. That the monitor(s), the auditor(s), the IRB/IEC, and the regulatory authority(ies) will be granted direct access to the subject’s original medical records for verification of clinical trial procedures and/or data, without violating the confidentiality of the subject, to the extent permitted by the applicable laws and regulations and that, by signing a written informed consent form, the subject or the subject’s legally acceptable representative is authorising such access.
15. That records identifying the subject will be kept confidential and, to the extent permitted by the applicable laws and/or regulations, will not be made publicly available. If the results of the trial are published, the subject’s identity will remain confidential.
16. That the subject or the subject’s legally acceptable representative will be informed in a timely manner if information becomes available that may be relevant to the subject’s willingness to continue participation in the trial.
17. The person(s) to contact for further information regarding the trial and the rights of trial subjects, and whom to contact in the event of trial-related injury.
18. The foreseeable circumstances and/or reasons under which the subject’ s participation in the trial may be terminated.
19. The expected duration of the subject’s participation in the trial.
20. The approximate number of subjects involved in the trial.

11. Prior to participation in the trial, the subject or the subject’s legally acceptable representative should receive a copy of the signed and dated written informed consent form and any other written information provided to the subjects. During a subject’s participation in the trial, the subject or the subject’s legally acceptable representative should receive a copy of the signed and dated consent form updates and a copy of any amendments to the written information provided to subjects.

12. When a clinical trial (therapeutic or non-therapeutic) includes subjects who can only be enrolled in the trial with the consent of the subject’s legally acceptable representative (e.g. minors, or participants with severe dementia), the subject should be informed about the trial to the extent compatible with the subject’s understanding and, if capable, the subject should sign and personally date the written informed consent.

13. Except as described in 14, a non-therapeutic trial (i.e. a trial in which there is no anticipated direct clinical benefit to the subject), should be conducted in subjects who personally give consent and who sign and date the written informed consent form.

14. Non-therapeutic trials may be conducted in subjects with consent of a legally acceptable representative provided the following conditions are fulfilled:

1. The objectives of the trial cannot be met by means of a trial in subjects who can give informed consent personally.
2. The foreseeable risks to the subjects are low.
3. The negative impact on the subject’s well being is minimised and low.
4. The trial is not prohibited by law.
5. The approval/favourable opinion of the IRB/IEC is expressly sought on the inclusion of such subjects, and the written approval/favourable opinion covers this aspect.

Such trials, unless an exception is justified, should be conducted in participants having a disease or condition for which the investigational product is intended. Subjects in these trials should be particularly closely monitored and should be withdrawn if they appear to be unduly distressed.

15. In emergency situations, when prior consent of the subject is not possible, the consent of the subject’s legally acceptable representative, if present, should be requested. When prior consent of the subject is not possible, the subject’s legally acceptable representative is not available, enrolment of the subject should require measures described in the protocol and/or elsewhere, with documented approval/favourable opinion by the IRB/IEC, to protect the rights, safety and well being of the subject and to ensure compliance with applicable regulatory requirements. The subject or the subject’s legally acceptable representative should be informed about the trial as soon as possible and consent to continue and other consent as appropriate (see 10) should be requested.

**Records and Reports**

1. The investigator should ensure the accuracy, completeness, legibility, and timeliness of the data reported to the sponsor in the CRFs and in all required reports.

2. Data reported on the CRF that are derived from source documents should be consistent with the source documents or the discrepancies should be explained.

3. Any change or correction to a CRF should be dated, initialled, and explained (if necessary) and should not obscure the original entry (i.e. an audit trail should be maintained); this applies to both written and electronic changes or corrections. Sponsors should provide guidance to investigators and/or the investigator’s designated representatives on making such corrections.

Sponsors should have written procedures to assure that changes or corrections in CRFs made by sponsor’s designated representatives are documented, are necessary and are endorsed by the investigator. The investigator should retain records of the changes and corrections.

4. The investigator/institution should maintain the trial documents as specified in Essential Documents for the Conduct of a Clinical Trial and as required by the applicable regulatory requirement(s). The investigator/institution should take measures to prevent accidental or premature destruction of these documents.

5. Essential documents should be retained until at least 2 years after the last approval of a marketing application in an ICH region and until there are no pending or contemplated marketing applications in an ICH region or at least 2 years have elapsed since the formal discontinuation of clinical development of the investigational product. These documents should be retained for a longer period however if required by the applicable regulatory requirements or by an agreement with the sponsor. It is the responsibility of the sponsor to inform the investigator/institution as to when these documents no longer need to be retained.

6. The financial aspects of the trial should be documented in an agreement between the sponsor and the investigator/institution.

7. Upon request of the monitor, auditor, IRB/IEC or regulatory authority, the investigator/institution should make available for direct access all requested trial-related records.

**Progress Reports**

1. The investigator should submit written summaries of the trial status to IRB/IEC annually, or more frequently, if requested by the IRB/IEC.

2. The investigator should promptly provide written reports to the sponsor, the IRB/IEC and, where applicable, the institution on any changes significantly affecting the conduct of the trial, and/or increasing the risk to subjects.

**Safety Reporting**

1. All serious adverse events (SAEs) should be reported immediately to the sponsor except for those SAEs that the protocol or other document (e.g. Investigator’s Brochure) identifies as not needing immediate report. The immediate reports should be followed promptly by detailed, written reports. The immediate and follow-up reports should identify subjects by unique code numbers assigned to the trial subjects rather than by the subjects’ names, personal identification numbers and/or addresses. The investigator should also comply with the applicable regulatory requirement(s) related to the reporting of unexpected serious adverse drug reactions to the regulatory authority(ies) and the IRB/IEC.

2. Adverse events and/or laboratory abnormalities identified in the protocol as critical to safety evaluations should be reported to the sponsor according to the reporting requirements and within the time periods specified by the sponsor in the protocol.

3. For reported deaths, the investigator should supply the sponsor and the IRB/IEC with any additional requested information (e.g. autopsy reports and terminal medical reports).

**Premature Termination or Suspension of a Trial**

1. If the trial is prematurely terminated or suspended for any reason, the investigator/ institution should promptly inform the trial subjects, should assure appropriate therapy and follow-up for the subjects, and, where required by the applicable regulatory requirement(s), should inform the regulatory authority(ies). In addition to:

a) If the investigator terminates or suspends a trial without prior agreement of the sponsor, the investigator should inform the institution where applicable, and the investigator/institution should promptly inform the sponsor and the IRB/IEC, and should provide the sponsor and the IRB/IEC a detailed written explanation of the termination or suspension.

b) If the sponsor terminates or suspends a trial, the investigator should promptly inform the institution where applicable and the investigator/institution should promptly inform the IRB/IEC and provide the IRB/IEC a detailed written explanation of the termination or suspension.

c) If the IRB/IEC terminates or suspends its approval/favourable opinion of a trial, the investigator should inform the institution where applicable and the investigator/institution should promptly notify the sponsor and provide the sponsor with a detailed written explanation of the termination or suspension.

**Final Report(s) by Investigator**

1. Upon completion of the trial, the investigator, where applicable, should inform the institution; the investigator/institution should provide the IRB/IEC with a summary of the trial’s outcome, and the regulatory authority(ies) with any reports required.

1. Although 6 months is listed as a minimum age, in line with the proposed product labelling, it is not anticipated that any child below the age of 2 years is likely to be enrolled during this study. [↑](#footnote-ref-1)
2. See footnote 1 under “Design in brief” [↑](#footnote-ref-2)
