# Supplementary material for: 1,2-Octanediol, a Novel Surfactant, for Treating Head Louse Infestation: Identification of Activity, Formulation, and Randomised, Controlled Trials
Source: PLoS One. 2012 Apr 16;7(4):e35419. doi: 10.1371/journal.pone.0035419 (PMC3327678; doi:10.1371/journal.pone.0035419)
Supplement: Table S1 — GC-MS recovery of hydrocarbons from lice using eicosane as the reference standard. (DOCX) [file pone.0035419.s007.docx]

Table S1. GC-MS recovery of hydrocarbons from lice using eicosane as the reference standard

| **Identification** | **Retention Time (mins)** | **Pre-treatment** | | **Post-treatment** | |
| --- | --- | --- | --- | --- | --- |
|  |  | **Area** | **%w/w** | **Area** | **%w/w** |
| Eicosane (C20) | 13.8 | 9800211 | n/a | 10284168 | n/a |
| C25 | 18.4 | 1894774 | 0.012 | 245041 | 0.0015 |
| C27 | 19.9 | 2961454 | 0.019 | 633298 | 0.0039 |
| C29 | 21.4 | 6182866 | 0.040 | 2024795 | 0.012 |
| Total | n/a | n/a | 0.071 | n/a | 0.017 |

After treatment with 1,2-octanediol the quantities of the three principal *n*-alkanes extracted from the louse cuticular lipid were reduced to between approximately 25% and 10% of the pre-treatment quantities by weight.
