# Supplementary material for: 1,2-Octanediol, a Novel Surfactant, for Treating Head Louse Infestation: Identification of Activity, Formulation, and Randomised, Controlled Trials
Source: PLoS One. 2012 Apr 16;7(4):e35419. doi: 10.1371/journal.pone.0035419 (PMC3327678; doi:10.1371/journal.pone.0035419)
Supplement: Table S2 — GC-MS output peak areas covering >1% of the total lipid extract. (DOCX) [file pone.0035419.s008.docx]

Table S2. GC-MS output peak areas covering >1% of the total lipid extract

| Retention Time (mins) | Area% | | Compound |
| --- | --- | --- | --- |
|  | Pre-treatment | Post-treatment |  |
| 4.2 | - | 3.4 | Octanoic acid |
| 5.0 | - | 17.2 | Cyclic aliphatic |
| 5.7 | 1.1 | - | Low molecular weight aliphatic |
| 6.8 | 7.7 | - | Aliphatic Acid |
| 15.2 | 3 | - | Palmidrol |
| 17.1 | 1.3 | - | Palmidrol derivative |
| 18.4 | 7.6 | 1.6 | Pentacosane |
| 20.0 | 11.8 | 3.8 | Heptacosane |
| 20.7 | 2.1 | - | Substituted saturated aliphatic |
| 21.3 | 5.2 | - | Unsaturated aliphatic |
| 21.4 | 24.8 | 12.3 | Nonacosane |
| 21.7 | 7.3 | - | Substituted saturated aliphatic, possibly amine |
| 22.7 | 9.7 | - | Substituted unsaturated aliphatic |
| 23.1 | 4.8 | - | Substituted saturated aliphatic |
| 23.3 | 4.6 | - | Substituted saturated aliphatic |
