# Supplementary material for: 1,2-Octanediol, a Novel Surfactant, for Treating Head Louse Infestation: Identification of Activity, Formulation, and Randomised, Controlled Trials
Source: PLoS One. 2012 Apr 16;7(4):e35419. doi: 10.1371/journal.pone.0035419 (PMC3327678; doi:10.1371/journal.pone.0035419)
Supplement: Text file S1 — Preliminary work investigating the activity of 1,2-octandiol on cuticular lipids of human lice. (DOCX) [file pone.0035419.s009.docx]

Preliminary investigation of the activity of 1,2-octandiol on cuticular lipids of human lice.

The first stage in the process of determining whether 1,2-octanediol could have any effect on the surface lipids of head lice was to confirm that 1,2-octanediol solution adequately covered the surface of lice. This was confirmed by treating insects in the laboratory using a version of the alcohol free 1,2-octanediol formulation dosed with 1% zinc oxide nanopowder (< 100 nm) (Sigma Aldrich) and made into a suspension. The lice were treated as they would be for a normal *ex vivo* efficacy test and then examined using an SXL30 Environmental SEM in environmental mode with a pressure of 65 Pascal of water for charge control. X-ray elemental mapping was performed using a 20 keV beam scanning for zinc which shows up as the yellow patterning in the scan (Figure S1b), which has been superimposed onto the SEM photo image of the louse (Figure S1a). These images show a spread of the 1,2-octanediol formulation across the surface of the louse but, as might be expected, there were accumulations of the fluid in folds and clefts in the cuticle surface.

The lipid coating of insect cuticle has been investigated for several species from widely separated taxonomic groups living in a range of ecological environments. The majority of components of cuticular lipid have been shown as *n*-alkanes or *n*-alkenes [1] ranging from 98% of total lipid [2], down to only trace quantities in some tsetse flies (*Glossina* spp) [3,4], or even absent in the Colorado potato beetle, *Leptinotarsa decemlineata* [5]. In addition the cuticle lipids of most insects also contain a small percentage of branched alkanes, wax esters, and other compounds [1].

The cuticular lipids of insects from the Psocodea have not been investigated other than one study of the psocid *Liposcelis bostrychophila* [6]. In this study it was found that the cuticle lipid was unlike that of many other species with a core of C21–C34 *n*-alkanes, C28–C42 monomethyl alkanes and C31–C43 dimethyl alkanes, but no unsaturated hydrocarbons. All of these were only present in small quantities, the most abundant being C29 and C31 *n*-alkanes, a mix of C31 monomethyl alkanes, and C33 mono- and dimethyl alkanes. None of these constituted more than about 10% of the total lipid. Fatty acid amides constituted a homologous series (C16–C22 in chain length), with stearoyl amide (C17) being the most common [6].

A similar pattern of *n*-alkanes, the most prevalent being C29-C33, together with lower proportions of mono- and dimethyl alkanes were also found in the blood feeding bugs of the sub-family Triatominae, although in this group the fatty acid amides were not identified [7].

We have conducted a preliminary investigation and partial analysis of the cuticular lipids of human head lice, *Pediculus capitis*, by hexane extraction. Initial experiments using gas chromatography showed a series of peaks equivalent to *n*-alkanes with C17, C19, C25, C26 and C27 chains. For treated lice the same alkanes could be identified with further peaks in the range C28-C32. Additional tests were then conducted using GC-MS to examine some of the peaks and the effect on these as a result of exposure to 5% 1,2-octanediol solution.

For analysis of cuticle lipids in untreated insects, 50 lice were accurately weighed into a 5 mL vial and 250 µL of 0.005% eicosane (C20) in hexane added. The vial was swirled for approximately 2 minutes and the resulting solution, with its internal standard of eicosane, decanted off into a gas chromatograph vial containing a microlitre insert. The resultant solution was analysed by GC-MS.

For the post-treatment sample, a further 50 lice were accurately weighed into a 5 mL vial and 1 mL of 5% 1,2-octanediol preparation added and the vial swirled. These were left to stand at room temperature overnight before being washed with a 1% solution of sodium dodecyl sulphate, rinsed with ultrapure water, and dried in an oven at 40 °C. The lice were then extracted with the standard eicosane in hexane solution and processed in the same way as the untreated lice.

Analytes extracted from the untreated lice consisted of a number of long chain saturated hydrocarbons, up to C29, with the most common being C25, C27, and C29 (Table S1). The GC-MS also detected a range of saturated and unsaturated substituted aliphatics from C12 to approximately C20, in which the possible substitutions were amide, alcohol, or acid groups. Two low molecular weight analytes were also detected early in the chromatogram (Figure S2, Table S2). After washing with 5% 1,2-octanediol the weight for weight (w/w) percentage of hexane extracted hydrocarbons C25, C27 and C29 from the known weight of head lice was found to decrease from 0.07% w/w to 0.02% w/w (Table S1). The range of analytes detected also decreased (Figure S3, Table S2).

Analysis by GC-MS of louse cuticular waxes before and after treatment with 1,2-octanediol suggests that it is able to disrupt the integrity of the alkane components in the C25-C29 range such that they can be emulsified and removed using a weak sodium dodecyl sulphate solution. This is consistent with observations made in experiments investigating the activity of 1,2-octanediol against aphids on roses. Cultivated rose leaves have a high proportion of C25-C33 alkanes in their epicuticular waxes [8,9], which protect the leaf tissues from excessive water loss and dehydration. Those leaves exposed to a 1,2-octanediol preparation exhibited wilt and “leaf burn” after treatment, apparently as a result of dehydration because the leaf cuticle lipids were disrupted (EctoPharma Ltd, unpublished data). Consequently, these observations provide evidence that 1,2-octanediol acts against human head lice by cuticular lipid disruption leading to excessive water loss and subsequent death through dehydration.

Acknowledgements

Environmental SEM work was performed by Lech Staniewicz, Cavendish Laboratory, University of Cambridge; preliminary lipid extractions and gas chromatography by Steve Valovin and Insect R&D Limited, Cambridge; and GC-MS work by Helen Winsor, IPOS research group, University of Huddersfield.

References

1. Blomquist, G.J., Nelson, D.R., de Renobales, M. 1987. Chemistry, biochemistry, and physiology of insect cuticular lipids. Archives of Insect Biochemistry and Physiology 6, 227-265.

2. Lockey, K.H. 1988. Lipids of the insect cuticle: origin, composition and function. Comparative Biochemistry and Physiology [B] 89, 595–645.

3. Nelson, D.R., Carlson, D.A. 1986. Cuticular hydrocarbons of the tsetse flies *Glossina morsitans morsitans, G. austeni* and *G. pallidipes.* Insect Biochemistry 26, 403-416.

4. Nelson, D.R., Carlson, D.A., Fatland, C.L. 1988. Cuticular hydrocarbons of the tsetse flies, II *G. f. fuscipes, G. p. palpalis, G. p. garnbiensis, G. tachinoides* and *G. bratipalpis.* Journal of Chemical Ecology, [14,](http://www.springerlink.com/content/0098-0331/14/3/) 963-987.

5. Malinski, E., Kusmierz, J., Szafranek, J., Dubis, E., Poplawski, J., Wrobel, J.T., Konig, W.A. 1986. Cuticular hydrocarbons of the Colorado beetle *Leptinotarsa decemlineata* Say. Zeitschrift fur Naturforschung B 41, 567-574

6. Howard, R.W., Lord, J.C. 2003. Cuticular lipids of the booklouse, *Liposcelis bostrichophila* hydrocarbons, aldehydes, fatty acids, and fatty acid amides. Journal of Chemical Ecology, 29,615-627.

7. Juárez, M.P., Carlson, D.A., Schettino, P.M.S., Mijailovsky, S., Rojas, G. 2002. Cuticular hydrocarbons of Chagas disease vectors in Mexico. Memorias do Instituto Oswaldo Cruz*,* Rio de Janeiro, 97, 819-827.

8. Buschhaus, C., Herz, H., Jetter R. 2007. Chemical composition of the epicuticular and intracuticular wax layers on adaxial sides of *Rosa canina* leaves. Annals of Botany 100, 1557–1564.

9. Goodwin, S.M., Edwards, C.J., Jenks, M.A. 2007. Leaf cutin monomers, cuticular waxes, and blackspot resistance in rose. Horticultural Science 42, 1631–1635.
